# Decoding Sulfur-Containing Aroma Compounds in Foods: From Key Odorant Mapping to Structure–Odor Mechanisms and Flavor Design

**DOI:** 10.3390/foods15173003

**Published:** 2026-08-26

**Authors:** Jinpeng Hu, Lulu Ma, Jiaying Huo, Jinyuan Sun, Shugang Li, Hao Wang

**Affiliations:** 1 School of Food and Biological Engineering, Engineering Research Center of Bio-Process, Ministry of Education, Key Laboratory for Agricultural Products Processing of Anhui Province, Hefei University of Technology, Hefei 230601, China; hujinpeng06@163.com (J.H.); 2022800057@hfut.edu.cn (L.M.); huojy2023@hfut.edu.cn (J.H.); 2 China Food Flavor and Nutrition Health Innovation Center, Beijing Technology and Business University, Beijing 100048, China; sunjinyuan@btbu.edu.cn; 3 Institute of Agro-Products Processing Research of Anhui Academy of Agricultural Sciences, Hefei 230031, China; 4 Anhui Engineering Laboratory of Food Microbial Fermentation and Functional Application, Hefei 230031, China

**Keywords:** sulfur-containing aroma compounds, key food odorants, olfactory receptors, structure–odor relationship, flavor design

## Abstract

With extremely low odor thresholds and potent flavor activity, sulfur-containing aroma compounds (SACs) constitute the molecular cornerstone of characteristic flavors in meat, coffee, and fermented foods. Research has advanced from early component identification to the elucidation of structure–activity relationships, olfactory receptor recognition mechanisms, and food-flavor improvement. This review first summarizes the detection and quantification methods for SACs, their distribution in foods, key odor contributions, and major formation pathways. It then highlights progress in understanding molecular structural parameters, olfactory receptor recognition, and computational simulations that decode flavor perception mechanisms. From a translational perspective, we further discuss flavor retention in real food matrices, off-flavor regulation, cross-modal perceptual enhancement, and functional applications. Current challenges include food matrix complexity, high compound reactivity, and nonlinear olfactory combinatorial coding. Future directions involve constructing a multiscale predictive framework integrating neuroscience, developing explainable artificial intelligence to decode olfactory coding, and advancing closed-loop green biomanufacturing for the precise design and sustainable production of SACs.

## 1. Introduction

Flavor is the core attribute determining food quality and consumer acceptance, and its essence is the integrated olfactory perception formed when groups of volatile compounds stimulate olfactory receptors [1]. Among the tens of thousands of food volatiles identified, fewer than 3% are recognized as key food odorants (KFOs) because their concentrations exceed their sensory thresholds, thus dominating the characteristic aroma of foods [2]. Among KFOs, sulfur-containing aroma compounds (SACs) occupy a special position due to their exceptionally high flavor impact: although they account for only about 10% of total volatile substances, they represent up to 16% of the 226 established KFOs, making them one of the most representative categories [3]. This prominent status stems from their universally extremely low odor thresholds (often down to ng/L or µg/L levels) and their intense, distinctive sensory characteristics (e.g., meaty, garlic-like, fruity notes), rendering them key contributors that shape the characteristic flavor profiles of numerous food systems ranging from fruits and vegetables to meats and fermented products [4].

SACs have long been a hot topic in food flavor chemistry [5], and with the advancement of research techniques, the SACs research paradigm has undergone a profound shift from macro-level phenomenon description to micro-level mechanism elucidation. Early studies achieved a breakthrough from observing aroma generation upon heating to pinpointing key SACs, relying on gas chromatography-mass spectrometry (GC-MS) to identify landmark compounds such as 2-methyl-3-furanthiol and its disulfide [6]. Entering the 21st century, the research focus shifted from discovering what they are to understanding why they are. Baoguo Sun’s team proposed a structural model for meaty aroma molecules (e.g., 1,2-oxo/thio functional groups) [7], preliminarily establishing a qualitative relationship between molecular structure and sensory attributes. Subsequently, studies by Schieberle and coworkers [8,9,10,11,12] entered the quantitative structure–activity relationship (QSAR) phase, systematically revealing for the first time the precise influence of structural parameters on odor thresholds, thereby driving the research deeper from “macroscopic rule summarization” toward “molecular-level structure–activity relationship elucidation and olfactory recognition mechanism analysis”.

Building on these molecular-level insights, a series of specialized reviews have systematically catalogued the occurrence, formation, analysis, and sensory roles of volatile sulfur compounds across diverse food systems: baijiu [4,13], beverages (with a focus on 2-furfurylthiol) [14], vegetables and mushrooms [15], Allium plants [16], garlic [17], fermented products (cheese, wine, beer) [18], cooked meat [19], seafood [20], and thiols as key food odorants [21]. These works collectively establish a solid knowledge base on precursor chemistry, enzymatic and thermal generation pathways, and extraction/detection methodologies.

However, SACs research still faces a fundamental bottleneck: existing studies largely elucidate molecular mechanisms in simplified systems (pure compounds or model solutions), whereas the flavor performance of SACs in real foods results from the coupling of multiscale processes—molecular structure, receptor recognition, food matrix interactions, and neural coding. Proteins, lipids, and carbohydrates in food matrices can alter the release kinetics and olfactory accessibility of SACs through non-covalent or covalent interactions. Meanwhile, SACs not only directly activate olfactory receptors but can also allosterically modulate taste receptors (e.g., T1R1/T1R3) or trigger cross-modal enhancement effects (such as odor-induced saltiness enhancement, OISE) [22,23,24], reshaping overall flavor perception at the receptor level. This complexity, spanning from molecules to systems, demands that the research paradigm shift from isolated structural elucidation toward integrative multiscale prediction.

Unlike previous matrix-specific or compound-specific reviews, this review takes key odorant mapping, mechanism decoding, and flavor design as the main thread to summarize recent advances in SACs research. It begins with a systematic mapping of 54 key SACs across 221 food samples, providing an integrated view of their distribution patterns and odor contributions in diverse food systems. It then decodes the structure–odor mechanisms of SACs by tracing the progression from empirical associations between molecular structure and odor quality, to quantitative structure–threshold relationships, olfactory receptor recognition, and computational chemistry. On this basis, the review further discusses how these molecular mechanisms can be translated into flavor modulation and design in real food matrices, including flavor retention, off-flavor masking, cross-modal sensory enhancement, and functional applications. Finally, it highlights future directions involving multiscale prediction, explainable artificial intelligence, and green biomanufacturing, providing a theoretical framework for developing predictable and designable SACs-based food flavor systems.

## 2. Key SACs in Foods

SACs are bioactive volatiles that fundamentally define the flavor identity of diverse food systems. However, their high chemical reactivity, thermal lability, and matrix-dependent release behavior pose substantial challenges to accurate qualitative and quantitative analysis, key odorant identification, and formation pathway elucidation. This section systematically reviews research progress on foodborne SACs across three progressive dimensions: first, the technical evolution from accurate detection to ultra-trace quantification; second, the distribution patterns and key flavor contributions of SACs across major food categories; and third, the biosynthetic and chemical formation mechanisms governing SAC generation via the Maillard reaction, thiamine degradation, and enzymatic/microbial pathways. Together, these aspects establish the chemical foundation for targeted flavor modulation in real food systems.

### 2.1. Detection Technologies for SACs

**Figure 1:**
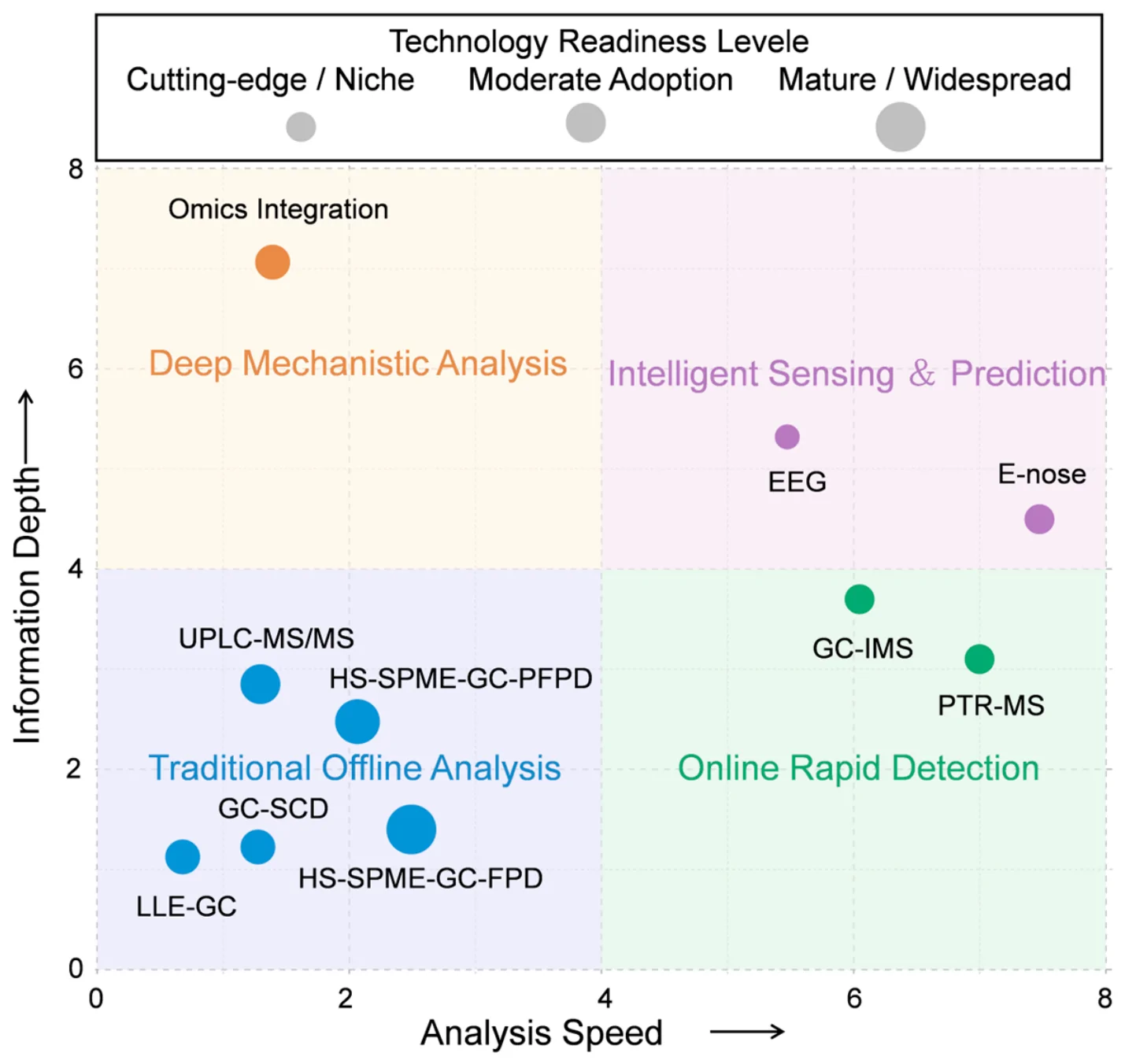
Differences in various technologies in terms of information depth, analysis speed and technical maturity for the detection of SACs.

Current analytical approaches for SACs have evolved into a multi-tier technical system centered on conventional chromatographic analysis and complemented by emerging rapid detection and intelligent sensing technologies, providing essential support for food flavor profiling and key odorant identification [25,26]. The differences among these techniques in terms of information depth, analysis speed, and technical maturity are illustrated in Figure 1.

Among traditional detection methods, gas chromatography (GC) remains the core platform for the analysis of volatile SACs. When combined with appropriate pretreatment and enrichment techniques—such as liquid–liquid extraction (LLE), headspace solid-phase microextraction (HS-SPME), dynamic headspace sampling (DHS), and stir bar sorptive extraction (SBSE)—the enrichment efficiency and detection sensitivity for target compounds can be significantly improved [27]. Among these, HS-SPME is widely applied in the separation and enrichment of SACs due to its simple operation, solvent-free nature, and suitability for complex matrices. Meanwhile, sulfur-specific detectors, such as the flame photometric detector (FPD), pulsed flame photometric detector (PFPD), and sulfur chemiluminescence detector (SCD), can further enhance the selective detection capability for SACs. In particular, SCD demonstrates strong advantages in the analysis of trace SACs owing to its high selectivity for sulfur and low detection limit [28].

For thiol compounds, derivatization strategies are particularly important due to their susceptibility to oxidation, poor stability, and insufficient detection sensitivity by direct analysis [29]. Converting thiols into more stable and more responsive derivatives can effectively improve their chromatographic behavior and enhance detection performance. Common derivatization reagents include DTDP (2,2′-dithiodipyridine), PFBBr (pentafluorobenzyl bromide), pHMB (p-hydroxymercuribenzoic acid), and ETP (N-ethylthiophthalimide). Among them, DTDP is frequently coupled with ultra-performance liquid chromatography–tandem mass spectrometry (UPLC-MS/MS) for the highly sensitive quantification of thiols in acidic food matrices, serving as one of the important methods for detecting such compounds [30].

Beyond traditional techniques, emerging detection technologies are also demonstrating promising application prospects in SACs analysis. For instance, online rapid detection techniques such as proton transfer reaction mass spectrometry (PTR-MS) and gas chromatography–ion mobility spectrometry (GC-IMS) can be used for real-time monitoring of volatile components and flavor pattern recognition during processing and fermentation [31,32,33], while intelligent sensing technologies such as electronic noses (E-nose) can be applied to aroma fingerprinting and quality discrimination [34,35]. By recording the electrophysiological signal changes in the human brain under different SACs, electroencephalography (EEG) enables objective quantification of consumers’ perceptual preferences and emotional responses to different flavor characteristics, compensating for the strong subjectivity and poor reproducibility of traditional sensory evaluation [36,37]. Furthermore, integrated omics approaches, particularly the combination of sensomics and metabolomics, allow the elucidation of key SACs and their formation mechanisms from multiple dimensions: chemical composition, sensory response, and metabolic origin [38,39].

Overall, the detection technologies for SACs are advancing from single-target analysis toward highly sensitive, high-throughput, and multidimensional integrated analysis. In the future, with the further integration of chromatographic separation, mass spectrometric detection, intelligent sensing, and data mining technologies, the capabilities for accurate identification, real-time monitoring, and mechanistic elucidation of SACs will be continuously enhanced.

### 2.2. Quantitative Methods for Key SACs

Accurate quantification is essential for evaluating the odor contribution of SACs and for constructing reliable key odorant maps. Because SACs generally occur at trace or ultra-trace levels and are highly volatile, reactive, and matrix-sensitive, their concentrations can be easily underestimated during extraction, enrichment, or instrumental analysis [40,41]. Such quantitative bias directly affects the calculation of odor activity values (OAV, the ratio of the concentration of a target flavor compound to its odor threshold) and may lead to misclassification of key food odorants [42].

Current quantitative strategies for SACs mainly include stable isotope dilution assay (SIDA), internal standard method (ISM), external standard method (ESM), and area normalization method (ANM). Among them, SIDA is generally regarded as the most reliable approach for aroma quantification. Furthermore, the matrix effect on odor thresholds is a critical factor that must be taken into account, and the odor threshold values of target compounds should ideally be determined in real sample matrices [43,44]. This advantage is particularly important for SACs, which are prone to volatilization, oxidation, adsorption, and chemical transformation in complex food matrices. Given the importance of SIDA in food flavor analysis, this review lists the preparation methods of stable isotope-labeled internal standards for some important SACs in the Supporting Information (SI).

Nevertheless, SIDA also has practical limitations. The synthesis or commercial availability of isotope-labeled SAC standards remains limited, especially for rare or newly identified sulfur odorants. In addition, deuterated analogues may show slightly different chromatographic behavior from the target compounds because of isotope effects, potentially affecting quantification accuracy [45,46]. Therefore, although SIDA provides the most robust basis for OAV calculation and key odorant screening, cross-study comparisons should consider differences in extraction methods, calibration strategies, isotope standards, and threshold media [47]. Future quantitative studies should develop more accessible isotope-labeled standards, pseudo-SIDA strategies based on high-resolution mass spectrometry, and standardized workflows to improve the comparability of SACs datasets across food systems [48].

In this review, the reported quantitative data and OAV derived from the literature serve as the methodological basis for the subsequent mapping of key SACs across food systems.

### 2.3. Key Odorant Mapping of SACs Across Food Systems

SACs are widely distributed across various food systems, and their unique aroma characteristics and extremely low perception thresholds play an indispensable role in shaping the characteristic flavor profiles of foods [49].

#### 2.3.1. Food-Category Distribution and Sensory Relevance of SACs

SACs are widely distributed, but their formation pathways and sensory roles show clear food-category specificity. In fruits and vegetables, sulfur compounds such as diethyl disulfide in durian, sulfides in Allium plants, and isothiocyanates in Brassica vegetables contribute tropical, alliaceous, pungent, or roasted notes [50,51,52]. In thermally processed foods, especially meat products, Maillard reaction- and lipid degradation-derived furan thiols and disulfides, such as 2-methyl-3-furanthiol, are indispensable contributors to meaty and roasted aromas [53,54]. In aquatic products and fermented foods, SACs such as dimethyl sulfide, thiazoles, and furfurylthiol shape marine, sulfurous, fermented, roasted, or sesame-like aroma profiles [55,56,57]. SACs are also key contributors to the roasted and nutty notes of coffee, whereas excessive or imbalanced SACs may generate undesirable off-flavors, such as grassy notes in rapeseed oil [14,58].

These examples indicate that SACs act as both flavor-defining molecules and potential sources of aroma defects. Their high potency and broad distribution make them important not only for explaining characteristic food aromas, but also for developing predictive models of flavor quality across food matrices.

#### 2.3.2. OAV-Based Mapping of Key SACs

To move beyond isolated food-specific observations, this review further constructed an OAV-based key odorant map of SACs across food systems. OAV, defined as the ratio of the concentration of an odorant in a food matrix to its odor threshold in an appropriate medium, is commonly used to estimate the contribution of individual odorants to overall aroma [59]. Compounds with OAV ≥ 1 are generally considered key food odorants in the corresponding food product [60].

Given that SACs are typically present at extremely low levels in foods and exhibit high reactivity, ease of oxidation, and volatility, accurate quantification using SIDA is essential; and in line with the definition of key food odorants proposed by Hofmann et al. [2], the inclusion was limited exclusively to studies that utilized SIDA. SIDA uses isotopically labeled internal standards that behave identically to the target analytes throughout extraction, purification, and instrumental analysis, and is currently the only technique capable of providing accurate and robust quantitation for these labile trace compounds. Non-SIDA methods cannot adequately correct for recovery losses or matrix suppression, leading to potentially erroneous concentration data and unreliable OAV, thereby compromising the identification of key odorants. Therefore, SIDA exclusivity is a scientifically necessary prerequisite to ensure the highest level of data reliability for the systematic evaluation of sulfur-containing key odorants across diverse food matrices.

The literature collection comprised two sources. (i) The dataset focused on quality was established by Hofmann and colleagues from 1980–2013 [2]. This dataset employed the following inclusion criteria specified in advance for filtering key odorants: localization of the most intense odorants directed by bioactivity using gas chromatography olfactometry analysis; identification of key odorants by comparing chromatographic retention times, mass spectrometric data, and sensory data with those of reference compounds synthesized independently; and comprehensive quantitation of the entire set of key odorants using accurate and robust techniques such as stable isotope dilution analyses. From this dataset, 42 publications reporting key odorants that contain sulfur were retained. (ii) A systematic update from 2013 to 2026 retrieved from ScienceDirect, Pubmed and Web of Science on 1 May 2026, using the same criteria, yielded 163 records, of which 59 met the inclusion criteria for sulfur-containing compounds. In total, 101 publications covering 221 food samples were included. The detailed search strategy and screening protocol are provided in Supplementary Materials.

**Figure 2:**
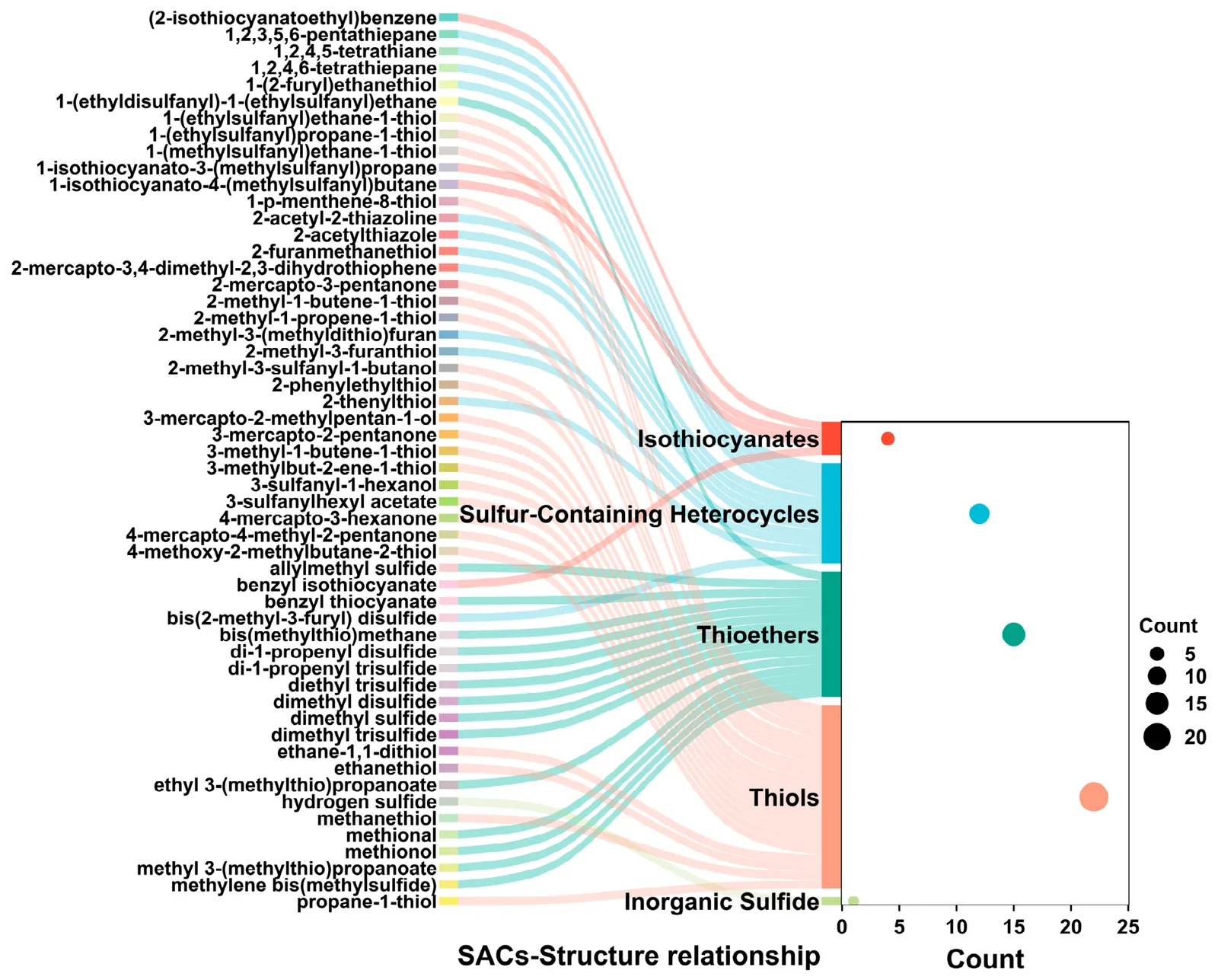
Structural classification and occurrence frequency distribution of the 54 key SACs.

Based on these data, 54 SACs with significant odor contributions were identified as key SACs. Figure 2 summarizes their structural classification and occurrence frequency, showing that thiols and thioethers are the dominant categories, followed by isothiocyanates, sulfur-containing heterocycles, and inorganic sulfides. This distribution reflects the prevalence of sulfur-containing amino acid degradation, microbial metabolism, and Maillard-type reactions across food matrices.

**Figure 3:**
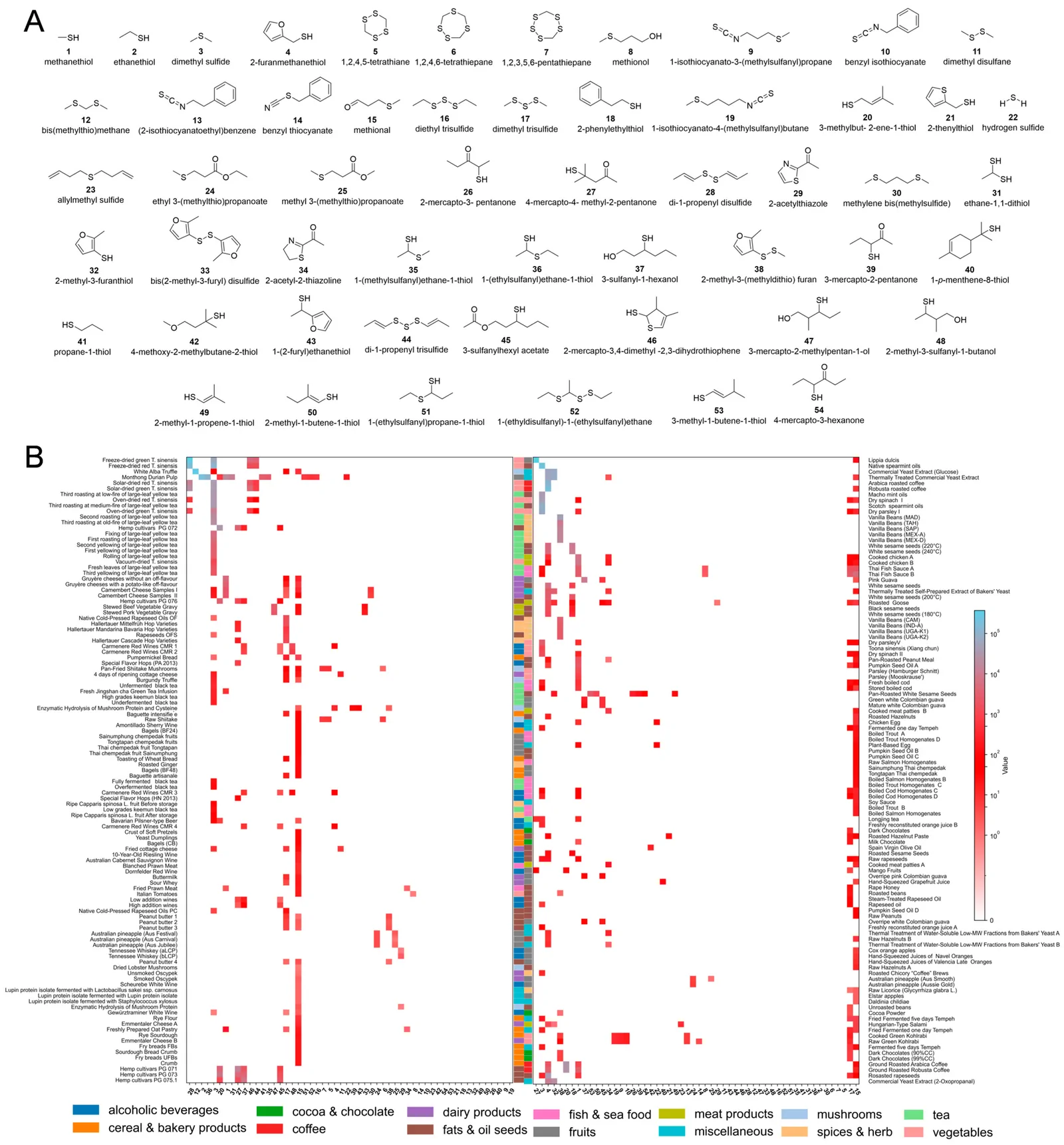
(**A**) Structures, serial numbers, and names of 54 key SACs. (**B**) Heatmap displaying the OAV and the assignment of the 54 key SACs characterized in 221 food samples.

Figure 3A shows the structures, numbers, and names of the 54 key SACs; meanwhile, the OAV heatmap in Figure 3B further reveals two major patterns of how SACs contribute to odor. First, some SACs, including methional, furfuryl mercaptan, dimethyl sulfide, methanethiol, and 2-methyl-3-furanthiol, occur frequently and exhibit high OAV across diverse matrices such as coffee [61], dairy products [62], baked foods [63], meat products [53], and fruits [64]. These compounds can be regarded as broadly distributed sulfur-containing key odorants that contribute to common sensory dimensions such as cooked, roasted, savory, and sulfurous notes [65]. Second, certain SACs show extremely high OAV but narrow food distribution. For example, ethanethiol, ethane-1,1-dithiol, and propane-1-thiol are mainly reported in durian and are closely associated with its distinctive aroma identity [50]. Therefore, SACs contribute to food aroma through both cross-matrix commonality and food-specific signature effects.

However, the interpretation of OAV-based mapping requires caution. Odor thresholds may be determined in different media, including water, oil, air, or model matrices, and may vary depending on whether GC-O, sensory threshold testing, or sensomics-based approaches are used [66]. Moreover, OAV does not fully account for retronasal release, matrix binding, mixture interactions, or nonlinear olfactory coding [67,68]. Therefore, Figure 3 should be interpreted as a reported key odorant contribution map rather than a direct quantitative comparison of odor intensity across all foods.

### 2.4. Formation Pathways Underlying SACs Distribution Patterns

The formation of SACs in foods is governed by a pathway-coupled network involving thermal reactions, thiamine degradation, and enzymatic/microbial metabolism [69,70]. These pathways explain why certain SACs repeatedly appear as key odorants across different food matrices, while others remain highly food-specific [71].

**Figure 4:**
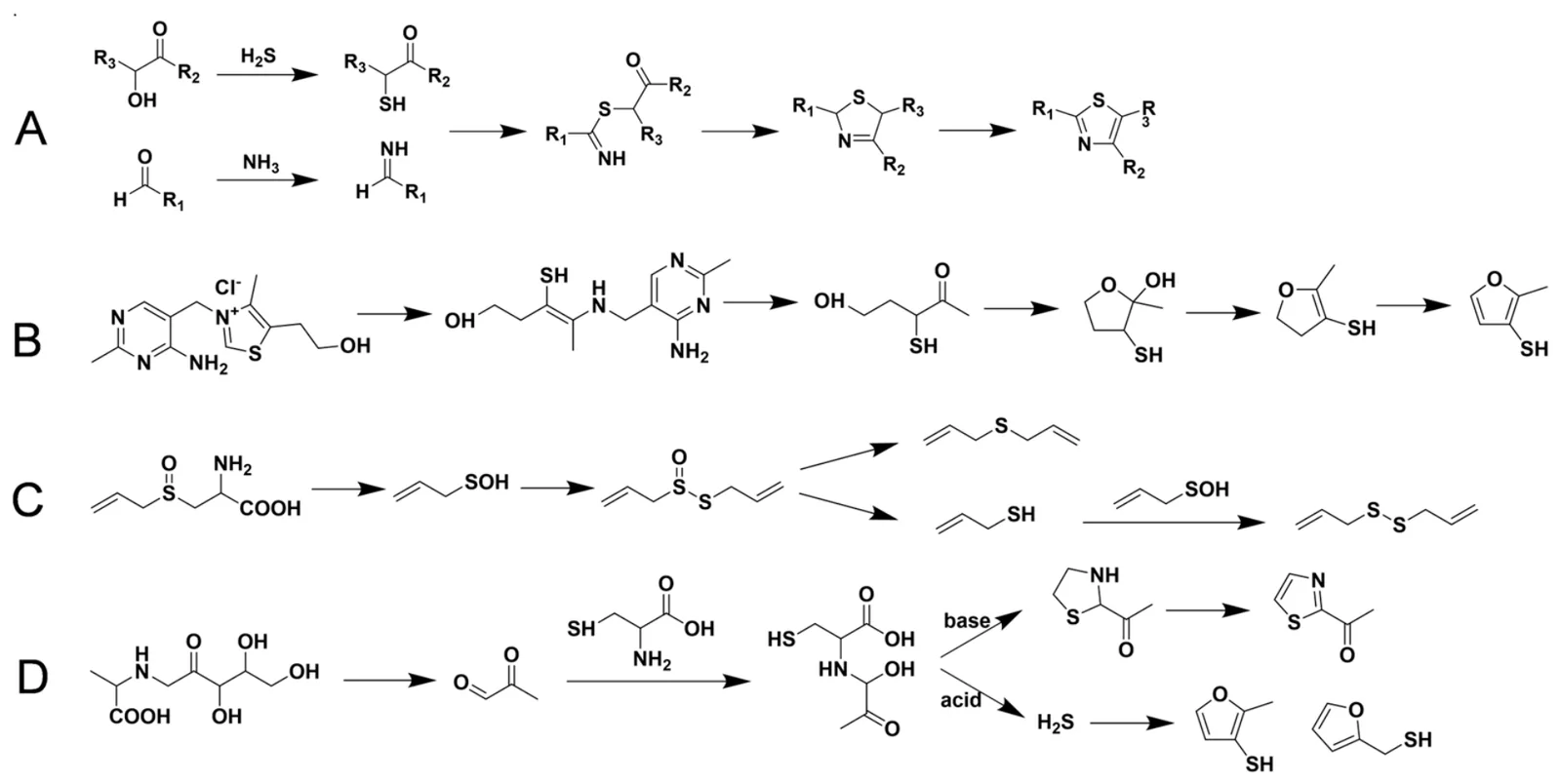
Metabolic synthesis pathways of SACs. (**A**) Maillard reaction. (**B**) Thiamine degradation. (**C**) Enzymatic reaction. (**D**) Effects of pH on the formation of SACs.

In thermally processed foods, the Maillard reaction and Strecker degradation convert sulfur-containing amino acids and reducing sugars into thiophenes, thiazoles, and thiols, thereby generating important roasted, cooked, savory, and meaty odorants (Figure 4A) [69]. Cysteine serves as a key precursor for hydrogen sulfide (H_2_S), ammonia, and acetaldehyde via pyrolysis and Strecker degradation, which subsequently react with sugar-derived carbonyl intermediates to form thiophenes, thiazoles, and thiols such as 2-furanmethanethiol [72]. Methionine undergoes Strecker degradation to produce methional, a potent cooked-savory odorant that can be further converted to methanethiol and dimethyl sulfide [73]. The sulfur intermediates generated from both amino acids have low odor thresholds and are indispensable for meaty and roasted aromas. Representative products include furfuryl mercaptan, a key contributor to the roasty aroma of coffee, and methional, which is widely associated with cooked and savory notes in heated or fermented foods [14,74]. Thiamine degradation provides another route to potent meaty odorants, such as 2-methyl-3-furanthiol and bis(2-methyl-3-furyl) disulfide, particularly in meat systems (Figure 4B) [75].

In fresh and fermented foods, enzymatic or microbial reactions dominate SACs formation [76,77,78]. Typical examples include alliinase-mediated conversion of S-alk(en)yl cysteine sulfoxides in Allium plants and microbial C-S lyase-mediated conversion of methionine to methanethiol in fermented dairy products (Figure 4C). These reactions generate matrix-specific SAC profiles and explain the characteristic alliaceous, fermented, sulfurous, or cheese-like aroma attributes of corresponding foods [70].

Processing conditions further redirect SACs formation [79,80]. pH affects the balance between H_2_S generation, thiol accumulation, and sulfur-containing heterocycle formation, while temperature influences Maillard reaction progression, lipid–Maillard interactions, and thiol oxidation (Figure 4D) [19]. Thus, precursor composition and processing control determine whether SACs contribute desirable characteristic aromas or undesirable off-flavors [71].

Overall, formation pathways provide the chemical basis for the SACs distribution patterns described above and establish a mechanistic bridge from key odorant mapping to subsequent structure–odor relationship analysis.

## 3. Advances and Limitations in Deciphering the Structure–Odor Relationship of SACs

SACs are important components that determine aroma characteristics in foods, spices, and related flavor systems. Structure–odor relationship (SOR) studies aim to elucidate the association between molecular structural features (e.g., functional group types, carbon chain length, substitution patterns, and spatial configuration) and sensory attributes (e.g., odor quality, intensity, and olfactory threshold), thereby establishing predictive relationships between molecular structure and sensory outcomes [8,81]. This constitutes the cornerstone for a deeper understanding of olfactory perception mechanisms and the rational design of novel flavor molecules. Centered on this direction, SOR research on SACs has completed the leap from “macro-level rule summarization” to “quantitative analysis at the molecular level”, and is progressively extending toward olfactory receptor recognition and binding mechanisms at the atomic level.

### 3.1. Research Approaches for SOR of SACs

**Figure 5:**
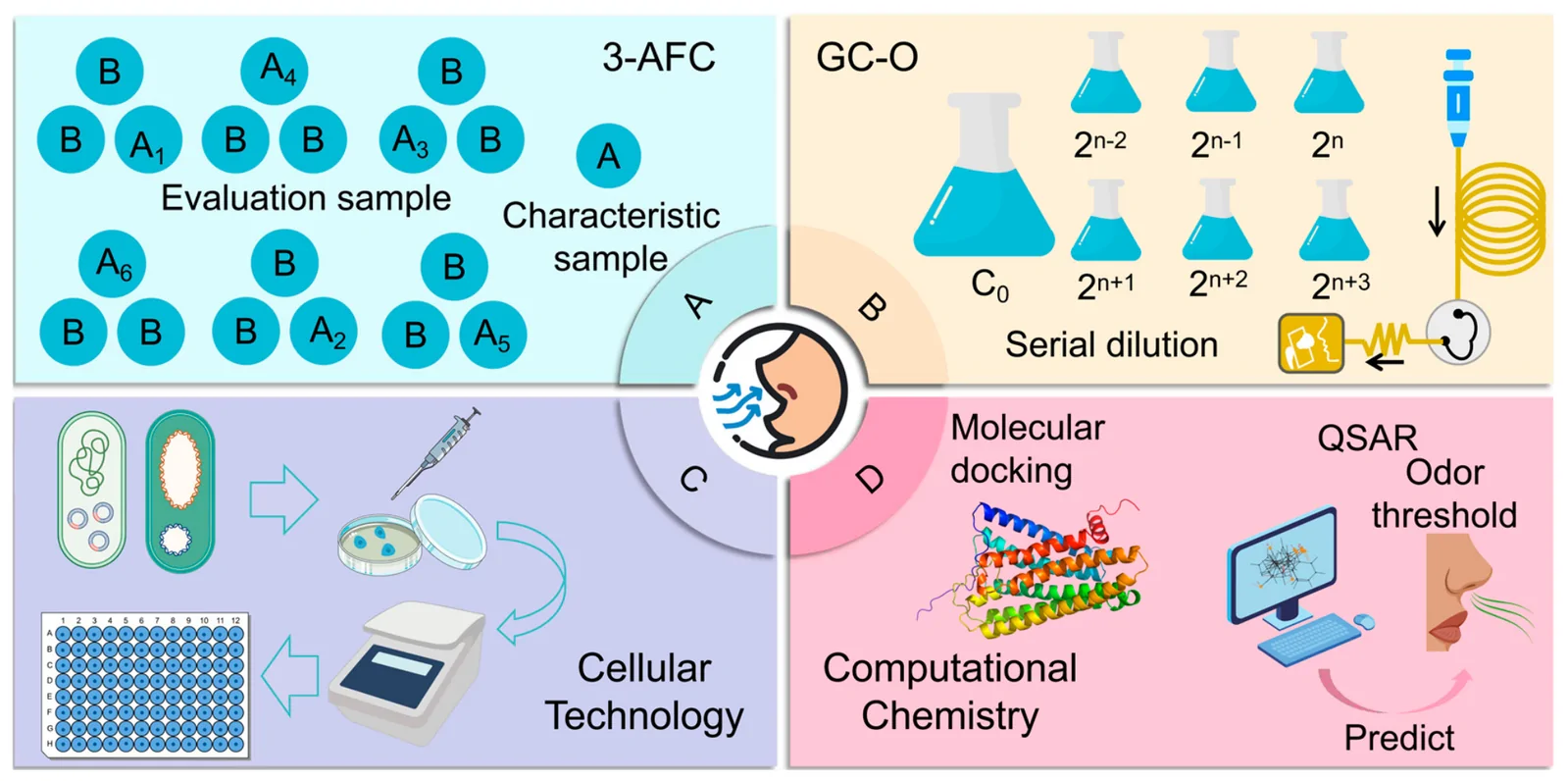
Four research methods for SACs. (**A**) Three-Alternative Forced-Choice Method (3-AFC). (**B**) Gas Chromatography-Olfactometry (GC-O). (**C**) Olfactory receptor cell-based assay. (**D**) Molecular docking and molecular dynamics simulation, and Quantitative Structure–Activity Relationship (QSAR).

Current research approaches for the SOR of SACs have formed a multi-level complementary system consisting of sensory evaluation, chromatographic analysis, receptor-level characterization, and computational simulation, primarily encompassing the following categories of methods (Figure 5).

#### 3.1.1. Three-Alternative Forced-Choice Method (3-AFC)

The 3-AFC method is a commonly used standard method for determining the olfactory thresholds of SACs (Figure 5A). Based on the principle of statistical discrimination, this method presents assessors with two identical samples and one different sample, requiring them to identify the odd one. The group olfactory threshold is then estimated by combining gradient dilution with probit analysis [82].

This method serves as an important basis for calculating OAV and can be used to systematically analyze the influence of molecular structural changes on odor intensity. Its advantage lies in its ability to measure thresholds across different food matrix systems. Its main drawbacks are the high purity requirements for standards, particularly with regard to odor purity, and a relatively cumbersome and time-consuming experimental procedure. The preparation, stability control, and storage of SACs at low concentrations all present considerable difficulty [83].

#### 3.1.2. Gas Chromatography-Olfactometry (GC-O)

GC-O combines the high separation capacity of gas chromatography with the high sensitivity of human olfactory perception, serving as the core technique for identifying key SACs in complex samples (Figure 5B). After chromatographic separation, the effluent is split proportionally between a chemical detector and an olfactory detection port, where trained assessors simultaneously record the odor characteristics, intensity, and retention time of each peak, thereby establishing a correspondence between chemical signals and sensory responses. Common data acquisition methods include the detection frequency method, aroma extract dilution analysis (AEDA), and direct intensity method [84].

In AEDA, the aroma extract is stepwise diluted at a fixed ratio, and the highest dilution at which a compound can still be detected is recorded as its flavor dilution (FD) factor, with a higher FD factor indicating stronger aroma activity. By combining AEDA with quantitative measurements (e.g., using SIDA or external calibration), FD factors can be related to concentrations, allowing the estimation of matrix-corrected odor thresholds and effectively overcoming the challenges of preparing pure standards and interference from matrix effects [8,9,10,11,12]. GC-O enables the identification of SACs at the ng/L level. By combining quantitative results with matrix-corrected thresholds, the OAV can be calculated, and the flavor contribution of individual compounds can be further verified through aroma recombination and omission experiments [85,86]. Its limitations include high instrumental cost, stringent requirements for assessors, and the potential degradation or transformation of thermally labile SACs during the chromatographic process [83].

#### 3.1.3. Olfactory Receptor Heterologous Expression Cell Technology

Olfactory receptor heterologous expression cell technology enables the direct investigation of interactions between SACs and specific olfactory receptors (ORs) by expressing these receptors in vitro, thereby revealing olfactory recognition mechanisms at the molecular level (Figure 5C). This technique is based on the signal transduction principles of G protein-coupled receptors (GPCRs) and typically evaluates the activation capacity of compounds by monitoring changes in intracellular second messengers (e.g., cyclic adenosine monophosphate (cAMP) or Ca^2+^) levels, with further calculation of the half-maximal effective concentration (EC_50_) [87].

This technology facilitates high-throughput screening and helps explain individual olfactory differences, but its limitations include the complexity of constructing the experimental system and the fact that in vitro receptor responses may not fully reflect the actual in vivo perceptual process [88,89].

#### 3.1.4. Computational Chemistry Methods

Computational chemistry methods are grounded in the principles of quantum chemistry and molecular simulation (Figure 5D). By calculating molecular electronic structures, physicochemical properties, and intermolecular interactions, these approaches predict the sensory attributes of compounds and elucidate their underlying mechanisms of action. Commonly employed strategies include QSAR, molecular docking, and molecular dynamics simulations [8,10,90].

Such methods can rapidly predict olfactory-related properties of unknown SACs and theoretically reveal possible interaction modes between molecules and receptors—such as hydrogen bonding, hydrophobic interactions, and metal coordination—thereby providing guidance for the rational design of novel flavor molecules [91]. However, their predictive accuracy is highly dependent on model construction, parameter selection, and the quality of training data, and the actual interaction processes in complex biological systems still require experimental validation [92].

### 3.2. Structural Motifs Governing the Odor Quality of SACs

**Figure 6:**
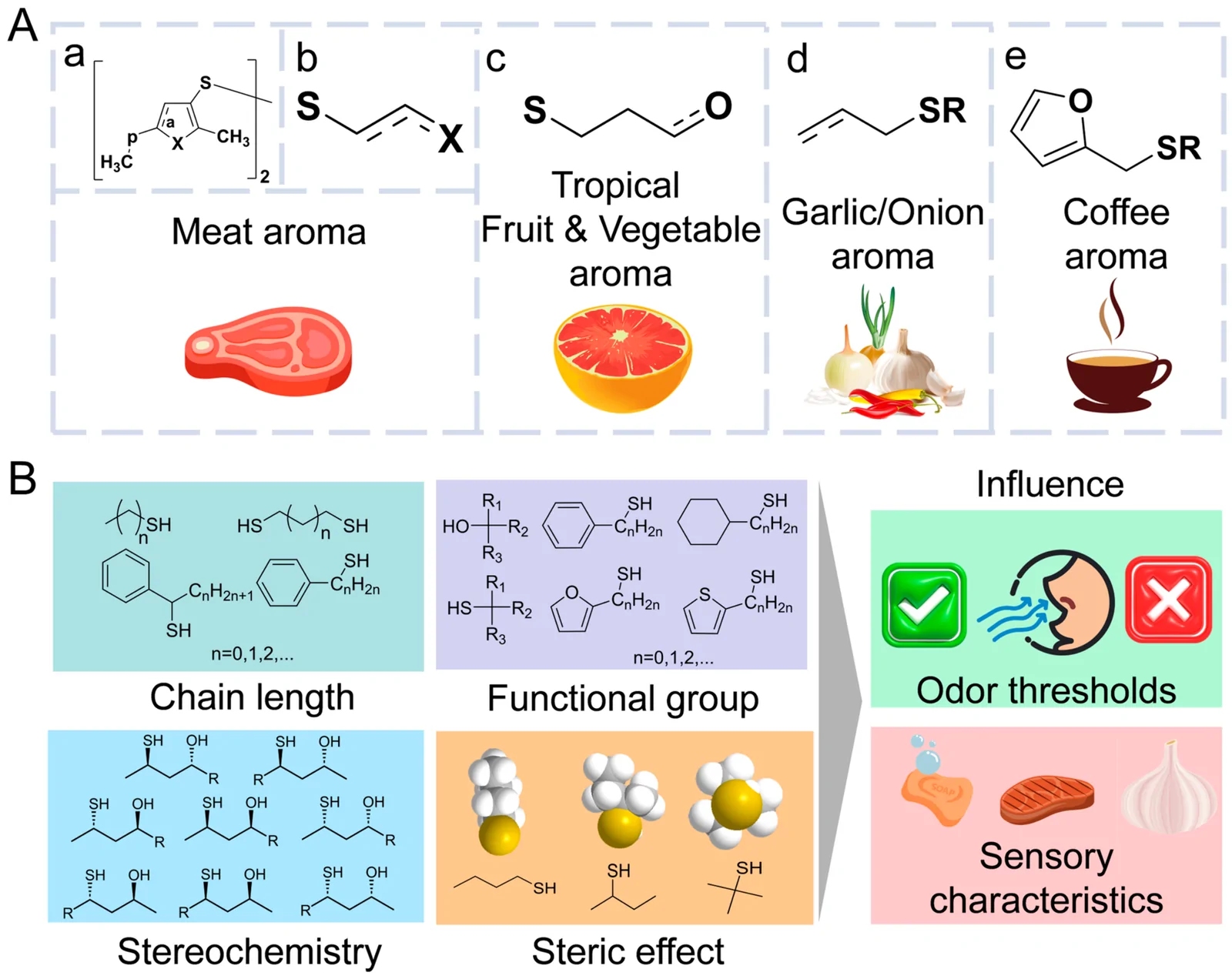
SOR of key SACs. (**A**) Characteristic structures from: (**a**) meat (Type 1); (**b**) meat (Type 2). X is O or S; p is a group coplanar with the α-carbon atom and the methyl carbon atom (such as C=O), or an atom different from O (such as S); (**c**) tropical fruits & vegetables; (**d**) garlic/onion; (**e**) coffee. (**B**) Odor thresholds and sensory characteristics governed by chain length, functional groups, stereochemistry and steric effects. Adapted from [8,12,95].

While a complete theoretical model for smell remains elusive, decades of research have successfully associated specific molecular frameworks with distinct aroma qualities, providing an invaluable, if incomplete, empirical guide (Table S2). For meaty aromas, foundational work identified key structural prerequisites. Early models emphasized geometric features like a free-rotating methyl group and specific distances between heteroatoms in furan- or thiophene-based sulfides (Figure 6(Aa)) [93]. This was later synthesized into the recognition of a 1,2-oxygen/sulfur or 1,2-sulfur/sulfur skeleton as a core “meaty olfactophore”, explaining the sensory properties of seminal compounds like 2-methyl-3-furanthiol and bis(2-methyl-3-furyl) disulfide (Figure 6(Ab)) [7]. Tropical and fruity notes, in contrast, are strongly linked to polyfunctional thiols featuring a 1,3-oxygen-sulfur arrangement. This “tropical olfactophore” is exemplified by compounds such as 4-mercapto-2-heptanone and its corresponding alcohol, where the specific spatial arrangement of sulfur and oxygen is critical (Figure 6(Ac)) [94,95]. Similarly, the pungent alliaceous character of garlic and onion is reliably associated with allylthio or propylthio groups, with subtle structural differences dictating the specific garlic- or onion-like nuance (Figure 6(Ad)) [96]. Finally, the roasted coffee character is dominantly imparted by the furfurylthio moiety, as seen in furfurylthiol and its derivatives (Figure 6(Ae)) [96].

### 3.3. Molecular Parameters Determining the Odor Thresholds of SACs in Air

Beyond identifying broad structural motifs, detailed studies [8,9,10,11,12,94,95,97,98,99] have quantified how finer parameters, including functional groups, chain length, stereochemistry, and steric environment, precisely tune odor activity (Figure 6B). Notably, the odor thresholds and sensory characteristics of these SACs were both measured by GC-O in the airborne phase.

The thiol (-SH) group is paramount, often conferring odor intensities orders of magnitude greater than its alcohol (-OH) analogue, fundamentally altering odor character [97]. Systematic work by Schieberle’s team revealed nuanced dependencies: in homologous series of aliphatic thiols and mercaptoalkanols, odor thresholds exhibit a parabolic relationship with chain length, reaching an optimum (e.g., C6 for 1-hexanethiol at 0.010 ng/L) [8,10]. Crucially, replacing the thiol with other groups drastically reduces potency, underscoring its unique role.

Stereochemistry (i.e., the three-dimensional arrangement of atoms in a molecule) is a key determinant of odor quality and potency. For the “tropical olfactophore”, the (*S*)-configured 4-mercapto-2-alkanones possess lower thresholds and more desirable fruity notes than their (*R*)-counterparts, while for 4-mercapto-2-alkanols, the (2*R*,4*R*)-diastereomer is most potent [98,99]. This chiral recognition implies highly specific interactions with olfactory receptors. However, such clear correlations can vanish with minor structural shifts; 2-mercapto-4-alkanones lack the strong tropical character and show weaker stereochemical differentiation [94,95].

Steric accessibility (i.e., the spatial availability of the thiol group for receptor binding) is equally critical. Studies on aromatic and cyclic thiols demonstrate that shielding the thiol group by bulky substituents or incorporating it into a constrained ring system significantly increases odor thresholds, whereas exposed primary thiols maintain ultra-low detection levels [8,9,10,11,12]. This highlights the importance of the molecule’s three-dimensional shape for receptor binding.

Further exploration of SOR reveals that heteroatom substitution within the ring skeleton is not a simple superposition of functional groups. For example, replacing the oxygen atom in damascenolide with sulfur yields the 2,5-dihydrothiophen-2-one analogue, which does not produce the expected sulfurous odor but instead exhibits waxy, fatty, and lactone-like characteristics, indicating that the odor of SACs is an emergent property of the molecule’s entire electronic system [100]. At the other end of the molecular-size spectrum, high-molecular-weight thioesters (>260 Da) synthesized from 4-ethyloctanoyl chloride, despite their low volatility, can continuously release distinct meaty, roasted, and sulfurous notes over an extended period, demonstrating that increasing molecular weight can shift functionality from immediate perception toward aroma persistence, which is crucial for flavor retention in complex food matrices [101].

These findings collectively illustrate a sophisticated, multi-parameter SOR where odor is an emergent property of the entire molecular structure. While quantitative trends within homologous series are well-established, a critical predictive gap persists: the current empirical and QSAR models struggle to extrapolate accurately across different SAC classes (e.g., from thiols to sulfides) or to account for the non-linear, combinatorial nature of odor perception in mixtures [102]. Furthermore, these structure–activity correlations, often derived in isolation, frequently fail to predict odor in real food contexts, highlighting the need to move beyond static molecular structures toward a systems-level perspective that integrates receptor biology, perceptual neurobiology, and food matrix physics [103]. This gap will be further elaborated in the context of computational and matrix-related limitations in Section 3.4 and Section 5.

### 3.4. Olfactory Receptor Recognition of SACs

**Figure 7:**
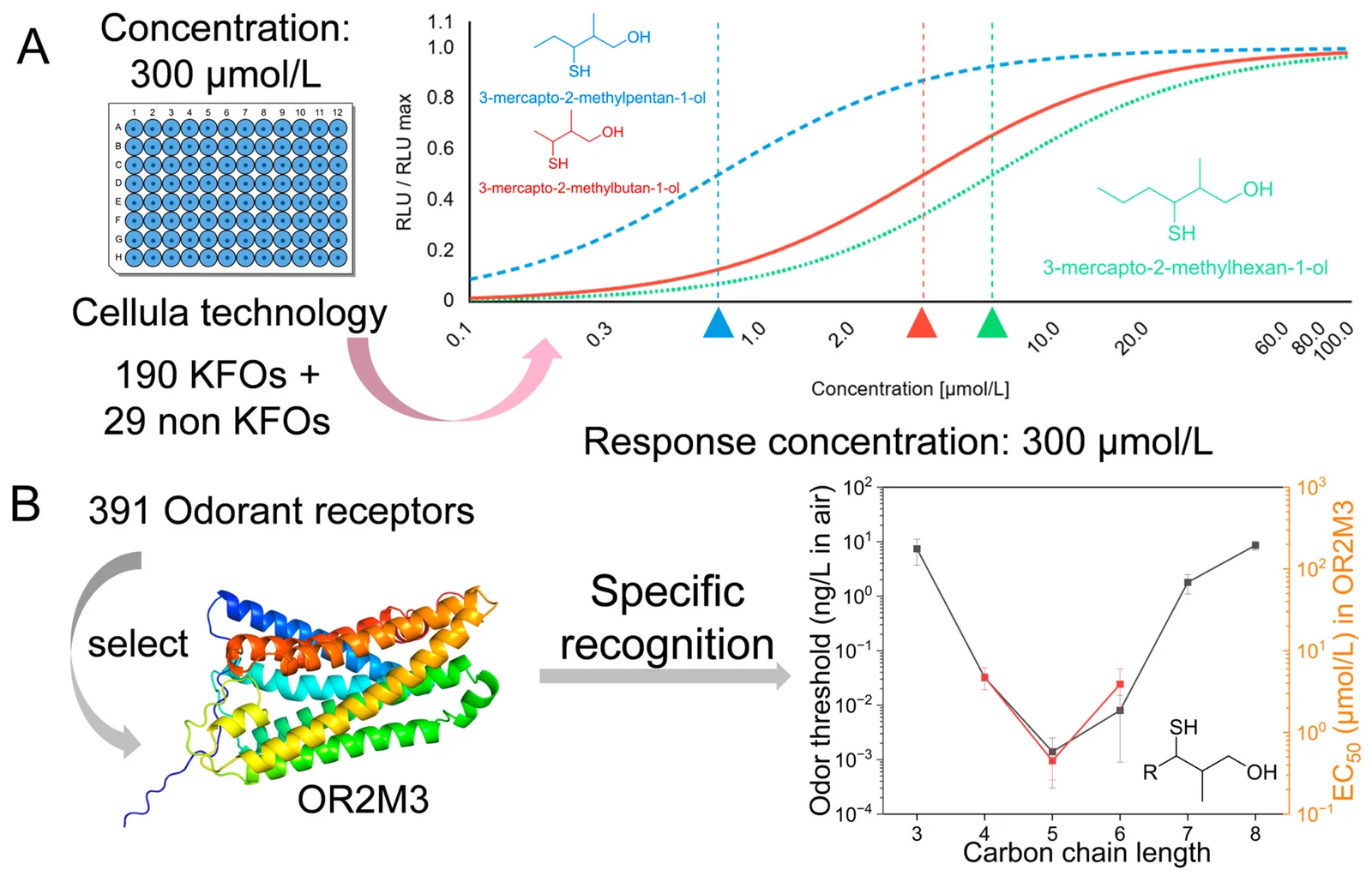
(**A**) 3-mercapto-2-methylpentan-1-ol is the core optimal ligand of OR2M3 with an EC_50_ lower than 1 μmol/L. (**B**) Comparative analysis of in vivo odor thresholds and in vitro EC_50_ values for the homologous series of 3-mercapto-2-methylalkan-1-ols activating OR2M3 receptor. Adapted with permission from Ref. [104]. Copyright 2016 Oxford University Press.

The study of SOR in SACs has identified ORs as crucial determinants of their high potency and specificity. KFOs among SACs, particularly thiols with extremely low odor thresholds, are often detected by highly specialized ORs. For instance, the human receptor OR2M3 responds exclusively to the onion KFOs 3-mercapto-2-methylpentan-1-ol (Figure 7A). Strikingly, its in vitro EC_50_ values for a homologous series mirror the in vivo odor threshold trend: both exhibit a pronounced V-shape across C4 to C6 chain lengths, with the highest potency (lowest EC_50_ and threshold) at the C5 homologue. This high selectivity is underscored by its complete lack of response to analogues with chains shorter than C4 or longer than C6, revealing its narrowly tuned nature (Figure 7B) [104]. This function is not found in its archaic hominin orthologs, suggesting a modern human-specific dietary adaptation. In contrast, broadly tuned receptors like OR2W1 recognize a wide spectrum of structurally diverse KFOs, including various SACs, highlighting its role in combinatorial odor coding [87]. This dual strategy of “specialist” and “generalist” receptors enables a multi-layered perceptual encoding. Moreover, single nucleotide polymorphisms (SNPs) in OR genes can alter ligand sensitivity, underpinning individual differences in sulfurous odor perception.

A major breakthrough in understanding the structural basis of how ORs recognize ligands came with the first experimentally determined cryogenic electron microscopy structure of a human odorant receptor, OR51E2, bound to propionate, a fatty acid with a short chain (PDB ID: 8F76) [105]. This structure revealed that propionate binds within a fully occluded pocket enclosed by transmembrane helices 3–6 (TM3-TM6), forming specific ionic and hydrogen bonds with the carboxylic acid moiety (notably via R262^6×59^) and van der Waals interactions with the aliphatic chain. Crucially, mutagenesis and computational modeling demonstrated that the geometric volume of this occluded pocket acts as a selectivity filter: expanding the pocket by mutating F155^4×57^ or L158^4×60^ to alanine enabled the receptor to accommodate and respond to fatty acids with longer chains (up to C8 and C6, respectively), while reducing potency for the native ligands with short chains. Although OR51E2 recognizes fatty acids rather than odorants containing sulfur, this work establishes fundamental principles directly relevant to the recognition of SACs: (i) odorant selectivity is governed by tight shape complementarity within an occluded pocket; (ii) extracellular loop 3 (ECL3) functions as a critical dynamic gate, with inward movement of TM6 and ECL3 upon ligand binding driving receptor activation; and (iii) the FYGx^6×50^ motif specific to ORs in TM6 serves as a structural pivot distinct from the CWxP motif of class A GPCRs that are not olfactory. These insights provide a critical structural framework for interpreting the narrow tuning of receptors such as OR2M3, where precise pocket geometry likely enforces strict specificity for chain length in thiols, and suggest that future cryogenic electron microscopy structures of ORs specific for SACs will reveal analogous, chemically tailored recognition logic.

A central challenge in advancing this field lies in deciphering the intricate combinatorial coding rules that govern OR-ligand interactions, where a single odorant can activate multiple receptors and a single receptor can respond to diverse ligands. Recently, two studies have provided important references from different perspectives: Bintu et al. [106] established a spatial molecular atlas of all olfactory receptors in the mouse, revealing the topological principle that natural social odors are encoded by independent spatial domains; Zhang et al. [107] developed enzyme/quantum dot composite gas-selective electrodes mimicking human olfactory receptor cells, achieving specific recognition of hydrogen sulfide, phenol, and other molecules in complex gas mixtures, offering a biomimetic strategy for artificial olfactory sensing. Future systematic receptor screening and structural modeling are poised to further elucidate these precise structure–activity relationships, providing a molecular foundation for sensory design.

### 3.5. Computational Elucidation of the SOR of SACs

The receptor screening and functional assays reviewed in Section 3.4 have unveiled recognition patterns at the receptor level, while molecular docking and molecular dynamics (MD) simulations have further elucidated the binding mechanisms between SACs and ORs at atomic resolution, bridging receptor-level screening with atomic-level mechanisms and providing a powerful theoretical framework for understanding the interactions between SACs and ORs.

Early computational studies of interactions between ORs and ligands relied heavily on homology models built from distantly related class A GPCRs, which suffered from low sequence identity and limited structural accuracy. However, the determination of the experimentally resolved structure of human OR51E2 by cryogenic electron microscopy (PDB ID: 8F76) [105] has fundamentally transformed this landscape. The OR51E2 structure confirms the canonical architecture of seven transmembrane helices but reveals features specific to ORs and not captured by previous homology models, most notably the occluded ligand-binding pocket, the FYGx^6×50^ connector motif, and the critical role of ECL3 dynamics in activation. For interactions between SACs and ORs, this breakthrough means that future docking and MD studies can now leverage experimentally validated structural templates and interaction fingerprints, such as persistent contacts involving ionic interactions or hydrogen bonds with conserved pocket residues and metrics of shape complementarity, rather than speculative models with low homology.

At the level of olfactory perception, the binding modes of SACs with ORs exhibit remarkable specificity and diversity. For instance, Xiao et al. [108] found that dimethyl sulfide in Longjing tea binds to the olfactory receptor OR1A1 primarily through hydrophobic interactions (involving residues such as Leu162 and Ile181), which explains the diffusivity and rapid perception characteristics of low-molecular-weight thioethers. Meanwhile, Haag et al. [109], in their study of the broadly tuned receptor OR2W1, revealed that sulfur-containing thiol esters (e.g., 3-mercaptohexyl acetate) preferentially form strong and persistent hydrogen bonds with the key residue Tyr259^6.51^ (MD simulations showed a hydrogen bond occupancy of up to 64.33%), and the calculated highly negative binding free energy (−22.67 ± 2.02 kcal/mol) supports the stability of this interaction. Liu et al. [90] discovered that SACs in rapeseed oil (3-butenyl isothiocyanate, dimethyl/trisulfide) primarily rely on hydrogen bonding for receptor binding, whereas the non-sulfur compound phenylacetonitrile is recognized through π-interactions. Such fundamental differences in binding modes may lead to significant distinctions in the dynamics of olfactory perception (e.g., saturation kinetics) between the two, highlighting the key role of the sulfur atom in molecular recognition.

The OR51E2 structure further validates the utility of MD simulation methodologies applied to systems involving SACs and ORs. In OR51E2, five independent MD simulations of one microsecond each quantified contact frequencies between propionate and pocket residues, tracked ECL3 conformational flexibility, and demonstrated that ligand binding stabilizes the connector region by restraining F250^6×47^ rotamers and maintaining the hydrogen bond between S111^3×40^ and Y251^6×48^. These same analytical approaches, including heatmaps of contact frequencies, monitoring of distance trajectories, and calculations of pocket volume, are directly transferable to receptors specific for SACs. For example, the observation that an occluded pocket enforces strict ligand length selectivity in OR51E2 supports the hypothesis that the narrow tuning of OR2M3 for the C5 compound 3-mercapto-2-methylpentan-1-ol arises from similarly constrained pocket architecture. Likewise, the finding that R262^6×59^ in ECL3 acts as an activation trigger by coordinating the polar headgroup of the ligand suggests that ORs specific for thiols may employ analogous conserved arginine or tyrosine residues to anchor the sulfhydryl group.

Nevertheless, it is important to note that experimentally determined structures of ORs specific for SACs, such as OR2M3, OR2W1, or OR1A1, remain to be solved. Until such structures become available, the OR51E2 framework offers the most reliable structural foundation for computational studies of the recognition of SACs. Future integration of cryogenic electron microscopy structures for ORs tuned for sulfur compounds, combined with systematic MD simulations and docking against the validated OR51E2 template, will enable precise mapping of how diverse sulfur functionalities (thiols, thioethers, isothiocyanates, and thiol esters) are geometrically and energetically accommodated within the binding pockets of distinct ORs, ultimately delivering an understanding of sulfurous odor perception at the atomic level.

Nevertheless, the limitations of computational analysis must be acknowledged: the structural accuracy of current OR homology models remains limited, and the timescale of MD simulations is still insufficient relative to the real ligand-receptor association–dissociation kinetics; a gap persists between in vitro single-receptor responses and the perceptual outcomes of multi-receptor combinatorial coding in vivo. Furthermore, current molecular force fields often lack the necessary precision for describing interactions involving sulfur atoms, and most simulation systems employ simplified aqueous environments that do not capture the multi-phase complexity of real foods. Future efforts should integrate cryo-electron microscopy structural determination with enhanced sampling algorithms, develop force field parameters tailored for sulfur-involved interactions, and progressively improve the physiological relevance of simulations by incorporating multi-component food matrices.

## 4. Flavor Modulation and Application of SACs in Food Matrices

With deepening research into the flavor mechanisms of SACs, their extremely low olfactory thresholds and distinctive meaty, roasted, and burnt aroma characteristics have demonstrated multidimensional application potential in plant-based meat products, flavor bases, and salt-reduced foods. However, the efficacy of SACs highly depends on their interaction modes with food matrices, dosage, and processing conditions [24,110]. In real food systems, SACs application can be regarded as a matrix-mediated flavor design process: molecular binding first regulates SACs retention, volatility, and release; release behavior then determines olfactory accessibility and sensory perception; and these perceptual effects can be exploited for aroma enhancement, off-flavor masking, and cross-modal taste modulation [111]. Nevertheless, successful translation from simplified model systems to real foods requires consideration of multiphase structures, competitive interactions, dosage effects, and processing conditions [112]. Therefore, this section evaluates SAC applications from the perspectives of molecular binding and release mechanisms, sensory modulation, system stabilization, functional applications, and the remaining gap between model systems and real food matrices.

### 4.1. Interactions of SACs with Food Proteins and Flavor Retention Mechanisms

**Figure 8:**
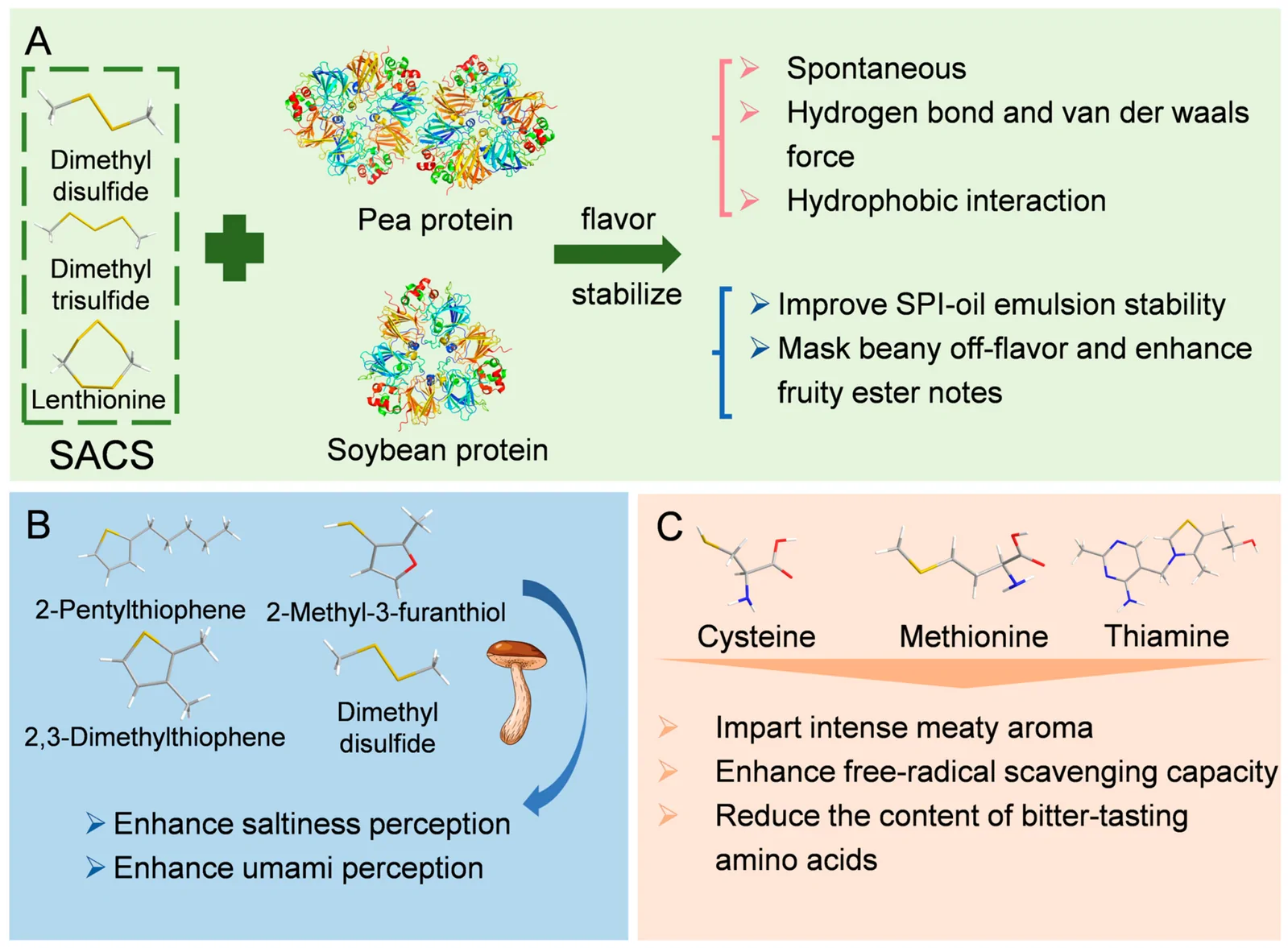
Flavor regulation and applications of SACs in food matrices. (**A**) Flavor retention mechanism between SACs and pea protein, as well as their effects on stability improvement and flavor modification of soy protein emulsion systems. Adapted from [110,115]. (**B**) Enhancement of saltiness perception by SACs. (**C**) Selective regulatory effects of sulfur-containing precursors on the generation of Maillard reaction-derived flavors.

Beyond their application in elucidating the binding mechanisms between SACs and olfactory receptor proteins, computational methods have also shed light, at the level of food matrix–flavor regulation, on how SACs influence their volatility and sensory performance through interactions with food components, particularly proteins (Figure 8A). Studies by Zhu et al. [113] and Wei et al. [114] demonstrated that both kafirin and beef myofibrillar proteins can bind to sulfur-containing or heterocyclic flavor compounds through hydrogen bonding and hydrophobic interactions. This encapsulation effect suppresses their volatilization, thereby increasing their odor threshold or altering their release profiles.

Systematic investigations by Sun et al. [110,115] further revealed how differences in the molecular structure of SACs precisely modulate their binding behavior with plant proteins (e.g., pea protein, soy protein isolate): linear thioethers (such as dimethyl disulfide and dimethyl trisulfide) primarily rely on hydrogen bonding, whereas cyclic polysulfides (such as lenthionine), by virtue of their larger hydrophobic surface area, higher hydrophobicity constants, and electronic systems, exhibit stronger binding affinity through hydrophobic interactions, π-sulfur bonds, and van der Waals forces. This structure–affinity relationship has clear sensory consequences: linear thioethers, which bind more weakly to proteins, are more readily released, contributing to enhanced meaty aroma, whereas cyclic compounds with high affinity can effectively mask beany off-flavors through encapsulation. Moreover, although such non-covalent interactions facilitate flavor retention, they can induce a transition in protein secondary structure from α-helix to β-sheet and lead to protein aggregation and increased particle size [110,115].

Notably, in addition to non-covalent binding, SACs (particularly thiols and disulfides) can also undergo disulfide bond exchange or covalent adduction with cysteine-rich proteins such as β-lactoglobulin. While this enhances binding stability, it may also cause irreversible loss of flavor compounds, thereby affecting product shelf life.

A limitation of computational studies is that current molecular force fields still require improved accuracy in describing the interactions between sulfur atoms and protein residues, and most simulation systems involve simplified aqueous environments, which differ from the multi-phase complexity of real foods [116,117]. Future efforts should develop force field parameters specifically tailored for sulfur-involved interactions and construct multi-component simulation systems incorporating lipids and polysaccharides.

### 4.2. Flavor Construction and Off-Flavor Masking

SACs exhibit multiple mechanisms in the flavor regulation of plant-based foods, with their roles extending from traditional off-flavor masking and meaty aroma construction to the direct modulation of basic taste perception (Figure 8B). Recent studies [110,118,119] have shown that in a high-moisture extrusion system for textured soybean protein, the D-xylose-cysteine precursor system generates 2-methyl-3-furanthiol and furfuryl mercaptan in situ, significantly masking the hexanal-derived beany off-flavor. Molecular dynamics simulations further reveal that these thiols can competitively bind to the olfactory receptor OR51E2 through hydrogen bonding and π-π stacking, thereby blocking off-flavor signal transduction [118]. Similarly, in pea protein and sesame meal systems, electronic nose and sensory analyses have confirmed that the addition of SACs amplifies meaty sulfur notes while suppressing the release of alcohol and aldehyde volatiles associated with beany or burnt flavors [110,119].

Building on this, the application of SACs has also demonstrated capabilities in cross-modal perceptual enhancement and receptor-level modulation. In salt reduction strategies, volatile thiophenes (e.g., 2,3-dimethylthiophene, 2-pentylthiophene) and thiols (e.g., 2-methyl-3-furanthiol) can generate cross-modal synergy with NaCl through the OISE effect, significantly boosting perceived saltiness intensity in low-sodium systems [23,24]. A distinct, receptor-level mechanism has also been reported: certain SACs (e.g., methanethiol, dimethyl sulfide, dimethyl trisulfide) can act directly as positive allosteric modulators of the taste receptor type 1 member 1/member 3 (T1R1/T1R3) umami receptor. By reshaping the receptor binding pocket and strengthening the hydrogen bond network between monosodium glutamate (MSG) and the receptor, they enhance umami perception independently of olfactory cross-modal interactions [22]. This direct regulatory mechanism at the receptor level provides a theoretical basis for developing novel umami enhancers; however, attention must be paid to its dose effect—excessively high concentrations of SACs may produce unpleasant sulfurous or putrid odors that disrupt the overall flavor balance.

### 4.3. System Stability and Functional Applications

SACs exhibit a dual functionality as both flavor modulators and physical stabilizers in functional food systems (Figure 8A), while different sulfur-containing precursors exert markedly selective regulatory effects on the targeted generation of Maillard reaction flavors (Figure 8C).

Regarding colloidal system stability, hydrophobic SACs (e.g., lenthionine) can embed themselves into the oil–water interface stabilized by soy protein through hydrophobic interactions, enhancing the compactness of the interfacial protein film and thereby improving the emulsifying activity index (EAI) and emulsion stability index (ESI); concurrently, they can suppress the release of undesirable volatiles such as hexanal and 1-octen-3-one, while promoting the formation of pleasant esters like ethyl acetate [115].

With respect to the differential regulation of Maillard reaction flavors by sulfur-containing precursors, different precursors direct the formation of characteristic flavor compounds through selective reaction pathways. In *Pleurotus citrinopileatus* hydrolysate systems, L-cysteine (Cys) tends to promote the generation of sulfur-containing heterocyclic compounds (e.g., thiophenes, thiols), imparting strong meaty, kokumi, and umami notes to Maillard reaction products (MRPs), while significantly enhancing 2,2-diphenyl-1-picrylhydrazyl (DPPH) radical scavenging capacity, 2,2′-azino-bis(3-ethylbenzothiazoline-6-sulfonic acid) (ABTS) radical scavenging activity, and reducing power; in contrast, L-methionine (Met) primarily drives the formation of furan compounds, endowing MRPs with caramel-like aroma characteristics [120]. Although thiamine (Thi) lowers the system pH and inhibits browning, its degradation products (e.g., 4-methyl-5-thiazoleethanol) also make important contributions to meaty aroma. This study further demonstrated that the introduction of sulfur-containing precursors not only altered the molecular weight distribution and free amino acid composition of MRPs (e.g., reducing bitter amino acid content), but Partial Least Squares Regression (PLSR) analysis also revealed that thiols and Maillard peptides are the primary contributors to meaty and kokumi notes, while furans are highly correlated with caramel-like notes. These findings corroborate results obtained from sesame meal [119] and chicken skeleton [121] systems, namely that cysteine is the core precursor for constructing meaty-aroma MRPs, whereas methionine is more favorable for the formation of caramel-sweet aroma profiles.

However, the application of sulfur-containing precursors exhibits a clear dose effect: excessive cysteine can disrupt the fibrous protein structure during extrusion, whereas excessive methionine may lead to the accumulation of thioethers such as dimethyl disulfide, generating undesirable onion- or garlic-like off-flavors [118].

### 4.4. The Application Gap from Model Systems to Real Foods

Overall, the application of SACs in foods exhibits a pronounced structure–dose–function dependence. Their advantages lie in their multi-target, high-efficiency flavor modulation capabilities (encompassing flavor retention, off-flavor masking, cross-modal flavor enhancement, antioxidant activity enhancement, and functional stabilization), whereas the challenges include potential perturbation of protein structures, irreversible flavor loss due to covalent binding, and off-flavor risks at high concentrations. The most fundamental challenge is that most current mechanistic studies are based on model systems (such as pure protein solutions or simplified Maillard systems), whereas the multi-component, multi-phase characteristics of real foods (e.g., fat globules, starch granules, fiber networks) exert non-negligible modulating effects on the release and perception of SACs. Future research should further integrate sensomics with neuroscience techniques to elucidate the regulatory patterns of SACs on central flavor coding at the holistic level, and optimize their targeted delivery and controlled release strategies in complex food matrices, so as to achieve synergistic enhancement of both flavor quality and nutritional function.

## 5. Conclusions and Future Perspectives

Research on the SOR of SACs is in a critical transition from empirical induction to molecular mechanism elucidation. This review summarizes the detection and quantification methods, distribution characteristics, key odor contributions, and biosynthetic/metabolic pathways of SACs in foods, clarifying how molecular skeleton, stereochemistry, chain length, and steric hindrance regulate odor activity. Olfactory receptor recognition and molecular simulations have revealed the perceptual basis of their potent flavors at receptor and atomic levels. However, a predictive gap from microscopic molecular mechanisms to macroscopic sensory manifestation persists: the complexity of food matrices, dynamic transformation of SACs during processing and storage, and nonlinear olfactory combinatorial coding together constitute the major bottleneck for rational design.

To overcome these limitations, the research paradigm in this field needs to evolve toward deep data integration and mechanism-driven model construction, with breakthroughs focusing on the following three directions:

Developing a multiscale predictive framework integrating neuroscience. Constructing a cross-scale model that traverses “molecular structure–receptor activation–neural response–sensory perception” requires integrating computational chemistry and molecular dynamics simulations with psychophysics and neuroimaging techniques. For instance, EEG, as a non-invasive neurosensory analysis technique, has recently been introduced to objectively quantify consumers’ perceptual preferences and emotional responses to different SACs, thereby compensating for the subjectivity of traditional sensory evaluation [36,122]. By establishing quantitative correlations between structural parameters of SACs and specific EEG response patterns (e.g., power changes in specific frequency bands), it may be possible to bypass the complex olfactory coding step and directly establish a predictive mapping from chemical structure to neural activity, offering a new perspective for understanding the nonlinear characteristics of perception.

Advancing artificial intelligence to decode olfactory perceptual coding. Machine learning and deep learning are powerful tools for tackling the challenge of complex olfactory coding. Using graph neural networks and large-scale olfactory databases, it is possible not only to predict the sensory attributes of molecules but also to decipher their potential receptor activation profiles. Such AI-derived “odor fingerprints”—high-dimensional vectors representing the predicted activation pattern across a repertoire of olfactory receptors—can serve as an intermediate phenotype linking chemical structure to perceptual output [123]. By training models to learn the complex mapping relationships among “structure–fingerprint–perception”, AI holds the potential to reversely deduce certain rules of olfactory coding and even generate novel molecules capable of specifically activating target receptor combinations to elicit desired aroma perceptions.

Constructing a closed-loop green biomanufacturing system. A deep understanding of SOR should ultimately serve sustainable production practices. The bioconversion of natural substrates into SACs via enzymatic or microbial methods—such as the reaction of Goji berry peptides with cysteine to generate meaty flavorings [72], fungal fermentation to produce natural meaty flavors [124], and yeast metabolic engineering [125]—has become an important direction. With the rise in plant-based and cultured meat, rationally designed SACs will play a crucial role in masking off-flavors and imparting meaty aroma.

In summary, the future of SACs and SOR research lies in a transition from a descriptive discipline, which relies on empirical knowledge and isolated data, toward an integrative science that not only incorporates neural perception mechanisms but is also co-driven by theory and data, ultimately enabling green and precision manufacturing. By integrating key odorant mapping, structure–odor decoding, and matrix-based applications, this review provides a conceptual framework for transforming SACs research from descriptive flavor chemistry toward predictable and designable food aroma systems.

## Data Availability

No new data were created or analyzed in this study. Data sharing is not applicable to this article.

## Supplementary Materials

The following supporting information can be downloaded at: https://www.mdpi.com/article/10.3390/foods15173003/s1. The following files are available free of charge. Figure S1. Flow Chart for the Synthesis of [^2^H_3_]-methional; Figure S2. Flow Chart for the Synthesis of [^2^H_3_]-2-methyl-3-furanthiol or [^2^H_3_]-Bis(2-methyl-3-furyl)disulfide; Figure S3. Flow Chart for the Synthesis of R-S-S-CD_3_; Figure S4. Flow Chart for the Synthesis of [^2^H_6_]-3-mercapto-3-methylbutyl; Figure S5. Flow Chart for the Synthesis of [^2^H_3_]-4-mercapto-3-hexanone; Figure S6. Flow Chart for the Synthesis of [^2^H_4_]-2-acetyl-2-thiazoline. Synthetic methods for stable isotopes, along with Table S1 (listing 54 SACs identified as decisive KFOs across 221 food samples), Table S2 (showcasing SACs with characteristic molecular scaffolds), and their associated references. References [126,127,128,129,130,131,132,133,134,135,136,137,138,139,140,141,142,143,144,145,146,147,148,149,150,151,152,153,154,155,156,157,158,159,160,161,162,163,164,165,166,167,168,169,170,171,172,173,174,175,176,177,178,179,180,181,182,183,184,185,186,187,188,189,190,191,192,193,194,195,196,197,198,199,200,201,202,203,204,205,206,207,208,209,210,211,212,213,214,215,216,217,218,219,220,221,222,223,224,225,226,227,228,229,230,231] are cited in Supporting Information.
