# Supplementary material for: Decoding Sulfur-Containing Aroma Compounds in Foods: From Key Odorant Mapping to Structure–Odor Mechanisms and Flavor Design
Source: Foods. 2026 Aug 26;15(17):3003. doi: 10.3390/foods15173003 (PMC13564995; doi:10.3390/foods15173003)
Supplement: Supplementary file 1 [file foods-15-03003-s001.zip › foods-4469440-supplementary.pdf]

Decoding Sulfur-Containing Aroma Compounds in Foods: From Key Odorant Mapping to Structure–Odor Mechanisms and Flavor Design

Jinpeng Hu [1,†], Lulu Ma [1,†], Jiaying Huo [1], Jinyuan Sun [2], Shugang Li [1,*] and Hao Wang [3,4,*]

1 School of Food and Biological Engineering, Engineering Research Center of Bio-Process, Ministry of Education, Key Laboratory for Agricultural Products Processing of Anhui Province, Hefei University of Technology, Hefei 230601, China; hujinpeng06@163.com (J.H.); 2022800057@hfut.edu.cn (L.M.); huojy2023@hfut.edu.cn (J.H.)
2 China Food Flavor and Nutrition Health Innovation Center, Beijing Technology and Business University, Beijing 100048, China; sunjinyuan@btbu.edu.cn
3 Institute of Agro-Products Processing Research of Anhui Academy of Agricultural Sciences, Hefei 230031, China
4 Anhui Engineering Laboratory of Food Microbial Fermentation and Functional Application, Hefei 230031, China
* Correspondence: lishugang2020@hfut.edu.cn (S.L.); whdamonxin@163.com (H.W.)
† These authors contributed equally to this work.

1. Synthesis of stable isotope

Stable Isotope Dilution Assay（SIDA）, which employs stable isotope-labeled internal standards, can effectively compensate for losses during sample preparation and matrix effects, making it recognized as the "gold standard" for the quantitative analysis of aroma compounds. It is particularly suitable for the quantification of target analytes that are prone to loss or subject to strong matrix interference, such as sulfur-containing aroma substances. Given the significant role of SIDA in food flavor analysis, this paper summarizes the preparation methods of stable isotopes for important sulfur-containing odorants, including methyl sulfides, symmetric disulfides , asymmetric disulfides, thiols, and thiazoles. The specific synthetic procedures are as follows:

1.1 Synthesis of [$^2H_3$]-methional

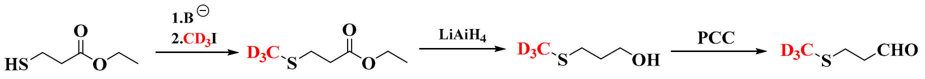

Figure S1. Flow Chart for the Synthesis of [$^2H_3$]-methional

Detailed Synthetic Procedures [46]**:** At 30 °C, a freshly prepared solution of tetrabutylammonium hydrogen sulfate (1.35 g, 4 mmol) and sodium hydroxide (8 mmol) in 8 mL water was added dropwise to a vigorously stirred solution of ethyl

3-mercaptopropionate (4 mmol) and [$^2H_3$]-methyl iodide in chloroform (8 mL); the reaction mixture was further stirred for 30 min, the phases were separated, the aqueous layer was extracted with chloroform (3×25 mL), the combined organic phases were concentrated, the residue was treated with ether to precipitate tetrabutylammonium iodide which was removed by filtration, the filtrate was washed with water, dried over Na_2SO_4, and distilled to afford pure 3-([$^2H_3$]methylmercapto)ethyl propionate (0.5 g, 83%), b.p. 87-89 °C/15 mmHg, MS(CI) = 152. Next, in a 100-mL three-necked flask fitted with a dropping funnel and an argon inlet, dried ether (15 mL) and pulverized lithium aluminium hydride (0.063 g) were placed; after stirring for 15 min at room temperature under an inert atmosphere to form a milky suspension, a solution of 3-([$^2H_3$]methylmercapto)ethyl propionate (3.3 mmol) was added via the dropping funnel at a rate to maintain gentle reflux, stirring was continued for another 2 h, the cooled reaction mixture was carefully treated with aqueous ether followed by water, the ether layer was separated, the solvent was removed by distillation at 55 °C in a water bath, and the residue was chromatographed on a silica gel column (maintained at 10 °C by a cooling jacket) with 30-40% ether in pentane to obtain [$^2H_3$]-methionol (0.270 g, 76%), a colourless oil with MS(CI) = 110. Finally, the ethyl ester function was first reduced to the alcohol derivative ([$^2H_3$]-methionol) with lithium aluminium hydride, and then oxidized with good efficiency using readily available pyridinium chlorochromate (PCC): in a 50-mL round-bottomed flask fitted with a drying tube, 0.8 g PCC was suspended in dry methylene chloride (10 mL), a solution of [$^2H_3$]-methionol (0.270 g) in methylene chloride (3 mL) was added quickly, after 2 h of thorough stirring, the reaction mixture was treated with ether (20 mL), the supernatant was decanted from the black residue, the insoluble residue was thoroughly washed with ether (3×25 mL), the combined organic solution was passed through a short pad of Florisil, the solvent was removed by distillation, and the residual oil was chromatographed on a silica gel column (maintained at 10 °C by a cooling jacket) with 2% ether in pentane to afford

the title compound [$^2H_3$]-methional (0.067 g), which was pure by HRGC analysis.

1.2 Synthesis of [$^2H_3$]-2-methyl-3-furanthiol or [$^2H_3$]Bis(2-methyl-3-furyl)disulfide

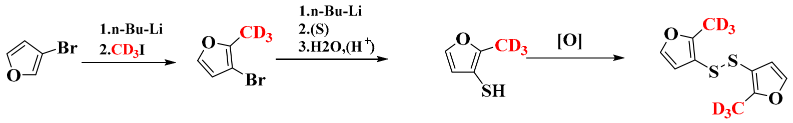

Figure S2. Flow Chart for the Synthesis of [$^2H_3$]-2-methyl-3-furanthiol or [$^2H_3$]-Bis(2-methyl-3-furyl)disulfide

Detailed Synthetic Procedures [46]**:** In a 100-ml three-necked flask, fitted with a condenser, a dropping funnel and filled with argon, were placed diisopropylamine (10mmol) in tetrahydrofuran (10 ml), freshly distilled from lithium aluminium hydride. At - 70 °C, n-butyllithium in hexane (10 mmol) was added to the reaction flask over a 5 min period via the dropping funnel, followed by the addition of 3-bromo furan (1.47 g, 10 mmol). Stirring was continued for 4 hat -70 °C and then [$^2H_3$]-methyl iodide (1.05 mol, 1.53 g) diluted in anhydrous tetrahydrofuran (3 ml) was added. After stirring over- night, the reaction mixture was finally poured into water and extracted with ether (3×50 ml). The combined ether extracts were washed with water, dried over Na_2SO_4 and, after removal of the ether, the residual brown oil was purified by distillation and compound [$^2H_3$]- 3-bromo-2-methylfuran isolated (1.2 g, 73%).Then, In a 100 ml three-necked flask, fitted with a dropping funnel and an argon inlet, were placed dried ether (10 ml) and the bromo derivative (1.2 g, 7.3 mmol). The mixture was stirred under argon and cooled to -70 °C in a solid CO_2-acetone bath while n-butyllithium (7.3 mmol) was added dropwise via the dropping funnel. The temperature of the mixture was held at - 70 °C and powdered sulfur crystals (0.250 g) were added in one aliquot to the well-stirred flask. After 1 h the temperature was allowed to rise to - 10 °C, whereupon the yellow solution was carefully poured into 50 ml rapidly stirred ice water to dissolve the lithium salt of the thiol. The ether layer was extracted with water (3×50 ml). The aqueous extracts were combined with the aqueous layer, the whole chilled and carefully acidified with

sulfuric acid (2 mol/L). The aqueous phase was immediately extracted with ether (3×50 ml), the combined ether extract was washed with water to remove trace amounts of acid and the extracts were then dried over Na_2SO_4. After removal of the ether, the residual oil was fractionated by means of a Fischer column. The yield was of 64% (0.54 g). [$^2H_3$]-2-methyl-3-furanthiol(20 mg), dissolved in a 5% aqueous solution of sodium hydroxide (5 ml), was oxidized by treatment with a potassium iodide/iodine solution in water. After quenching the reaction mixture with aqueous sodium thiosulfate (0.1 mol/L), the compound was extracted in ether. The organic extract was washed with water, dried over Na_2SO_4 and the solvent removed in vacuo to afford the disulfide [$^2H_3$]-Bis(2-methyl-3-furyl)disulfide quantitatively as a light yellow oil.

1.3 Synthesis of [$^2H_3$]-methyl alkyl disulfide

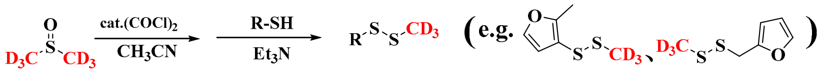

Figure S3. Flow Chart for the Synthesis of R-S-S-CD_3

Detailed Synthetic Procedures [44]**:** Into a solution of [$^2H_6$]-DMSO (30 mmol, 6 equiv.) in CH_3CN (12 mL), a solution of oxalyl chloride (3 mmol, 0.6equiv.) in CH_3CN (8 mL) was added dropwise at room temperature. Once the addition of oxalyl chloride was finished, the mixture was heated to reflux for 2 h in an oil bath.Following this, the mixture was cooled to room temperature. Then, Et_3N(10.5 mmol, 2.1equiv.) and thiol (5 mmol, 1 equiv.) were successively introduced. The resulting solution was stirred for 15 min. Subsequently, the reaction mixture was concentrated under vacuum, and the remaining material was subjected to purification using flash column chromatography (PE/EtOAc = 97:3). This procedure yielded the [$^2H_3$]-methyl alkyl disulfide.

1.4 Synthesis of [$^2H_6$]-3-mercapto-3-methylbutyl

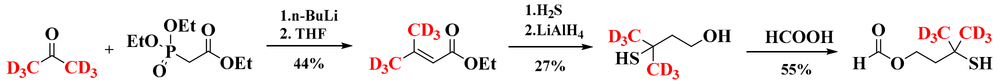

Figure S4. Flow Chart for the Synthesis of [$^2H_6$]-3-mercapto-3-methylbutyl

Detailed Synthetic Procedures [126]**:** Under an atmosphere of argon, n-butyl lithium in hexane (76 mL, 1.59 mol/L, 120.9 mmol) was added dropwise to a solution of diethylphosphonoacetic acid ethyl ester (40.63 g, 181.4 mmol) in THF (730 mL) at 0 °C within 20 min; after stirring for 20 min at 0 °C, a solution of [$^2H_6$]-acetone (7.74 g, 120.9 mmol) in THF (40 mL) was added dropwise at 0 °C over 10 min, followed by stirring at 0 °C for 1.5 h, warming to room temperature, stirring overnight, and pouring into water (800 mL). The aqueous phase was extracted with diethyl ether (800 mL × 2), and the organic layer was washed with brine, dried over $MgSO_4$, filtered, and concentrated under reduced pressure; the resulting residue (103 g) was distilled (bp 48–50 °C/2.1 kPa) to afford [$^2H_6$]-ethyl 3-methyl-2-butenoate as a colorless oil. To a solution of this product (6.27 g, 46.8 mmol) in dimethyl sulfoxide (DMSO, 13 g) at 0 °C were added diazabicyclo[5.4.0]undec-7-ene (DBU, 0.7 g, 4.6 mmol) and hydroquinone (0.25 g, 2.3 mmol), and hydrogen sulfide was bubbled through the solution at a rate of 50 mL/min for 2 h at 0 °C; the reaction mixture was then warmed to 40 °C, stirred overnight, and poured into 1 mol/L aqueous HCl (100 mL) at 10 °C. The aqueous phase was extracted with diethyl ether (100 mL × 2), and the organic layer was washed with saturated aqueous $NaHCO_3$ and brine, dried over Na_2SO_4, filtered, and concentrated under reduced pressure to give crude [$^2H_6$]-ethyl 3-mercapto-3-methylbutyrate as an oil, which was used directly in the next step without further purification. Under an atmosphere of argon, a solution of the crude [$^2H_6$]-ethyl 3-mercapto-3-methylbutyrate (8.83 g, 52.6 mmol) in diethyl ether (50 mL) was added dropwise to a suspension of lithium aluminum hydride (2.13 g, 55.9 mmol) in diethyl ether (150 mL) at 0 °C within 30 min; after stirring at 0 °C for 2 h, water (11 mL) was added dropwise at 0 °C over 5 min, followed by stirring at 10 °C for 40 min and addition of 2 mol/L aqueous HCl (250 mL). The organic layer was separated, the aqueous layer was extracted with diethyl ether (125 mL × 2), and the combined organic layers were dried over Na_2SO_4, filtered, and concentrated under reduced pressure; the residue (7.07 g) was distilled (bp 52 °C/5 kPa) to obtain

[$^{2}H_{6}$]-3-mercapto-3-methylbutanol as a colorless oil. Finally, [$^{2}H_{6}$]-3-mercapto-3-methylbutanol (0.88 g, 7.0 mmol) and formic acid (3.21 g, 69.8 mmol) were mixed at room temperature, stirred for 5 h at room temperature, diluted with diethyl ether (100 mL), washed with water and brine, dried over Na_2SO_4, filtered, and concentrated under reduced pressure; the residue (7.93 g) was distilled (bp 30–35 °C/3 kPa) to afford the target product [$^{2}H_{6}$]-3-mercapto-3-methylbutyl formate as a colorless oil (0.59 g; yield, 55%; purity, 98%).

1.5 Synthesis of [$^{2}H_{3}$]-4-mercapto-3-hexanone

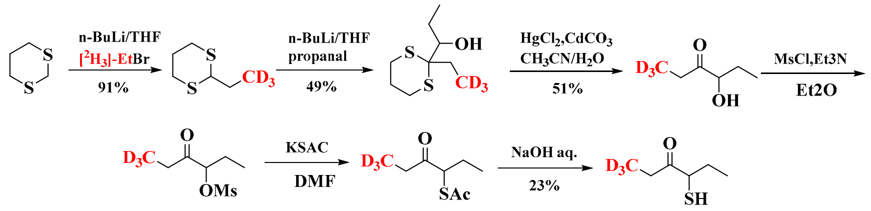

Figure S5. Flow Chart for the Synthesis of [$^{2}H_{3}$]-4-mercapto-3-hexanone

Detailed Synthetic Procedures [126]**:** Under an argon atmosphere, a hexane solution of n-butyl lithium (15.4 mL, 1.59 mol/L, 24.5 mmol) was added dropwise to a solution of 1,3-dithiane (2.9 g, 24.5 mmol) in tetrahydrofuran (THF, 25 mL) at −30 °C over 10 min; the mixture was stirred at −30 °C for 2 h, then a THF solution (5 mL) of [$^{2}H_{3}$]-bromoethane (2.5 g, 22.3 mmol) was added dropwise at −20 °C over 5 min. After stirring overnight at 0 °C in a refrigerator, the reaction mixture was warmed to room temperature and poured into ice water (50 mL), and the aqueous phase was extracted with diethyl ether (50 mL × 3). The combined organic layers were washed with brine, dried over magnesium sulfate ($MgSO_4$), filtered, and concentrated under high vacuum; the residue (4.2 g) was distilled (bp 71–72 °C/0.5 kPa) to afford [$^{2}H_{3}$]-2-ethyl-1,3-dithiane as an oil. Under an argon atmosphere, a hexane solution of n-butyl lithium (13.8 mL, 1.59 mol/L, 22.0 mmol) was added dropwise to a solution of the above-prepared [$^{2}H_{3}$]-2-ethyl-1,3-dithiane (3.1 g, 20.0 mmol) in THF (100 mL) at −30 °C over 10 min; the mixture was stirred at −30 °C for 2 h, then a THF solution (5 mL) of propanal (1.7 g, 30.0 mmol) was added dropwise at -20 °C within 5 min.

After stirring overnight at 0 °C in a refrigerator, the reaction mixture was warmed to room temperature and poured into ice water (50 mL), and the aqueous phase was extracted with diethyl ether (100 mL × 3). The combined organic layers were washed with brine, dried over $MgSO_4$, filtered, and concentrated under high vacuum; the residue (4.6 g) was purified by silica gel chromatography (150 g, eluent: hexane/ethyl acetate = 15:1, v/v), and the target-containing fractions were combined and concentrated under high vacuum to afford [$^2H_3$]-1-(2-ethyl-1,3-dithian-2-yl)propan-1-ol as an oil. Under a nitrogen atmosphere, mercury(II) chloride ($HgCl_2$, 5.4 g, 20.0 mmol), cadmium carbonate ($CdCO_3$, 3.4 g, 20.0 mmol), and the above [$^2H_3$]-1-(2-ethyl-1,3-dithian-2-yl)propan-1-ol (2.1 g, 9.8 mmol) were dissolved in a water/acetonitrile mixture (1:10, v/v, 110 mL), and the mixture was stirred at 50–60 °C for 2 h. After cooling to room temperature, the reaction mixture was diluted with diethyl ether (500 mL), filtered through Celite, and the filtrate was washed with brine, dried over $MgSO_4$, filtered, and concentrated under high vacuum; the residue (1.0 g) was purified by silica gel chromatography (45 g, eluent: hexane/ethyl acetate = 10:1 to 3:1, v/v), and the target-containing fractions were combined and concentrated under high vacuum to afford [$^2H_3$]-4-hydroxy-3-hexanone as an oil. [$^2H_3$]-4-Methanesulfonyloxy-3-hexanone was prepared by reacting [$^2H_3$]-4-hydroxy-3-hexanone with methanesulfonyl chloride according to the synthesis protocol of 4-methanesulfonyloxy-3-hexanone reported in our previous study, and the crude product was used directly in the next step. Subsequently, [$^2H_3$]-4-acetylthio-3-hexanone was obtained by treating the crude [$^2H_3$]-4-methanesulfonyloxy-3-hexanone with potassium thioacetate according to the synthesis protocol of 4-acetylthio-3-hexanone reported in our previous study (0.53 g, crude product used directly). Finally, [$^2H_3$]-4-mercapto-3-hexanone was prepared by treating the crude [$^2H_3$]-4-acetylthio-3-hexanone with aqueous sodium hydroxide (NaOH) according to the synthesis protocol of 4-mercapto-3-hexanone reported in our previous study (156 mg; yield, 23%; purity, 97%).

1.6 Synthesis of [$^2H_4$]-2-acetyl-2-thiazoline

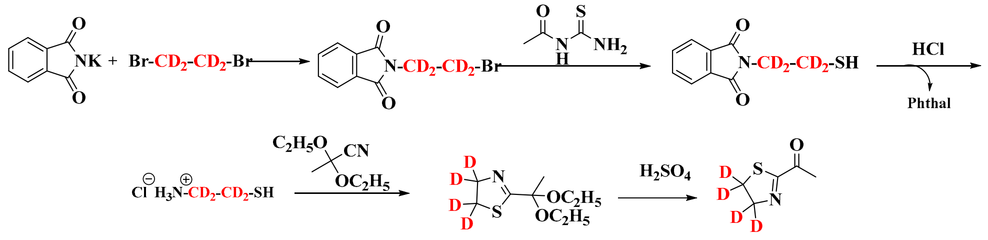

Figure S6. Flow Chart for the Synthesis of [$^2H_4$]-2-acetyl-2-thiazoline

Detailed Synthetic Procedures [127]**:** A mixture of [$^2H_4$]-bromoethyl phthalimide (52mmol, 13.4g) and acetylthiourea (58 mmol, 6.85 g) in absolute ethanol (50 ml) was refluxed for 24 h. The solvent was removed in vacuo and the residue, which contained [$^2H_4$]-mercaptoethyl phthalimlde, was dissolved in diethyl ether (10 ml) and then applied onto a column (30×1 cm) with $A1_2O_3$ (activity II, 250 g). The column was washed with diethyl ether (500 ml) and the solvent was evaporated at reduced pressure from the effluent. The dry residue was suspended in aqueous HC1 (20%, w/w, 50 ml) and refluxed for 24 h. The phthalic acid formed was removed by filtration, and the water by distillation under reduced pressure. The residue containing [$^2H_4$]-cysteamine. HC1 was taken up in methanol (1 ml) and the pH of the solution was adjusted to 10.0 by addition of methanolic KOH (20%, w/w). After filtration, ammo-nium acetate (13 mmol, 1 g) and 2,2-diethoxypropionitrile (3.5 mmol, 500 rag) were added, and the mixture was stirred for 18 h at room temperature and for a further 6 h at 70 °C. Most of the methanol was removed in vacuo, and the residue was treated with a cooled (0 °C) mixture consisting of KOH (0.9 g), water (8 ml) and diethyl ether (2 ml). The organic layer was separated, and the aque- ous phase extracted with diethyl ether (4×5 ml). Removal of the ether yielded a residue which was dropped into cooled (0 °C) H_2SO_4 (96%, w/w, 4 ml). The solution was left stirring for 15 rain and then dropped into a mixture of $NaHCO_3$ (17 g), ice (44 g) and diethyl ether (6 ml). Ice-water (50 ml) was added, and the [$^2H_4$]-2-acetyl-2-thiazoline was extracted with diethyl ether (5×15 ml). High-resolution gas chromatography (HRGC) on capillary

OV-1701 showed that the [$^{2}H_4$]-acetyl-2-thiazoline obtained was sufficiently pure.

2. Systematic Review (Meta-Analysis) of Key SACs

To clarify the key role of SACs in the aromas of different foods, a systematic meta-analysis was conducted based on a literature review. The literature collection for this systematic review comprised two distinct sources: (i) the quality-oriented dataset previously established by Hofmann and co-workers [2] , and (ii) a new systematic search updating the dataset to the present.

Source 1: Hofmann's original dataset (1980-2013). Following the quality-oriented strategy established by Hofmann and co-workers, we first screened the 117 publications that met the pre-specified criteria (i)-(iii) in the original meta-analysis. From these, 42 publications reporting at least one sulfur-containing compound among the identified key odorants were retained for this review.

Source 2: New systematic search (2013-2026). To update the dataset, a systematic literature search was conducted on May 1, 2026, in ScienceDirect (https://www.sciencedirect.com/), PubMed (https://pubmed.ncbi.nlm.nih.gov/), and Web of Science (https://www.webofscience.com/). The following search string was used: ("aroma analysis" or "flavor analysis" and "SIDA") and "food", with the time range restricted to 2013-2026. The initial retrieval yielded 97 records from PubMed, 95 records from Web of Science, and 74 records from ScienceDirect. After removing duplicates, 163 unique records remained. The retained publications were screened to ensure that at least one sulfur-containing compound was reported among the identified key odorants, resulting in 59 studies being retained for inclusion in the dataset.

The remaining publications underwent a two-stage screening process. In the first stage, titles and abstracts were screened against the following pre-specified inclusion criteria adapted from Hofmann et al.: (i) bioactivity-directed localization of most

intense odorants by means of GC-O analysis; (ii) identification of key odorants by comparing chromatographic retention time, mass spectrometric, and sensory data with those of independently synthesized reference compounds; and (iii) comprehensive quantitation of the entire set of key odorants using stable isotope dilution analysis (SIDA). Studies employing non-SIDA quantification methods (e.g., external standardization, standard addition, or generic internal standard calibration without isotopically labeled analogues) were excluded, because these methods cannot adequately correct for the severe recovery losses and matrix effects characteristic of volatile sulfur-containing compounds, thereby compromising the accuracy of odor activity value calculations. In the second stage, full texts of the retained publications were screened to ensure that at least one sulfur-containing compound was reported among the identified key odorants.

Ultimately, 101 publications from 1980 to 2026 were obtained, covering studies on key food odorants in 221 food samples (including alcoholic beverages, meat products, aquatic products, grain baked foods, dairy products, oilseeds, fruits, vegetables, mushrooms, herbs, cocoa and chocolate, coffee, tea, soy sauce, and other various foods). The specific key SACs and relevant references are as follows:

Table. S1. List of Key SACs in 221 food samples, including their CAS numbers, odor properties, stable isotope-labeled analogs, odor thresholds, odor activity value (OAV), molecular structures, and literature sources.

No.	Compound	CAS No.	Odor	Labeled Standards	OT	OAV	Structure	Foods	Refs.
1	methanethiol	74-93-1	cabbage-like, sulphury	[$^2H_3$]-methanethiol	0.59 μg/L in water	12	—SH	Longjing tea	[128]
	methanethiol	74-93-1	cabbage-like, sulphury	[$^2H_3$]-methanethiol	0.4 μg/kg in oil	3	—SH	Milk Chocolate	[129]
	methanethiol	74-93-1	sulfury	--[a]	0.59 μg/kg in water	10	—SH	Chicken Egg	[130]
	methanethiol	74-93-1	sulfury	--[a]	0.59 μg/kg in water	30	—SH	Plant-Based Egg	[130]
	methanethiol	74-93-1	cabbage-like, sulfury	[$^2H_3$]-methanethiol	0.36 μg/kg in oil	31	—SH	Raw rapeseeds	[131]
	methanethiol	74-93-1	cabbage-like, sulfury	[$^2H_3$]-methanethiol	0.36 μg/kg in oil	617	—SH	Rosasted rapeseeds	[131]
	methanethiol	74-93-1	rotten, sulfurous, cabbage	[$^2H_3$]-methanethiol	0.02 μg/kg in water	2910	—SH	Thai Fish Sauce A	[132]
	methanethiol	74-93-1	rotten, sulfurous, cabbage	[$^2H_3$]-methanethiol	0.02 μg/kg in water	2853	—SH	Thai Fish Sauce B	[132]
	methanethiol	74-93-1	cabbage-like, sulfury	[$^2H_3$]-methanethiol	0.1 μg/kg in starch	290	—SH	Freshly Prepared Oat Pastry	[133]
	methanethiol	74-93-1	sulfury	[$^2H_3$]-methanethiol	0.06 μg/kg in sunflower oil	2316	—SH	Pumpkin Seed Oil A	[134]
	methanethiol	74-93-1	sulphurous	[$^2H_3$]-methanethiol	0.06 μg/kg in oil	7270	—SH	Gruyère cheeses with a potato-like off-flavour	[135]

Continued

No.	Compound	CAS No.	Odor	Labeled Standards	OT	OAV	Structure	Foods	Refs.
1	methanethiol	74-93-1	sulphurous	$[^2H_3]$-methanethiol	0.06 µg/kg in oil	11300	—SH	Gruyère cheeses without an off-flavour	[135]
	methanethiol	74-93-1	sulfurous	$[^2H_3]$-methanethiol	0.06 µg/kg in sunflower oil were nasally estimated	4333	—SH	Camembert Cheese Samples I	[136]
	methanethiol	74-93-1	sulfurous	$[^2H_3]$-methanethiol	0.06 µg/kg in sunflower oil were nasally estimated	4538	—SH	Camembert Cheese Samples II	[136]
	methanethiol	74-93-1	sulphurous	$[^2H_3]$-methanethiol	0.2 µg/kg in water	29	—SH	Dry spinach I	[137]
	methanethiol	74-93-1	sulphurous	$[^2H_3]$-methanethiol	0.2 µg/kg in water	13	—SH	Dry spinach II	[137]
	methanethiol	74-93-1	sulphurous	$[^2H_3]$-methanethiol	0.06 µg/kg in sunflower oil	1889	—SH	Pan-Roasted Peanut Meal	[138]
	methanethiol	74-93-1	sulphurous	$[^2H_3]$-methanethiol	0.36 µg/kg in oil	50	—SH	Boiled Cod Homogenates C	[139]
	methanethiol	74-93-1	sulphurous	$[^2H_3]$-methanethiol	0.36 µg/kg in oil	65	—SH	Boiled Cod Homogenates D	[139]

Continued

No.	Compound	CAS No.	Odor	Labeled Standards	OT	OAV	Structure	Foods	Refs.
1	methanethiol	74-93-1	sulphurous	[$^{2}H_{3}$]-methanethiol	0.2 µg/kg in water	1300	—SH	Parsley (Hamburger Schnitt)	[140]
	methanethiol	74-93-1	sulphurous	[$^{2}H_{3}$]-methanethiol	0.2 µg/kg in water	875	—SH	Parsley (Mooskrause')	[140]
	methanethiol	74-93-1	sulphurous，cabbage-like	[$^{2}H_{3}$]-methanethiol	0.2 µg/kg in water	335	—SH	Dry parsley I	[141]
	methanethiol	74-93-1	sulphurous，cabbage-like	[$^{2}H_{3}$]-methanethiol	0.2 µg/kg in water	10	—SH	Dry parsleyV	[141]
	methanethiol	74-93-1	cabbage-like	[$^{2}H_{3}$]-methanethiol	0.2 µg/kg in water	1050	—SH	Ground Roasted Arabica Coffee	[142]
	methanethiol	74-93-1	cabbage-like	[$^{2}H_{3}$]-methanethiol	0.2 µg/kg in water	3000	—SH	Ground Roasted Robusta Coffee	[142]
	methanethiol	74-93-1	sulphurousputrid	[$^{2}H_{3}$]-methanethiol	0.2 µg/kg in water	7280	—SH	Cooked chicken A	[143]
	methanethiol	74-93-1	sulphurousputrid	[$^{2}H_{3}$]-methanethiol	0.2 µg/kg in water	7275	—SH	Cooked chicken B	[143]
	methanthiol	74-93-1	sulfurous	[$^{2}H_{3}$]-methanethiol	0.59 µg/kg in water	500	—SH	Fresh boiled cod	[144]
	methanthiol	74-93-1	sulfurous	[^{2}H3]-methanethiol	0.59 µg/kg in water	650	—SH	Stored boiled cod	[144]

Continued

No.	Compound	CAS No.	Odor	Labeled Standards	OT	OAV	Structure	Foods	Refs.
1	methanthiol	74-93-1	sulfurous	[$^2H_3$]-methanethiol	0.59 μg/kg in water	500	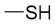	Fresh boiled cod	[144]
	methanthiol	74-93-1	sulfurous	[^{2}H3]-methanethiol	0.59 μg/kg in water	650	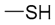	Stored boiled cod	[144]
	methanethiol	74-93-1	sulfurous	[$^2H_3$]-methanethiol	0.59 μg/kg in water	4	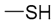	Fried Prawn Meat	[145]
	methanethiol	74-93-1	cabbage-like, sulfury	[$^2H_3$]-methanethiol	0.59 μg/kg in water	14	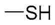	Fresh Jingshan cha Green Tea Infusion	[146]
2	ethanethiol	75-08-1	rotten onion	[$^2H_5$]-ethanethiol,	0.095 μg/kg in water	480000	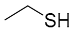	Monthong Durian Pulp	[147]
3	dimethyl sulfide	75-18-3	asparagus-like, putrid	[$^2H_6$]-dimethyl sulfide	0.3 μg/L in water	440	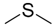	Longjing tea	[128]
	dimethyl sulfide	75-18-3	asparagus-like	[$^2H_6$]-dimethyl sulfide	0.26 μg/kg in oil	7	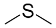	Rapeseed oil	[148]
	dimethyl sulfide	75-18-3	asparagus-like, putrid	[$^2H_6$]-dimethyl sulfide	0.3 μg/kg in water	1100	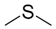	Toona sinensis (Xiang chun)	[149]
	dimethyl sulfide	75-18-3	asparagus-like	[$^2H_6$]-dimethyl sulfide	2.6 μg/kg in oil	151	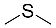	Raw rapeseeds	[131]
	dimethyl sulfide	75-18-3	asparagus-like	[$^2H_6$]-dimethyl sulfide	2.6 μg/kg in oil	298	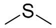	Rosasted rapeseeds	[131]
	dimethyl sulfide	75-18-3	cabbage	[$^2H_6$]-dimethyl sulfide	1.0 μg/kg in water	2	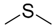	Pumpernickel Bread	[150]
	dimethyl sulfide	75-18-3	corn	[$^2H_6$]-dimethyl sulfide	0.3 μg/kg in water	134	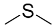	Thai Fish Sauce A	[132]

Continued

No.	Compound	CAS No.	Odor	Labeled Standards	OT	OAV	Structure	Foods	Refs.
3	dimethyl sulfide	75-18-3	corn	[$^2H_6$]-dimethyl sulfide	0.3 µg/kg in water	34	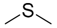	Thai Fish Sauce B	[132]
	dimethyl sulfide	75-18-3	corn	[$^2H_6$]-dimethyl sulfide	0.3 µg/kg in water	237000	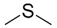	Native spearmint oils	[151]
	dimethyl sulfide	75-18-3	corn	[$^2H_6$]-dimethyl sulfide	0.3 µg/kg in water	91000	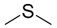	Scotch spearmint oils	[151]
	dimethyl sulfide	75-18-3	corn	[$^2H_6$]-dimethyl sulfide	0.3 µg/kg in water	36300	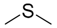	Macho mint oils	[151]
	dimethyl sulfide	75-18-3	corn	[$^2H_6$]-dimethyl sulfide	0.3 µg/kg in water	12	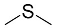	Roasted Chicory “Coffee” Brews	[152]
	dimethyl sulfide	75-18-3	cabbage	[$^2H_6$]-dimethyl sulfide	7.6 µg/L in water	16	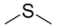	Fermented one day Tempeh	[153]
	dimethyl sulfide	75-18-3	cabbage	[$^2H_6$]-dimethyl sulfide	7.6 µg/L in water	16	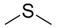	Fried Fermented one day Tempeh	[153]
	dimethyl sulfide	75-18-3	cabbage	[$^2H_6$]-dimethyl sulfide	7.6 µg/L in water	17	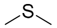	Fermented five days Tempeh	[153]
	dimethyl sulfide	75-18-3	cabbage	[$^2H_6$]-dimethyl sulfide	7.6 µg/L in water	19	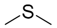	Fried Fermented five days Tempeh	[153]
	dimethyl sulfide	75-18-3	sulphurous	[$^2H_6$]-dimethyl sulfide	0.12 µg/kg in water	36	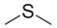	Freshly reconstituted orange juice A	[154]

Continued

No.	Compound	CAS No.	Odor	Labeled Standards	OT	OAV	Structure	Foods	Refs.
3	dimethyl sulfide	75-18-3	sulphurous	[$^2H_6$]-dimethyl sulfide	0.12 μg/kg in water	350	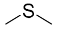	Freshly reconstituted orange juice B	[154]
	dimethyl sulfide	75-18-3	canned maize	[$^2H_6$]-dimethyl sulfide	1.0 μg/kg in water	59	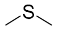	Bavarian Pilsner-type Beer	[155]
	dimethyl sulfide	75-18-3	sulfurous, garlic-like	[$^2H_6$]-dimethyl sulfide	1.2 μg/kg in sunflower oil were nasally estimated	208	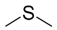	Camembert Cheese Samples I	[136]
	dimethyl sulfide	75-18-3	sulfurous, garlic-like	[$^2H_6$]-dimethyl sulfide	1.2 μg/kg in sunflower oil were nasally estimated	341	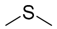	Camembert Cheese Samples II	[136]
	dimethyl sulfide	75-18-3	cabbage-like,spicy	[$^2H_6$]-dimethyl sulfide	0.3 μg/kg in water	45000	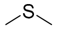	Dry spinach I	[137]
	dimethyl sulfide	75-18-3	cabbage-like,spicy	[$^2H_6$]-dimethyl sulfide	0.3 μg/kg in water	1667	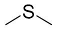	Dry spinach II	[137]
	dimethyl sulfide	75-18-3	cabbage-like,spicy	[$^2H_6$]-dimethyl sulfide	10 μg/L in water/ethanol (90 + 10, w/w)	1	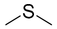	Gewürztraminer White Wine	[156]
	dimethyl sulfide	75-18-3	cabbage-like,spicy	[$^2H_6$]-dimethyl sulfide	2.6 μg/kg in oil	39	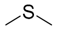	Boiled Cod Homogenates C	[139]

Continued

No.	Compound	CAS No.	Odor	Labeled Standards	OT	OAV	Structure	Foods	Refs.
3	dimethyl sulfide	75-18-3	cabbage-like,spicy	[$^{2}H_{6}$]-dimethyl sulfide	2.6 µg/kg in oil	13	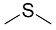	Boiled Cod Homogenates D	[139]
	dimethyl sulfide	75-18-3	sulphurous，cabbage-like	[$^{2}H_{6}$]-dimethyl sulfide	1.0 µg/kg in water	75000	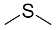	Dry parsley I	[141]
	dimethyl sulfide	75-18-3	sulphurous，cabbage-like	[$^{2}H_{6}$]-dimethyl sulfide	1.0 µg/kg in water	3000	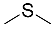	Dry parsley V	[141]
	dimethyl sulfide	75-18-3	cabbage-like	[$^{2}H_{6}$]-dimethyl sulfide	0.3 µg/kg in water	260	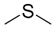	Fresh boiled cod	[144]
	dimethyl sulfide	75-18-3	cabbage-like	[$^{2}H_{6}$]-dimethyl sulfide	0.3 µg/kg in water	83	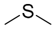	Stored boiled cod	[144]
	dimethyl sulfide	75-18-3	Cooked corn-like	[$^{2}H_{3}$]-dimethyl sulfide	0.3 µg/kg in water	4270	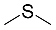	Fresh leaves of large-leaf yellow tea	[157]
	dimethyl sulfide	75-18-3	Cooked corn-like	[$^{2}H_{3}$]-dimethyl sulfide	0.3 µg/kg in water	12,900	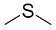	Fixing of large-leaf yellow tea	[157]
	dimethyl sulfide	75-18-3	Cooked corn-like	[$^{2}H_{3}$]-dimethyl sulfide	0.3 µg/kg in water	6230	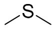	First yellowing of large-leaf yellow tea	[157]
	dimethyl sulfide	75-18-3	Cooked corn-like	[$^{2}H_{3}$]-dimethyl sulfide	0.3 µg/kg in water	6270	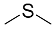	Rolling of large-leaf yellow tea	[157]
	dimethyl sulfide	75-18-3	Cooked corn-like	[$^{2}H_{3}$]-dimethyl sulfide	0.3 µg/kg in water	11500	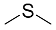	First roasting of large-leaf yellow tea	[157]

Continued

No.	Compound	CAS No.	Odor	Labeled Standards	OT	OAV	Structure	Foods	Refs.
3	dimethyl sulfide	75-18-3	Cooked corn-like	[$^2H_3$]-dimethyl sulfide	0.3 µg/kg in water	6400	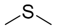	Second yellowing of large-leaf yellow tea	[157]
	dimethyl sulfide	75-18-3	Cooked corn-like	[$^2H_3$]-dimethyl sulfide	0.3 µg/kg in water	46700	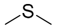	Second roasting of large-leaf yellow tea	[157]
	dimethyl sulfide	75-18-3	Cooked corn-like	[$^2H_3$]-dimethyl sulfide	0.3 µg/kg in water	3930	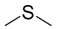	Third yellowing of large-leaf yellow tea	[157]
	dimethyl sulfide	75-18-3	Cooked corn-like	[$^2H_3$]-dimethyl sulfide	0.3 µg/kg in water	71700	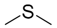	Tthird roasting at low-fire of largeyellow tea-leaf	[157]
	dimethyl sulfide	75-18-3	Cooked corn-like	[$^2H_3$]-dimethyl sulfide	0.3 µg/kg in water	79,700	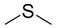	Tthird roasting at medium-fire of large-leaf yellow tea	[157]
	dimethyl sulfide	75-18-3	Sweet	[$^2H_6$]-dimethyl sulfide	0.84 µg/kg in water in water	115	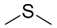	Ripe Capparis spinosa L. fruit Before storage	[158]
	dimethyl sulfide	75-18-3	Sweet	[$^2H_6$]-dimethyl sulfide	0.84 µg/kg in water in water	63	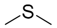	Ripe Capparis spinosa L. fruit After storage	[158]
	dimethyl sulfide	75-18-3	cabbage-like	[$^2H_6$]-dimethyl sulfide	2.6 µg/kg in oil	7	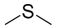	Native Cold-Pressed Rapeseed Oils PC	[159]
	dimethyl sulfide	75-18-3	cabbage-like	[$^2H_6$]-dimethyl sulfide	2.6 µg/kg in oil	2	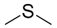	Native Cold-Pressed Rapeseed Oils OF	[159]

Continued

No.	Compound	CAS No.	Odor	Labeled Standards	OT	OAV	Structure	Foods	Refs.
3	dimethyl sulfide	75-18-3	cabbage-like	$[^2H_6]$-dimethyl sulfide	2.6 μg/kg in oil	2	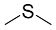	Rapeseeds OFS	[159]
	dimethyl sulfide	75-18-3	cabbage-like	$[^2H_6]$-dimethyl sulfide	0.74 μg/kg in water	82	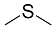	White Alba Truffle	[160]
	dimethyl sulfide	75-18-3	cabbage-like	$[^2H_6]$-dimethyl sulfide	0.74 μg/kg in water	500	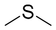	Burgundy Truffle	[160]
	dimethyl sulfide	75-18-3	cabbage	$[^2H_5]$-linalool[b]	0.3μg/Lin water	716	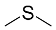	Unfermented black tea	[161]
	dimethyl sulfide	75-18-3	cabbage	$[^2H_5]$-linalool[b]	0.3μg/Lin water	427	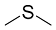	Underfermented black tea	[161]
	dimethyl sulfide	75-18-3	cabbage	$[^2H_5]$-linalool[b]	0.3μg/Lin water	234	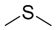	Fully fermented black tea	[161]
	dimethyl sulfide	75-18-3	cabbage	$[^2H_5]$-linalool[b]	0.3μg/Lin water	184	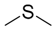	Overfermented black tea	[161]
	dimethyl sulfide	75-18-3	asparagus-like, putrid	$[^2H_6]$-dimethyl sulfide	0.3 μg/kg in water	458	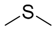	Fresh Jingshan cha Green Tea Infusion	[146]
	dimethyl sulfide	75-18-3	cabbage-like	$[^2H_6]$-dimethyl sulfide	0.3 μg/kg in water	150 00 0	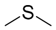	Freeze-dried green T. sinensis	[162]
	dimethyl sulfide	75-18-3	cabbage-like	$[^2H_6]$-dimethyl sulfide	0.3 μg/kg in water	95 000	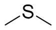	Solar-dried green T. sinensis	[162]
	dimethyl sulfide	75-18-3	cabbage-like	$[^2H_6]$-dimethyl sulfide	0.3 μg/kg in water	26 000	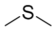	Oven-dried green T. sinensis	[162]

Continued

No.	Compound	CAS No.	Odor	Labeled Standards	OT	OAV	Structure	Foods	Refs.
3	dimethyl sulfide	75-18-3	cabbage-like	$[^{2}H_{6}]$-dimethyl sulfide	0.3 μg/kg in water	280 000	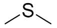	Freeze-dried red T. sinensis	[162]
	dimethyl sulfide	75-18-3	cabbage-like	$[^{2}H_{6}]$-dimethyl sulfide	0.3 μg/kg in water	87 000	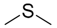	Solar-dried red T. sinensis	[162]
	dimethyl sulfide	75-18-3	cabbage-like	$[^{2}H_{6}]$-dimethyl sulfide	0.3 μg/kg in water	77 000	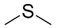	Oven-dried red T. sinensis	[162]
	dimethyl sulfide	75-18-3	cabbage-like	$[^{2}H_{6}]$-dimethyl sulfide	0.3 μg/kg in water	437	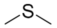	High grades keemun black tea	[163]
	dimethyl sulfide	75-18-3	cabbage-like	$[^{2}H_{6}]$-dimethyl sulfide	0.3 μg/kg in water	39	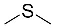	Low grades keemun black tea	[163]
	dimethyl sulfide	75-18-3	cabbage-like	$[^{2}H_{6}]$-dimethyl sulfide	1 μg/kg in water	377	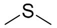	4 days of ripening cottage cheese	[164]
	dimethyl sulfide	75-18-3	cabbage-like	$[^{2}H_{6}]$-dimethyl sulfide	1 μg/kg in water	13	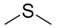	Fried cottage cheese	[164]
4	2-furanmethanethiol	98-02-2	coffee-like	$[^{2}H_{3}]$-2-furanmethanethiol	0.019 μg/kg in oil	149	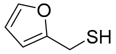	Raw rapeseeds	[131]
	2-furanmethanethiol	98-02-2	coffee-like	$[^{2}H_{3}]$-2-furanmethanethiol	0.019 μg/kg in oil	36300	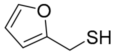	Rosasted rapeseeds	[131]
	2-furanmethanethiol	98-02-2	roasted	--[a]	0.01 μg/kg in water	1901	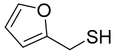	Roasted Goose	[45]
	2-furanmethanethiol	98-02-2	coffee-like	$[^{2}H_{2}]$-2-furanmethanethiol	0.37 μg/kg in oil	86	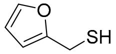	Roasted Hazelnut Paste	[165]

Continued

No.	Compound	CAS No.	Odor	Labeled Standards	OT	OAV	Structure	Foods	Refs.
4	2-furanmethanethiol	98-02-2	sulfury, burnt	[$^{2}H_{2}$]-2-furanmethanethiol	0.37 µg/kg in oil	8	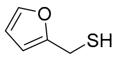	Raw Hazelnuts B	[166]
	2-furanmethanethiol	98-02-2	sulfury, burnt	[$^{2}H_{2}$]-2-furanmethanethiol	0.37 µg/kg in oil	15	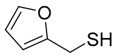	Roasted Hazelnuts	[166]
	2-furanmethanethiol	98-02-2	sulfury, burnt	[$^{2}H_{2}$]-2-furanmethanethiol	0.4 µg/kg in sunflower oil	6	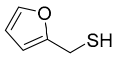	Pumpkin Seed Oil A	[134]
	2-furanmethanethiol	98-02-2	roasty, coffeelike	[$^{2}H_{2}$]-2-furanmethanethiol	0.4 µg/kg in sunflower oil	135	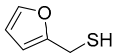	Roasted Sesame Seeds	[167]
	2-furanmethanethiol	98-02-2	roasty,coffee-like	[$^{2}H_{2}$]-2-furanmethanethiol	0.01 µg/kg in water	58000	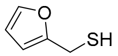	Thermally Treated Commercial Yeast Extract	[168]
	2-furanmethanethiol	98-02-2	roasty,coffee-like	[$^{2}H_{2}$]-2-furanmethanethiol	0.01 µg/kg in water	2900	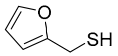	Thermally Treated Self-Prepared Extract of Bakers' Yeast	[168]
	2-furanmethanethiol	98-02-2	roasty,coffee-like	[$^{2}H_{2}$]-2-furanmethanethiol	0.01µg/kg in water	58000	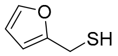	Commercial Yeast Extract (Glucose)	[168]
	2-furanmethanethiol	98-02-2	roasty,coffee-like	[$^{2}H_{2}$]-2-furanmethanethiol	0.01 µg/kg in water	12200	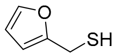	Commercial Yeast Extract (2-Oxopropanal)	[168]
	2-furanmethanethiol	98-02-2	roasty,coffee-like	[$^{2}H_{2}$]-2-furanmethanethiol	0.01 µg/kg in water	10	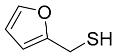	Thermal Treatment of Water-Soluble Low-MW Fractions from Bakers' Yeast A	[168]

Continued

No.	Compound	CAS No.	Odor	Labeled Standards	OT	OAV	Structure	Foods	Refs.
4	2-furanmethanethiol	98-02-2	roasty,coffee-like	$[^2H_2]$-2-furanmethanethiol	0.01 μg/kg in water	10	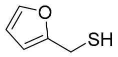	Thermal Treatment of Water-Soluble Low-MW Fractions from Bakers' Yeast B	[168]
	2-furanmethanethiol	98-02-2	roasty,coffee-like	$[^2H_2]$-2-furanmethanethiol	0.01 μg/kg in water	110000	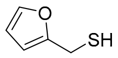	Arabica roasted coffee	[169]
	2-furanmethanethiol	98-02-2	roasty,coffee-like	$[^2H_2]$-2-furanmethanethiol	0.01 μg/kg in water	170000	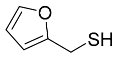	Robusta roasted coffee	[169]
	2-furanmethanethiol	98-02-2	roasty,coffee-like	$[^2H_2]$-2-furanmethanethiol	0.4 μg/kg in sunflower oil	22	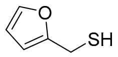	Pan-Roasted Peanut Meal	[138]
	2-furanmethanethiol	98-02-2	roasty, sulfurous	$[^2H_2]$-2-furanmethanethiol	0.4 μg/kg in oil	4	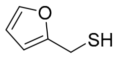	Hungarian-Type Salami	[170]
	2-furanmethanethiol	98-02-2	coffee-like	$[^2H_2]$-2-furanmethanethiol	0.01 μg/kg in water	1910	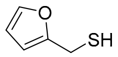	Ground Roasted Arabica Coffee	[142]
	2-furanmethanethiol	98-02-2	coffee-like	$[^2H_2]$-2-furanmethanethiol	0.01 μg/kg in water	3900	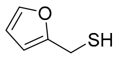	Ground Roasted Robusta Coffee	[142]
	2-furanmethanethiol	98-02-2	sulphurousroasty	$[^2H_2]$-2-furanmethanethiol	0.01 μg/kg in water	450	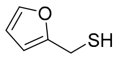	Cooked chicken A	[143]

Continued

No.	Compound	CAS No.	Odor	Labeled Standards	OT	OAV	Structure	Foods	Refs.
4	2-furanmethanethiol	98-02-2	Sulphurousroasty	[$^2H_2$]-2-furanmethanethiol	0.01 µg/kg in water	80	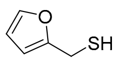	Cooked chicken B	[143]
	2-furanmethanethiol	98-02-2	coffee-like	[$^2H_2$]-2-furanmethanethiol	0.4 µg/kg in sunflower oil	1225	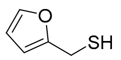	White sesame seeds (180°C)	[171]
	2-furanmethanethiol	98-02-2	coffee-like	[$^2H_2$]-2-furanmethanethiol	0.4 µg/kg in sunflower oil	3165	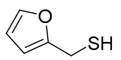	White sesame seeds (200°C)	[171]
	2-furanmethanethiol	98-02-2	coffee-like	[$^2H_2$]-2-furanmethanethiol	0.4 µg/kg in sunflower oil	5080	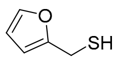	White sesame seeds (220°C)	[171]
	2-furanmethanethiol	98-02-2	coffee-like	[$^2H_2$]-2-furanmethanethiol	0.4 µg/kg in sunflower oil	5235	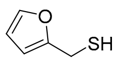	White sesame seeds (240°C)	[171]
	2-furanmethanethiol	98-02-2	coffee-like	[$^2H_2$]-2-furanmethanethiol	0.4 µg/kg in sunflower oil	1682	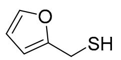	Black sesame seeds	[171]
	2-furanmethanethiol	98-02-2	coffee-like	[$^2H_2$]-2-furanmethanethiol	0.4 µg/kg in sunflower oil	6152	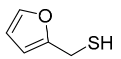	White sesame seeds	[171]
	2-furanmethanethiol	98-02-2	roasted coffee	[$^2H_5$]-2-furanmethanethiol	0.4 ng/L in a synthetic wine matrix solution	83	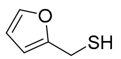	Carmenere Red Wines CMR 1	[172]
	2-furanmethanethiol	98-02-2	roasted coffee	[$^2H_5$]-2-furanmethanethiol	0.4 ng/L in a synthetic wine matrix solution	80	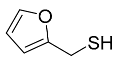	Carmenere Red Wines CMR 3	[172]
	2-furanmethanethiol	98-02-2	roasted coffee	[$^2H_5$]-2-furanmethanethiol	0.4 ng/L in a synthetic wine matrix solution	97	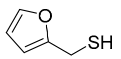	Carmenere Red Wines CMR 4	[172]

Continued

No.	Compound	CAS No.	Odor	Labeled Standards	OT	OAV	Structure	Foods	Refs.
4	2-furanmethanethiol	98-02-2	coffee	--[a]	0.036 µg/kg in water	610	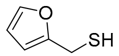	Enzymatic Hydrolysis of Mushroom Protein and Cysteine	[173]
5	1,2,4,5-tetrathiane	291-22-5	sulfury, burnt	[$^{2}H_4$]-1,2,4,5-tetrathiane	0.23 µg/kg in water	12	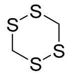	Raw Shiitake	[174]
	1,2,4,5-tetrathiane	291-22-5	sulfury, burnt	[$^{2}H_4$]-1,2,4,5-tetrathiane	0.23 µg/kg in water	891	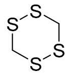	Pan-Fried Shiitake Mushrooms	[174]
6	1,2,4,6-tetrathiepane	292-45-5	sulfury, burnt	[$^{2}H_6$]-1,2,4,6-tetrathiepane	1.16 µg/kg in water	9	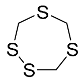	Raw Shiitake	[174]
	1,2,4,6-tetrathiepane	292-45-5	sulfury, burnt	[$^{2}H_6$]-1,2,4,6-tetrathiepane	1.16 µg/kg in water	2	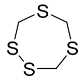	Pan-Fried Shiitake Mushrooms	[174]
7	1,2,3,5,6-pentathiepane	292-46-6	sulfury, burnt	[$^{2}H_4$]-1,2,3,5,6-pentathiepane	0.27 µg/kg in water	10	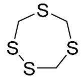	Raw Shiitake	[174]
	1,2,3,5,6-pentathiepane	292-46-6	sulfury, burnt	[$^{2}H_4$]-1,2,3,5,6-pentathiepane	0.27 µg/kg in water	981	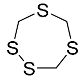	Pan-Fried Shiitake Mushrooms	[174]
8	methionol	505-10-2	potato	[$^{2}H_3$]-methionol	250 µg/kg in water	2.73	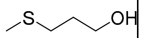	Thai Fish Sauce A	[132]

Continued

No.	Compound	CAS No.	Odor	Labeled Standards	OT	OAV	Structure	Foods	Refs.
8	methionol	505-10-2	potato	$[^{2}H_{3}]$-methionol	250 μg/kg in water	2.36	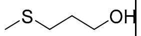	Thai Fish Sauce B	[132]
	methionol	505-10-2	cooked potato-like, tallowy	$[^{2}H_{3}]$-methionol	36μg/kg in water	1	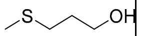	Italian Tomatoes	[175]
9	1-isothiocyanato-3-(methylsulfanyl)propane	505-79-3	sulfurous, garlic-like, mushroom-like	$[^{2}H_{8}]$-naphthalene[b]	5.0 μg/kg in water	270	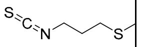	Raw Green Kohlrabi	[176]
	1-isothiocyanato-3-(methylsulfanyl)propane	505-79-3	sulfurous, garlic-like, mushroom-like	$[^{2}H_{8}]$-naphthalene[b]	5.0 μg/kg in water	270	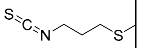	Cooked Green Kohlrabi	[176]
10	benzyl isothiocyanate	622-78-6	herb-like, sweaty, pungent	$[^{2}H_{8}]$-naphthalene[b]	0.70 μg/kg in water	220	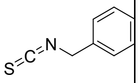	Raw Green Kohlrabi	[176]
	benzyl isothiocyanate	622-78-6	herb-like, sweaty, pungent	$[^{2}H_{8}]$-naphthalene[b]	0.70 μg/kg in water	190	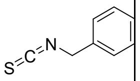	Cooked Green Kohlrabi	[176]
11	dimethyl disulfide	624-92-0	sulphuric, onion	$[^{2}H_{6}]$-dimethyl disulfide	0.2 μg/kg in water	384	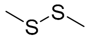	4 days of ripening cottage cheese	[164]
	dimethyl disulfide	624-92-0	sulphuric, onion	$[^{2}H_{6}]$-dimethyl disulfide	0.2 μg/kg in water	10	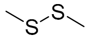	Fried cottage cheese	[164]

Continued

No.	Compound	CAS No.	Odor	Labeled Standards	OT	OAV	Structure	Foods	Refs.
12	bis(methylthio)methane	1618-26-4	sulfury, garlic-like	[$^2H_8$]-bis(methylthio)methane	0.012 µg/kg in water	817000	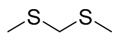	White Alba Truffle	[160]
13	(2-isothiocyanatoethyl) benzene	2257-09-2	broth-like, vegetable-like	[$^2H_8$]-naphthalene^b	6.0 µg/kg in water	7	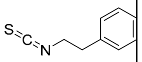	Raw Green Kohlrabi	[176]
	(2-isothiocyanatoethyl) benzene	2257-09-2	broth-like, vegetable-like	[$^2H_8$]-naphthalene^b	6.0 µg/kg in water	7	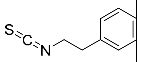	Cooked Green Kohlrabi	[176]
14	benzyl thiocyanate	3012-37-1	broth-like, buttery, cress-like	[$^2H_8$]-naphthalene^b	5.0 µg/kg in water	4	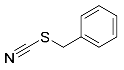	Raw Green Kohlrabi	[176]
15	methional	3268-49-3	boiled potato	[$^2H_5$]-methional	0.43 µg/kg in water	30	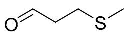	Sour Whey	[177]
	methional	3268-49-3	boiled potato	[$^2H_5$]-methional	0.43 µg/kg in water	26	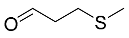	Buttermilk	[177]
	methional	3268-49-3	cooked potato	[$^2H_3$]-methional	0.43 µg/kg in water	1	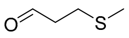	Dried Lobster Mushrooms	[178]
	methional	3268-49-3	cooked potato-like	[$^2H_3$]-methional	0.43 µg/kg in water	2	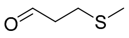	Raw Licorice (Glycyrrhiza glabra L.)	[179]
	methional	3268-49-3	cooked potato-like	[$^2H_3$]-methional	0.27 µg/kg in starch	46	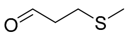	Crust of Soft Pretzels	[180]
	methional	3268-49-3	baked potato	[$^2H_3$]-methional	0.27 µg/kg in starch	59	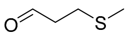	Bagels (CB)	[181]

Continued

No.	Compound	CAS No.	Odor	Labeled Standards	OT	OAV	Structure	Foods	Refs.
15	methional	3268-49-3	baked potato	$[^2H_3]$-methional	0.27 µg/kg in starch	89	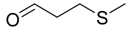	Bagels (BF_{24})	[181]
	methional	3268-49-3	baked potato	$[^2H_3]$-methional	0.27 µg/kg in starch	160	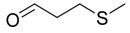	Bagels (BF_{48})	[181]
	methional	3268-49-3	cooked potato	$[^2H_3]$-methional	0.27 µg/kg in starch	56	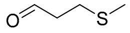	Yeast Dumplings	[182]
	methional	3268-49-3	cooked potato	$[^2H_3]$-methional	0.43 µg/kg in water	418	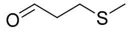	Chicken Egg	[130]
	methional	3268-49-3	cooked potato	$[^2H_3]$-methional	0.43 µg/kg in water	74	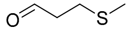	Plant-Based Egg	[130]
	methional	3268-49-3	cooked potato	$[^2H_3]$-methional	0.5 µg/kg in oil	26	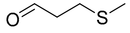	Raw rapeseeds	[131]
	methional	3268-49-3	cooked potato	$[^2H_3]$-methional	0.5 µg/kg in oil	54	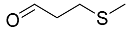	Rosasted rapeseeds	[131]
	methional	3268-49-3	boiled potato	$[^2H_3]$-methional	0.43 µg/kg in water	2047	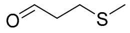	Pumpernickel Bread	[150]
	methional	3268-49-3	cooked potato	$[^2H_3]$-methional	0.2 µg/kg in water	1755	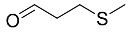	Thai Fish Sauce A	[132]
	methional	3268-49-3	cooked potato	$[^2H_3]$-methional	0.2 µg/kg in water	5460	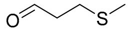	Thai Fish Sauce B	[132]
	methional	3268-49-3	cooked potato	$[^2H_3]$-methional	1.4 µg/L in water	121	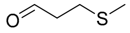	Lippia dulcis	[183]

Cotinued

No.	Compound	CAS No.	Odor	Labeled Standards	OT	OAV	Structure	Foods	Refs.
15	methional	3268-49-3	potato	[$^2H_3$]-methional	0.43 µg/kg in water	2	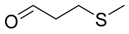	Daldinia childiae	[184]
	methional	3268-49-3	cooked potato-like	[$^2H_3$]-methional	0.27 µg/kg in starch	278	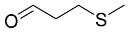	Sourdough Bread Crumb	[185]
	methional	3268-49-3	cooked potato	[$^2H_3$]-methional	0.52 µg/L in wine matrix (12% ethanol, 5 g/L tartaric acid, pH 3.5)	5	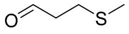	Australian Cabernet Sauvignon Wine	[186]
	methional	3268-49-3	cooked-potato-like	[$^2H_3$]-methional	0.27 µg/kg in starch	370	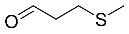	Freshly Prepared Oat Pastry	[133]
	methional	3268-49-3	cooked potato-like	[$^2H_3$]-methional	0.43 µg/kg in water	80	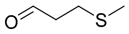	Raw Shiitake	[174]
	methional	3268-49-3	cooked potato-like	[$^2H_3$]-methional	0.43 µg/kg in water	202	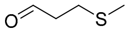	Pan-Fried Shiitake Mushrooms	[174]
	methional	3268-49-3	boiled potatoes	[$^2H_3$]-methional	0.2 µg/kg in water	601	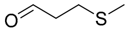	Roasted Goose	[45]
	methional	3268-49-3	cooked potato	[$^2H_3$]-methional	0.18 µg/kg in oil	13	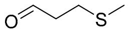	Raw Hazelnuts A	[165]
	methional	3268-49-3	cooked potato	[$^2H_3$]-methional	0.18 µg/kg in oil	45	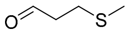	Roasted Hazelnut Paste	[165]

Continued

No.	Compound	CAS No.	Odor	Labeled Standards	OT	OAV	Structure	Foods	Refs.
15	methional	3268-49-3	boiled potato	[$^{2}H_{3}$]-methional	0.2 μg/L in water	150	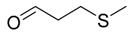	Fermented one day Tempeh	[153]
	methional	3268-49-3	boiled potato	[$^{2}H_{3}$]-methional	0.2 μg/L in water	820	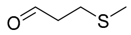	Fried Fermented one day Tempeh	[153]
	methional	3268-49-3	boiled potato	[$^{2}H_{3}$]-methional	0.2 μg/L in water	120	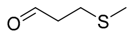	Fermented five days Tempeh	[153]
	methional	3268-49-3	boiled potato	[$^{2}H_{3}$]-methional	0.2 μg/L in water	900	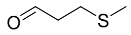	Fried Fermented five days Tempeh	[153]
	methional	3268-49-3	potato	[$^{2}H_{3}$]-methional	0.43 μg/kg in water	91	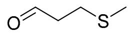	Tongtapan Thai chempedak	[45]
	methional	3268-49-3	potato	[$^{2}H_{3}$]-methional	0.43 μg/kg in water	91	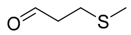	Sainumphung Thai chempedak	[45]
	methional	3268-49-3	cooked-potato	[$^{2}H_{3}$]-methional	0.18 μg/kg in oil	8	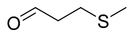	Raw Hazelnuts B	[166]
	methional	3268-49-3	cooked-potato	[$^{2}H_{3}$]-methional	0.18 μg/kg in oil	442	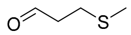	Roasted Hazelnuts	[166]
	methional	3268-49-3	potato-like	[$^{2}H_{3}$]-methional	250 μg/kg in water	4	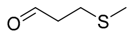	Bavarian Pilsner-type Beer	[155]
	methional	3268-49-3	cooked potatoes	[$^{2}H_{3}$]-methional	0.43 μg/kg in water	2.8	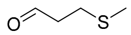	Smoked Oscypek	[187]
	methional	3268-49-3	cooked potatoes	[$^{2}H_{3}$]-methional	0.43 μg/kg in water	1.39	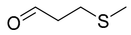	Unsmoked Oscypek	[187]
	methional	3268-49-3	cooked potato-like	[$^{2}H_{3}$]-methional	0.2 μg/kg in sunflower oil	109	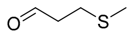	Pumpkin Seed Oil A	[134]

Continued

No.	Compound	CAS No.	Odor	Labeled Standards	OT	OAV	Structure	Foods	Refs.
15	methional	3268-49-3	cooked potato-like	$[^{2}H_{3}]$-methional	0.2 µg/kg in sunflower oil	71	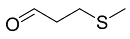	Pumpkin Seed Oil B	[134]
	methional	3268-49-3	cooked potato-like	$[^{2}H_{3}]$-methional	0.2 µg/kg in sunflower oil	75	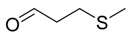	Pumpkin Seed Oil C	[134]
	methional	3268-49-3	cooked potato-like	$[^{2}H_{3}]$-methional	0.2 µg/kg in sunflower oil	34	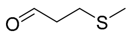	Pumpkin Seed Oil D	[134]
	methional	3268-49-3	cooked potato-like	$[^{2}H_{3}]$-methional	0.2 µg/kg in oil	1270	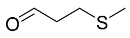	Gruyère cheeses with a potato-like off-flavour	[135]
	methional	3268-49-3	cooked potato-like	$[^{2}H_{3}]$-methional	0.2 µg/kg in oil	495	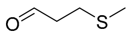	Gruyère cheeses without an off-flavour	[135]
	methional	3268-49-3	cooked potato	$[^{2}H_{3}]$-methional	1.4 µg/L in water	245	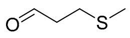	Soy Sauce	[188]
	methional	3268-49-3	cooked potato	$[^{2}H_{3}]$-methional	0.43 µg/kg in water	3.1	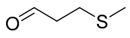	Pink Guava	[189]
	methional	3268-49-3	boiled potato	$[^{2}H_{3}]$-methional	0.27 µg/kg in Starch	278	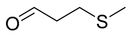	Crumb	[190]
	methional	3268-49-3	cooked potato	$[^{2}H_{3}]$-methional	0.27 µg/kg in Starch	896	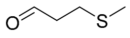	Rye Flour	[191]
	methional	3268-49-3	cooked potato	$[^{2}H_{3}]$-methional	1.8 µg/kg in water	241	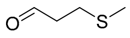	Rye Sourdough	[191]

Continued

No.	Compound	CAS No.	Odor	Labeled Standards	OT	OAV	Structure	Foods	Refs.
15	methional	3268-49-3	cooked potato	[$^{2}H_{3}$]-methional	0.2 µg/kg in water	500	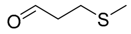	Robusta roasted coffee	[169]
	methional	3268-49-3	sulfurous, garlic-like	[$^{2}H_{3}$]-methional	0.2 µg/kg in sunflower oil were nasally estimated	625	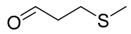	Camembert Cheese Samples I	[136]
	methional	3268-49-3	sulfurous, garlic-like	[$^{2}H_{3}$]-methional	0.2 µg/kg in sunflower oil were nasally estimated	135	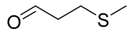	Camembert Cheese Samples II	[136]
	methional	3268-49-3	boiled potato	[^{2}H3]-methional	0.2 µg/kg in water	625	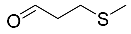	Dry spinach I	[137]
	methional	3268-49-3	boiled potato	[^{2}H3]-methional	0.2 µg/kg in water	90	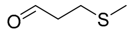	Dry spinach II	[137]
	methional	3268-49-3	cooked potato	[$^{2}H_{3}$]-methional	1.8 µg/kg in water	608	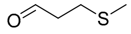	Thermally Treated Commercial Yeast Extract	[168]
	methional	3268-49-3	cooked potato	[$^{2}H_{3}$]-methional	1.8 µg/kg in water	159	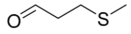	Thermally Treated Self-Prepared Extract of Bakers' Yeast	[168]
	methional	3268-49-3	cooked potato	[$^{2}H_{3}$]-methional	0.2 µg/kg in water	1200	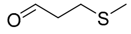	Arabica roasted coffee	[169]

Continued

No.	Compound	CAS No.	Odor	Labeled Standards	OT	OAV	Structure	Foods	Refs.
15	methional	3268-49-3	cooked potato	[$^2H_3$]-methional	0.2 µg/kg in water	500	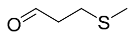	Robusta roasted coffee	[169]
	methional	3268-49-3	cooked potato	[$^2H_3$]-methional	0.04 µg/kg in water were retronasally estimated	5	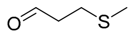	Hand-Squeezed Grapefruit Juice	[192]
	methional	3268-49-3	cooked potato	[$^2H_3$]-methional	0.04 µg/kg in water were retronasally estimated	10	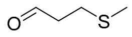	Hand-Squeezed Juices of Valencia Late Oranges	[193]
	methional	3268-49-3	cooked potato	[$^2H_3$]-methional	0.04 µg/kg in water were retronasally estimated	8	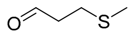	Hand-Squeezed Juices of Navel Oranges	[193]
	methional	3268-49-3	cooked potato	[$^2H_3$]-methional	0.2 µg/kg in sunflower oil	43	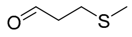	Raw Peanuts	[138]
	methional	3268-49-3	cooked potato	[$^2H_3$]-methional	0.2 µg/kg in sunflower oil	200	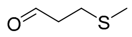	Pan-Roasted Peanut Meal	[138]
	methional	3268-49-3	boiled potato	[$^2H_3$]-methional	0.27 µg/kg in starch	99	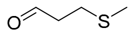	Baguette intensifie´e	[194]
	methional	3268-49-3	boiled potato	[$^2H_3$]-methional	0.27 µg/kg in starch	159	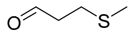	Baguette artisanale	[194]

Continued

No.	Compound	CAS No.	Odor	Labeled Standards	OT	OAV	Structure	Foods	Refs.
	methional	3268-49-3	boiled potato	[$^2H_3$]-methional	500 µg/L in water/ethanol (90 + 10, w/w)	2	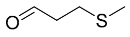	Scheurebe White Wine	[156]
	methional	3268-49-3	boiled potato	[$^2H_3$]-methional	500 µg/L in water/ethanol (90 + 10, w/w)	3	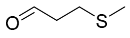	Gewürztraminer White Wine	[156]
	methional	3268-49-3	boiledpotato	[$^2H_3$]-methional	0.04 µg/kg in water	145	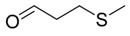	Boiled Trout A	[195]
	methional	3268-49-3	boiledpotato	[$^2H_3$]-methional	0.04 µg/kg in water	188	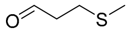	Boiled Trout B	[195]
	methional	3268-49-3	boiledpotato	[$^2H_3$]-methional	0.04 µg/kg in water	100	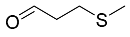	Boiled Trout Homogenates C	[195]
15	methional	3268-49-3	boiledpotato	[$^2H_3$]-methional	0.04 µg/kg in water	130	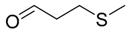	Boiled Trout Homogenates D	[195]
	methional	3268-49-3	Boiled potato	[$^2H_3$]-methional	0.27 µg/kg in starch	178	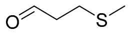	Toasting of Wheat Bread	[196]
	methional	3268-49-3	cooked potato-like	[$^2H_3$]-methional	0.2 µg/kg in oil	740	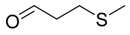	Hungarian-Type Salami	[170]
	methional	3268-49-3	boiled potato-like	[$^2H_3$]-methional	0.2 µg/kg in oil	75	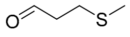	Raw Salmon Homogenates	[139]
	methional	3268-49-3	boiled potato-like	[$^2H_3$]-methional	0.2 µg/kg in oil	200	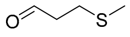	Boiled Salmon Homogenates	[139]
	methional	3268-49-3	boiled potato-like	[$^2H_3$]-methional	0.2 µg/kg in oil	110	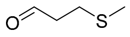	Boiled Salmon Homogenates B	[139]

Continued

No.	Compound	CAS No.	Odor	Labeled Standards	OT	OAV	Structure	Foods	Refs.
15	methional	3268-49-3	boiled potato-like	[$^2H_3$]-methional	0.2 μg/kg in oil	275	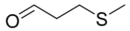	Boiled Cod Homogenates C	[139]
	methional	3268-49-3	boiled potato-like	[$^2H_3$]-methional	0.2 μg/kg in oil	250	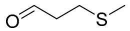	Boiled Cod Homogenates D	[139]
	methional	3268-49-3	boiled potato	[$^2H_3$]-methional	0.2 μg/kg in water	165	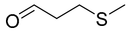	Cooked meat patties A	[197]
	methional	3268-49-3	boiled potato	[$^2H_3$]-methional	0.2 μg/kg in water	680	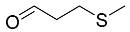	Cooked meat patties B	[197]
	methional	3268-49-3	boiled potato	[$^2H_3$]-methional	0.2 μg/kg in water	2	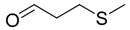	Elstar appples	[198]
	methional	3268-49-3	boiled potato	[$^2H_3$]-methional	0.2 μg/kg in water	6	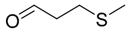	Cox orange apples	[198]
	methional	3268-49-3	boiled potato	[$^2H_3$]-methional	0.2 μg/kg in water	325	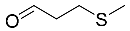	Dry parsley I	[141]
	methional	3268-49-3	boiled potato	[$^2H_3$]-methional	0.2 μg/kg in water	100	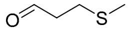	Dry parsley V	[141]
	methional	3268-49-3	boiled potato	[$^2H_3$]-methional	0.2 μg/kg in water	29	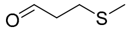	Ground Roasted Arabica Coffee	[142]
	methional	3268-49-3	boiled potato	[$^2H_3$]-methional	0.2 μg/kg in water	14	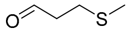	Ground Roasted Robusta Coffee	[142]
	methional	3268-49-3	boiled-potato-like	[$^2H_3$]-methional	0.2 μg/kg in water	75	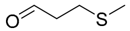	Cooked chicken A	[143]
	methional	3268-49-3	boiled-potato-like	[$^2H_3$]-methional	0.2 μg/kg in water	175	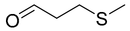	Cooked chicken B	[143]

Continued

No.	Compound	CAS No.	Odor	Labeled Standards	OT	OAV	Structure	Foods	Refs.
15	methional	3268-49-3	cooked potato-like	[$^2H_3$]-methional	0.2 μg/kg in water	16	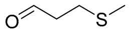	Dornfelder Red Wine	[199]
	methional	3268-49-3	boiled-potato-like	[$^2H_3$]-methional	0.05 μg/kg in sunflower oil retronasally estimated	660	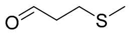	Emmentaler Cheese A	[200]
	methional	3268-49-3	boiled-potato-like	[$^2H_3$]-methional	0.05 μg/kg in sunflower oil retronasally estimated	306	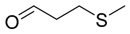	Emmentaler Cheese B	[200]
	methional	3268-49-3	boiled potato-like	[$^2H_3$]-methional	0.2 μg/kg in water	55	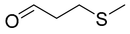	Fresh boiled cod	[144]
	methional	3268-49-3	boiled potato-like	[$^2H_3$]-methional	0.2 μg/kg in water	6	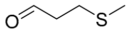	Stored boiled cod	[144]
	methional	3268-49-3	cooked potato	[$^2H_3$]-methional	0.43 μg/kg in water	190	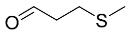	Roasted Ginger	[201]
	methional	3268-49-3	cooked potato	[$^2H_3$]-methional	1.4 μg/kg in water	51,700	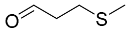	Lupin protein isolate fermented with Lupin protein isolate	[202]
	methional	3268-49-3	cooked potato	[$^2H_3$]-methional	1.4 μg/kg in water	2	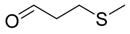	Lupin protein isolate fermented with Staphylococcus xylosus	[202]

Continued

No.	Compound	CAS No.	Odor	Labeled Standards	OT	OAV	Structure	Foods	Refs.
15	methional	3268-49-3	cooked potato	$[^2H_3]$-methional	1.4 µg/kg in water	2	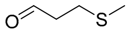	Lupin protein isolate fermented with Lactobacillus sakei ssp. carnosus	[202]
	methional	3268-49-3	cooked potato-like	$[^2H_3]$-methional	0.43µg/kg in water	27	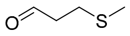	Italian Tomatoes	[175]
	methional	3268-49-3	cooked potato-like, tallowy	$[^2H_3]$-methional	0.43µg/kg in water	24	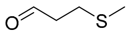	Fried Prawn Meat	[145]
	methional	3268-49-3	cooked potato-like, tallowy	$[^2H_3]$-methional	0.43µg/kg in water	24	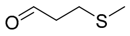	Blanched Prawn Meat	[145]
	methional	3268-49-3	cooked potato-like, tallowy	$[^2H_3]$-methional	0.43µg/kg in water	14	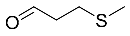	Carmenere Red Wines CMR 1	[172]
	methional	3268-49-3	cooked potato-like, tallowy	$[^2H_3]$-methional	0.43µg/kg in water	14	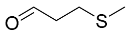	Carmenere Red Wines CMR 4	[172]
	methional	3268-49-3	cooked potato-like	$[^2H_3]$-methional	0.43 µg/kg in water	93	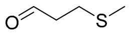	Amontillado Sherry Wine	[203]

Continued

No.	Compound	CAS No.	Odor	Labeled Standards	OT	OAV	Structure	Foods	Refs.
15	methional	3268-49-3	cooked potato-like, tallowy	$[^{2}H_{3}]$-methional	0.43 μg/kg in water	3.7	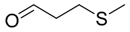	Chardonnay Marc Skins	[204]
	methional	3268-49-3	cooked potato-like, tallowy	$[^{2}H_{3}]$-methional	0.43μg/L in water/ethanol (9/1, v/v; orthonasal)	20	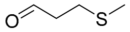	10-Year-Old Riesling Wine	[205]
	methional	3268-49-3	cooked potato-like, tallowy	$[^{2}H_{3}]$-methional	1.4μg/L in water	43	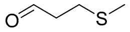	Stewed Beef Vegetable Gravy	[206]
	methional	3268-49-3	cooked potato-like, tallowy	$[^{2}H_{3}]$-methional	1.4μg/L in water	34	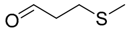	Stewed Pork Vegetable Gravy	[206]
	methional	3268-49-3	cooked potato-like, tallowy	$[^{2}H_{3}]$-methional	0.43 μg/kg in water	807	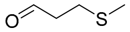	White Alba Truffle	[160]
	methional	3268-49-3	cooked potato-like, tallowy	$[^{2}H_{3}]$-methional	0.43 μg/kg in water	4	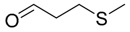	Burgundy Truffle	[160]
	methional	3268-49-3	cooked potato-like, tallowy	$[^{2}H_{3}]$-methional	0.2ug/kg in sunflower oil	1.5	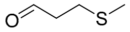	Peanut butter 1	[207]
	methional	3268-49-3	cooked potato-like	$[^{2}H_{3}]$-methional	0.2ug/kg in sunflower oil	3.1	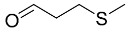	Peanut butter 2	[207]

Continued

No.	Compound	CAS No.	Odor	Labeled Standards	OT	OAV	Structure	Foods	Refs.
15	methional	3268-49-3	cooked potato-like, tallowy	[$^2H_3$]-methional	0.2ug/kg in sunflower oil	2.6	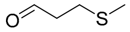	Peanut butter 3	[207]
	methional	3268-49-3	cooked potato-like, tallowy	[$^2H_3$]-methional	0.2ug/kg in sunflower oil	4.7	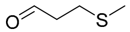	Peanut butter 4	[207]
	methional	3268-49-3	Baked potato	[$^2H_2$]-methional	0.27 μg/kg in starch	278	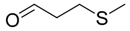	Fry breads UFBs	[208]
	methional	3268-49-3	Baked potato	[$^2H_2$]-methional	0.27 μg/kg in starch	267	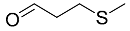	Fry breads FBs	[208]
	methional	3268-49-3	cooked potato-like, tallowy	--[a]	0.43 μg/kg in water	3	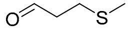	Enzymatic Hydrolysis of Mushroom Protein and Cysteine	[173]
	methional	3268-49-3	cooked potato-like, tallowy	--[a]	0.43 μg/kg in water	4	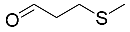	Enzymatic Hydrolysis of Mushroom Protein	[173]
	methional	3268-49-3	cooked potato	[$^2H_3$]-methional	0.2 μg/kg in water	31	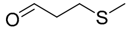	4 days of Ripening cottage cheese	[164]

Continued

No.	Compound	CAS No.	Odor	Labeled Standards	OT	OAV	Structure	Foods	Refs.
15	methional	3268-49-3	cooked potato	[$^2H_3$]-methional	0.2 µg/kg in water	36	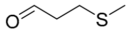	Fried cottage cheese	[164]
	methional	3268-49-3	cooked potato	[$^2H_3$]-methional	0.43 µg/kg in water	91	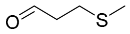	Thai chempedak fruit Tongtapan	[209]
	methional	3268-49-3	cooked potato	[$^2H_3$]-methional	0.43 µg/kg in water	91	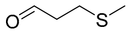	Thai chempedak fruit Sainumphung	[209]
16	diethyl trisulfide	3600-24-6	fried shallot	[$^2H_{10}$]-diethyl trisulfide	0.2 µg/kg in water	980	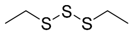	Monthong Durian Pulp	[147]
17	dimethyl trisulfide	3658-80-8	cabbage	[$^2H_6$]-dimethyl trisulfide	0.099 µg/kg in water	6	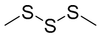	Sour Whey	[177]
	dimethyl trisulfide	3658-80-8	cabbage	[$^2H_6$]-dimethyl trisulfide	0.099 µg/kg in water	15	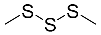	Buttermilk	[177]
	dimethyl trisulfide	3658-80-8	cabbage-like	[$^2H_6$]-dimethyl trisulfide	0.03 µg/kg in oil	37	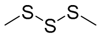	Rapeseed oil	[148]
	dimethyl trisulfide	3658-80-8	cabbage-like	[$^2H_6$]-dimethyl trisulfide	0.03 µg/kg in oil	178	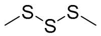	Milk Chocolate	[129]
	dimethyl trisulfide	3658-80-8	cabbage-like	[$^2H_6$]-dimethyl trisulfide	0.0099 µg/kg in water	1	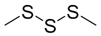	Raw Licorice (Glycyrrhiza glabra L.)	[179]
	dimethyl trisulfide	3658-80-8	cabbage-like	[$^2H_6$]-dimethyl trisulfide	0.03 µg/kg in oil	840	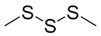	Dark Chocolates (90%CC)	[210]

Continued

No.	Compound	CAS No.	Odor	Labeled Standards	OT	OAV	Structure	Foods	Refs.
17	dimethyl trisulfide	3658-80-8	cabbage-like	[$^2H_6$]-dimethyl trisulfide	0.03 μg/kg in oil	730	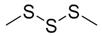	Dark Chocolates (99%CC)	[210]
	dimethyl trisulfide	3658-80-8	cabbage-like	[$^2H_6$]-dimethyl trisulfide	0.03 μg/kg in oil	44	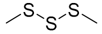	Raw rapeseeds	[131]
	dimethyl trisulfide	3658-80-8	cabbage-like	[$^2H_6$]-dimethyl trisulfide	0.03 μg/kg in oil	453	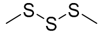	Rosasted rapeseeds	[131]
	dimethyl trisulfide	3658-80-8	cabbage	[$^2H_6$]-dimethyl trisulfide	0.0099 μg/kg in water	475	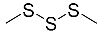	Pumpernickel Bread	[150]
	dimethyl trisulfide	3658-80-8	rotten, sulfurous, cabbage	[$^2H_6$]-dimethyl trisulfide	0.01 μg/kg in water	1751	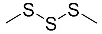	Thai Fish Sauce A	[132]
	dimethyl trisulfide	3658-80-8	rotten, sulfurous, cabbage	[$^2H_6$]-dimethyl trisulfide	0.01 μg/kg in water	2564	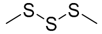	Thai Fish Sauce B	[132]
	dimethyl trisulfide	3658-80-8	rotten, sulfurous, cabbage	[$^2H_6$]-dimethyl trisulfide	2.5 μg/kg in oil	400	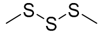	Dark Chocolates	[211]
	dimethyl trisulfide	3658-80-8	cabbage	[$^2H_6$]-dimethyl sulfide	0.0099 μg/kg in water	5	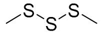	Mango Fruits	[212]
	dimethyl trisulfide	3658-80-8	cabbage-like	[$^2H_6$]-dimethyl trisulfide	0.03 μg/kg in oil	35	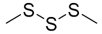	Steam-Treated Rapeseed Oil	[213]
	dimethyl trisulfide	3658-80-8	sulfurous, cabbage-like	[$^2H_6$]- dimethyl trisulfide	0.0099 μg/kg in water	370	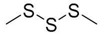	Raw Green Kohlrabi	[176]

Continued

No.	Compound	CAS No.	Odor	Labeled Standards	OT	OAV	Structure	Foods	Refs.
17	dimethyl trisulfide	3658-80-8	sulfurous, cabbage-like	[$^2H_6$]- dimethyl trisulfide	0.0099 µg/kg in water	220	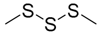	Cooked Green Kohlrabi	[176]
	dimethyl trisulfide	3658-80-8	cabbage-like	[$^2H_6$]-dimethyl trisulfide	0.086 µg/kg in starch	3	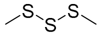	Freshly Prepared Oat Pastry	[133]
	dimethyl trisulfide	3658-80-8	sulfury, cabbage	[$^2H_6$]-Dimethyl trisulfide	0.099 µg/kg in water	100	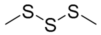	Pan-Fried Shiitake Mushrooms	[174]
	dimethyl trisulfide	3658-80-8	sulfurous, onion	--[a]	0.005 µg/kg in water	12	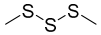	Roasted Goose	[45]
	dimethyl trisulfide	3658-80-8	sulfury, cabbage	[$^2H_6$]-dimethyl trisulfide	2.3 µg/kg in oil	164	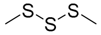	Roasted Hazelnut Paste	[165]
	dimethyl trisulfide	3658-80-8	cabbage	[$^2H_6$]-dimethyl trisulfide	0.01 µg/L in water	60	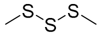	Fermented one day Tempeh	[153]
	dimethyl trisulfide	3658-80-8	cabbage	[$^2H_6$]-dimethyl trisulfide	0.01 µg/L in water	340	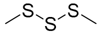	Fried Fermented one day Tempeh	[153]
	dimethyl trisulfide	3658-80-8	cabbage	[$^2H_6$]-dimethyl trisulfide	0.01 µg/L in water	680	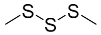	Fermented five days Tempeh	[153]
	dimethyl trisulfide	3658-80-8	cabbage	[$^2H_6$]-dimethyl trisulfide	0.01 µg/L in water	930	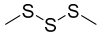	Fried Fermented five days Tempeh	[153]
	dimethyl trisulfide	3658-80-8	cabbage-like	[$^2H_6$]-dimethyl trisulfide	2.5 µg/kg in sunflower oil	8	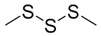	Pumpkin Seed Oil A	[134]

Continued

No.	Compound	CAS No.	Odor	Labeled Standards	OT	OAV	Structure	Foods	Refs.
17	dimethyl trisulfide	3658-80-8	cabbage-like	$[^2H_6]$-dimethyl trisulfide	0.03 μg/kg in an aqueous fructose-glucose solution	27	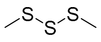	Rape Honey	[214]
	dimethyl trisulfide	3658-80-8	cabbage-like	$[^2H_6]$-dimethyl trisulfide	2.5 μg/kg in oil	27	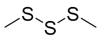	Gruyère cheeses with a potato-like off-flavour	[135]
	dimethyl trisulfide	3658-80-8	cabbage-like	$[^2H_6]$-dimethyl trisulfide	2.5 μg/kg in oil	54	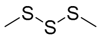	Gruyère cheeses without an off-flavour	[135]
	dimethyl trisulfide	3658-80-8	cabbage-like	$[^2H_6]$-dimethyl trisulfide	2.5 μg/kg in sunflower oil were nasally estimated	4	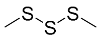	Camembert Cheese Samples I	[136]
	dimethyl trisulfide	3658-80-8	cabbage-like	$[^2H_6]$-dimethyl trisulfide	2.5 μg/kg in sunflower oil were nasally estimated	3	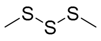	Camembert Cheese Samples II	[136]
	dimethyl trisulfide	3658-80-8	cabbage-like, sulphurous	$[^2H_6]$-dimethyl trisulfide	0.006 μg/kg in water	183	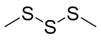	Dry spinach I	[137]
	dimethyl trisulfide	3658-80-8	cabbage-like, sulphurous	$[^2H_6]$-dimethyl trisulfide	0.006 μg/kg in water	117	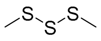	Dry spinach II	[137]

Continued

No.	Compound	CAS No.	Odor	Labeled Standards	OT	OAV	Structure	Foods	Refs.
17	dimethyl trisulfide	3658-80-8	sulfurous	[$^2H_6$]-dimethyl trisulfide	2.5 µg/kg in sunflower oil	3.6	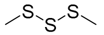	Unroasted beans	[215]
	dimethyl trisulfide	3658-80-8	sulfurous	[$^2H_6$]-dimethyl trisulfide	2.5 µg/kg in sunflower oil	21	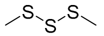	Roasted beans	[215]
	dimethyl trisulfide	3658-80-8	cabbage-like	[$^2H_6$]-dimethyl trisulfide	0.086 µg/kg in starch	22	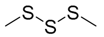	Baguette intensifie´e	[194]
	dimethyl trisulfide	3658-80-8	cabbage-like	[$^2H_6$]-dimethyl trisulfide	0.086 µg/kg in starch	27	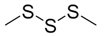	Baguette artisanale	[194]
	dimethyl trisulfide	3658-80-8	sulfury	[$^2H_6$]-dimethyl trisulfide	2.5 µg/kg in sunflower oil	2.8	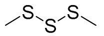	Cocoa Powder	[216]
	dimethyl trisulfide	3658-80-8	cabbage-like	[$^2H_6$]-dimethyl trisulfide	0.2 µg/L in water/ethanol (90 + 10, w/w)	1	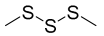	Gewürztraminer White Wine	[156]
	dimethyl trisulfide	3658-80-8	cabbage-like	[$^2H_6$]-dimethyl trisulfide	0.2 µg/L in water/ethanol (90 + 10, w/w)	1	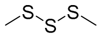	Gewürztraminer White Wine	[156]
	dimethyl trisulfide	3658-80-8	cabbage-like	[$^2H_3$]-dimethyl trisulfide	0.086 µg/kg in starch	52	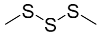	Toasting of Wheat Bread	[196]
	dimethyl trisulfide	3658-80-8	cabbage-like	[$^2H_3$]-dimethyl trisulfide	0.03 µg/kg in oil	19	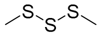	Boiled Cod Homogenates C	[139]

Continued

No.	Compound	CAS No.	Odor	Labeled Standards	OT	OAV	Structure	Foods	Refs.
17	dimethyl trisulfide	3658-80-8	cabbage-like	$[^{2}H_{3}]$-dimethyl trisulfide	0.03 µg/kg in oil	50	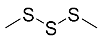	Boiled Cod Homogenates D	[139]
	dimethyl trisulfide	3658-80-8	sulphurous	$[^{2}H_{3}]$-dimethyl trisulfide	0.006 µg/kg in water	100	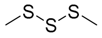	Dry parsley I	[141]
	dimethyl trisulfide	3658-80-8	sulphurous	$[^{2}H_{3}]$-dimethyl trisulfide	0.006 µg/kg in water	100	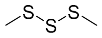	Dry parsley V	[141]
	dimethyl trisulfide	3658-80-8	cabbage-like	$[^{2}H_{6}]$-dimethyl trisulfide	0.006 µg/kg in water	113	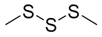	Cooked chicken A	[143]
	dimethyl trisulfide	3658-80-8	cabbage-like	$[^{2}H_{6}]$-dimethyl trisulfide	0.006 µg/kg in water	83	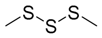	Cooked chicken B	[143]
	dimethyl trisulfide	3658-80-8	cabbage-like	$[^{2}H_{6}]$-dimethyl trisulfide	0.006 µg/kg in water	25	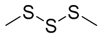	Fresh boiled cod	[144]
	dimethyl trisulfide	3658-80-8	cabbage-like	$[^{2}H_{6}]$-dimethyl trisulfide	0.006 µg/kg in water	50	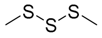	Stored boiled cod	[144]
	dimethyl trisulfide	3658-80-8	cabbage-like	$[^{2}H_{6}]$-dimethyl trisulfide	0.03 µg/kg in oil	1200	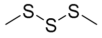	Hallertauer Mandarina Bavaria Hop Varieties	[217]
	dimethyl trisulfide	3658-80-8	cabbage-like	$[^{2}H_{6}]$-dimethyl trisulfide	0.03 µg/kg in oil	2400	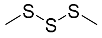	Hallertauer Cascade Hop Varieties	[217]

Continued

No.	Compound	CAS No.	Odor	Labeled Standards	OT	OAV	Structure	Foods	Refs.
17	dimethyl trisulfide	3658-80-8	cabbage-like	[$^2H_6$]-dimethyl trisulfide	0.03 µg/kg in oil	3200	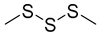	Hallertauer Mittelfrüh Hop Varieties	[217]
	dimethyl trisulfide	3658-80-8	cabbage-like	[$^2H_6$]-dimethyl trisulfide	0.03 µg/kg in oil	37	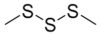	Native Cold-Pressed Rapeseed Oils PC	[159]
	dimethyl trisulfide	3658-80-8	cabbage-like	[$^2H_6$]-dimethyl trisulfide	0.03 µg/kg in oil	2900	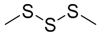	Native Cold-Pressed Rapeseed Oils OF	[159]
	dimethyl trisulfide	3658-80-8	cabbage-like	[$^2H_6$]-dimethyl trisulfide	0.03 µg/kg in oil	1900	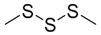	Rapeseeds OFS	[159]
	dimethyl trisulfide	3658-80-8	cabbage-like	[$^2H_6$]-dimethyl trisulfide	0.099 µg/kg in water	82	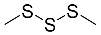	White Alba Truffle	[160]
	dimethyl trisulfide	3658-80-8	cabbage-like	[$^2H_6$]-dimethyl trisulfide	0.099 µg/kg in water	262	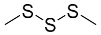	Burgundy Truffle	[160]
	dimethyl trisulfide	3658-80-8	sulfuric, cabbage-like	[$^2H_6$]-dimethyl trisulfide	2.5ug/kg in sunflower oil	12	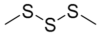	Peanut butter 1	[207]
	dimethyl trisulfide	3658-80-8	sulfuric, cabbage-like	[$^2H_6$]-dimethyl trisulfide	2.5ug/kg in sunflower oil	7.5	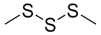	Peanut butter 2	[207]
	dimethyl trisulfide	3658-80-8	sulfuric, cabbage-like	[$^2H_6$]-dimethyl trisulfide	2.5ug/kg in sunflower oil	6.8	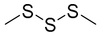	Peanut butter 3	[207]

Continued

No.	Compound	CAS No.	Odor	Labeled Standards	OT	OAV	Structure	Foods	Refs.
	dimethyl trisulfide	3658-80-8	sulfuric, cabbage-like	[$^2H_6$]-dimethyl trisulfide	2.5ug/kg in sunflower oil	2.2	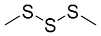	Peanut butter 4	[207]
17	dimethyl trisulfide	3658-80-8	sulfuric, cabbage-like	[$^2H_6$]-dimethyl trisulfide	0.01 µg/kg in water	244	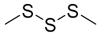	4 days of ripening cottage cheese	[164]
	dimethyl trisulfide	3658-80-8	sulfuric, cabbage-like	[$^2H_6$]-dimethyl trisulfide	0.01 µg/kg in water	10	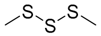	Fried cottage cheese	[164]
	2-phenylethylthiol	4410-99-5	rubbery, sulfurous	--[a]	0.05 µg/kg in water	33	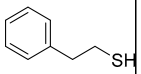	Roasted Goose	[45]
	2-phenylethylthiol	4410-99-5	burnt-rubbery	[^2H]-2-phenylethanethiol	0.05 µg/kg in sunflower oil	120	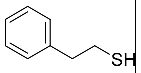	Roasted Sesame Seeds	[167]
	2-phenylethylthiol	4410-99-5	rubbery	[$^2H_2$]-2-phenylethylthiol	0.05 µg/kg in sunflower oil	360	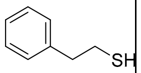	White sesame seeds (180℃)	[171]
	2-phenylethylthiol	4410-99-5	rubbery	[$^2H_2$]-2-phenylethylthiol	0.05 µg/kg in sunflower oil	2160	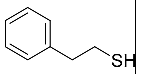	White sesame seeds (200℃)	[171]
18	2-phenylethylthiol	4410-99-5	Rubbery	[$^2H_2$]-2-phenylethylthiol	0.05 µg/kg in sunflower oil	8960	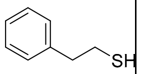	White sesame seeds (220℃)	[171]
	2-phenylethylthiol	4410-99-5	Rubbery	[$^2H_2$]-2-phenylethylthiol	0.05 µg/kg in sunflower oil	10800	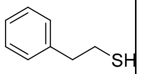	White sesame seeds (240℃)	[171]
	2-phenylethylthiol	4410-99-5	Rubbery	[$^2H_2$]-2-phenylethylthiol	0.05 µg/kg in sunflower oil	240	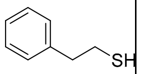	Black sesame seeds	[171]
	2-phenylethylthiol	4410-99-5	rubbery	[$^2H_2$]-2-phenylethylthiol	0.05 µg/kg in sunflower oil	880	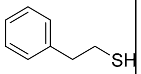	White sesame seeds	[171]

Continued

No.	Compound	CAS No.	Odor	Labeled Standards	OT	OAV	Structure	Foods	Refs.
19	1-isothiocyanato-4-(methylsulfanyl)butane	4430-36-8	mushroom-like, broth-like	[^{2}H$_{8}$]-naphthalene[b]	3.0 µg/kg in water	350	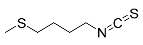	Raw Green Kohlrabi	[176]
	1-isothiocyanato-4-(methylsulfanyl)butane	4430-36-8	mushroom-like, broth-like	[^{2}H$_{8}$]-naphthalene[b]	3.0 µg/kg in water	350	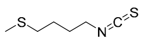	Cooked Green Kohlrabi	[176]
20	3-methylbut-2-ene-1-thiol	5287-45-6	sulfurous	[^{2}H$_{3}$]-3-methylbut-2-ene-1-thiol	0.00045 µg/L in water	120	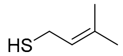	Mango Fruits	[212]
	3-methylbut-2-ene-1-thiol	5287-45-6	sulfury	[^{2}H$_{8}$]-3-methylbut-2-ene-1-thiol	0.0012 µg/kg in water	8	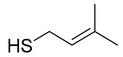	Bavarian Pilsner-type Beer	[155]
	3-methylbut-2-ene-1-thiol	5287-45-6	skunky	[^{2}H$_{8}$]-3-methylbut-2-ene-1-thiol	0.00045 µg/kg in water	27300	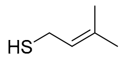	Ground Roasted Arabica Coffee	[142]
	3-methylbut-2-ene-1-thiol	5287-45-6	skunky	[^{2}H$_{8}$]-3-methylbut-2-ene-1-thiol	0.00045 µg/kg in water	27700	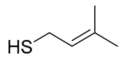	Ground Roasted Robusta Coffee	[142]
	3-methylbut-2-ene-1-thiol	5287-45-6	skunky	3-[^{2}H$_{3}$]-methyl-[4,4,4-^{2}H$_{3}$]-but-2-ene-1-thiol	0.00076 µg/kg in water	1200	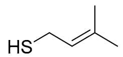	Monthong Durian Pulp	[147]
	3-methylbut-2-ene-1-thiol	5287-45-6	skunky	3-[^{2}H$_{3}$]-methyl-2-[4,4,4-^{2}H$_{3}$]-butene-1-thiol	0.00076 µg/kg in water	8276	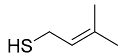	Hemp cultivars PG071	[218]

Continued

No.	Compound	CAS No.	Odor	Labeled Standards	OT	OAV	Structure	Foods	Refs.
20	3-methylbut-2-ene-1-thiol	5287-45-6	skunky	3-[$^2H_3$]-methyl-2-[4,4,4-$^2H_3$]-butene-1-thiol	0.00076 µg/kg in water	71000	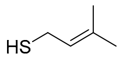	Hemp cultivars PG072	[218]
	3-methylbut-2-ene-1-thiol	5287-45-6	skunky	3-[$^2H_3$]-methyl-2-[4,4,4-$^2H_3$]-butene-1-thiol	0.00076 µg/kg in water	16816	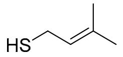	Hemp cultivars PG073	[218]
	3-methylbut-2-ene-1-thiol	5287-45-6	skunky	3-[$^2H_3$]-methyl-2-[4,4,4-$^2H_3$]-butene-1-thiol	0.00076 µg/kg in water	2421	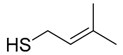	Hemp cultivars PG075.1	[218]
	3-methylbut-2-ene-1-thiol	5287-45-6	skunky	3-[$^2H_3$]-methyl-2-[4,4,4-$^2H_3$]-butene-1-thiol	0.00076 µg/kg in water	7260	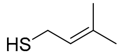	Hemp cultivars PG076	[218]
21	2-thenylthiol	6258-63-5	coffee-like	[^{2}H2]-2-thenylthiol	0.34 µg/kg in oil	27	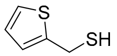	Roasted Hazelnut Paste	[165]
22	hydrogen sulfide	7783-06-4	boiled-egg like	--[a]	10 µg/kg in water	97	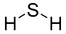	Chicken Egg	[130]
	hydrogen sulfide	7783-06-4	boiled-egg like	--[a]	10 µg/kg in water	50	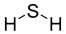	Plant-Based Egg	[130]
	hydrogen sulfide	7783-06-4	rotten egg	--[a]	10 µg/kg in water	330	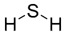	Monthong Durian Pulp	[147]
23	allylmethyl sulfide	10152-76-8	garlic-like	--[a]	22 µg/kg in oil	10	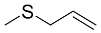	Hungarian-Type Salami	[170]
24	ethyl 3-(methylthio)propanoate	13327-56-5	meaty, onion, pineapple	[$^2H_9$]-ethyl 2-methylbutyrate[b]	7 µg/kg in water	5.586	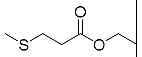	Australian pineapple (Aus Carnival)	[219]

Continued

No.	Compound	CAS No.	Odor	Labeled Standards	OT	OAV	Structure	Foods	Refs.
24	ethyl 3-(methylthio) propanoate	13327-56-5	meaty, onion, pineapple	[$^{2}H_{9}$]-ethyl 2-methylbutyrate[b]	7 μg/kg in water	5.630	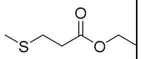	Australian pineapple (Aus Festival)	[219]
	ethyl 3-(methylthio) propanoate	13327-56-5	meaty, onion, pineapple	[$^{2}H_{9}$]-ethyl 2-methylbutyrate[b]	7 μg/kg in water	5.477	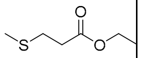	Australian pineapple (Aus Jubilee)	[219]
	ethyl 3-(methylthio) propanoate	13327-56-5	meaty, onion, pineapple	[$^{2}H_{9}$]-ethyl 2-methylbutyrate[b]	7 μg/kg in water	5.805	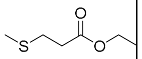	Australian pineapple (Aus Smooth)	[219]
	ethyl 3-(methylthio) propanoate	13327-56-5	meaty, onion, pineapple	[$^{2}H_{9}$]-ethyl 2-methylbutyrate[b]	7 μg/kg in water	6.518	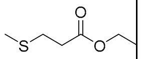	Australian pineapple (Aussie Gold)	[219]
25	methyl 3-(methylthio) propanoate	13532-18-8	meaty, onion-like	[$^{2}H_{9}$]-ethyl 2-methylbutyrate[b]	180 μg/kg in water	1.132	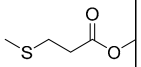	Australian pineapple (Aus Carnival)	[219]
	methyl 3-(methylthio) propanoate	13532-18-8	meaty, onion-like	[$^{2}H_{9}$]-ethyl 2-methylbutyrate[b]	180 μg/kg in water	3.315	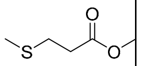	Australian pineapple (Aus Festival)	[219]
	methyl 3-(methylthio) propanoate	13532-18-8	meaty, onion-like	[$^{2}H_{9}$]-ethyl 2-methylbutyrate[b]	180 μg/kg in water	2.194	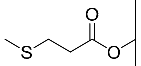	Australian pineapple (Aus Jubilee)	[219]

Continued

No.	Compound	CAS No.	Odor	Labeled Standards	OT	OAV	Structure	Foods	Refs.
25	methyl 3-(methylthio) propanoate	13532-18-8	meaty, onion-like	$[^{2}H_{9}]$-ethyl 2-methylbutyrate[b]	180 µg/kg in water	1.167	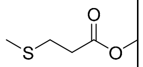	Australian pineapple (Aus Smooth)	[219]
26	2-mercapto-3-pentanone	17042-24-9	catty, black currantlike	$[^{2}H_{2}]$-3-mercapto-2-pentanone	0.6 µg/kg in sunflower oil	280	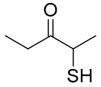	Pan-Roasted White Sesame Seeds	[126]
27	4-mercapto-4-methyl-2-pentanone	19872-52-7	black currant-like, sulphury	$[^{13}C_{4}]$-4-mercapto-4-methyl-2-pentanone	0.0016 µg/L in water	9	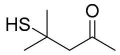	Longjing tea	[128]
	4-mercapto-4-methyl-2-pentanone	19872-52-7	tropical fruit	$[^{13}C_{4}]$-4-mercapto-4-methyl-2-pentanone	0.001 µg/L in water	51	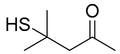	Mango Fruits	[212]
	4-mercapto-4-methyl-2-pentanone	19872-52-7	catty	$[^{2}H_{6}]$-3-mercapto-3-methylbutyl formiate	0.0001 µg/L in water	370000	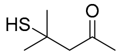	Lippia dulcis	[183]
	4-mercapto-4-methyl-2-pentanone	19872-52-7	black currant	4-$[^{13}C]$-methyl-4-sulfanyl-$[1,3,5-^{13}C_{3}]$-pentan-2-one	0.0001 µg/L in water	172	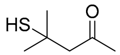	Special Flavor Hops (HN 2013)	[220]
	4-mercapto-4-methyl-2-pentanone	19872-52-7	black currant	4-$[^{13}C]$-methyl-4-sulfanyl-$[1,3,5-^{13}C_{3}]$pentan-2-one	0.0001 µg/L in water	1581	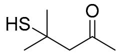	Special Flavor Hops (PA 2013)	[220]

Continued

No.	Compound	CAS No.	Odor	Labeled Standards	OT	OAV	Structure	Foods	Refs.
27	4-mercapto-4-methyl-2-pentanone	19872-52-7	black currant	4-[$^{2}H_{3}$]-mercapto-4-methyll-[1,1,1,3,5,5,5-$^{2}H_{7}$]-2-pentanone	0.0016 µg/kg in water	31119	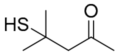	Hemp cultivars PG071	[218]
	4-mercapto-4-methyl-2-pentanone	19872-52-7	black currant	4-[$^{2}H_{3}$]-mercapto-4-methyll-[1,1,1,3,5,5,5-$^{2}H_{7}$]-2-pentanone	0.0016 µg/kg in water	6869	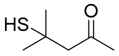	Hemp cultivars PG072	[218]
	4-mercapto-4-methyl-2-pentanone	19872-52-7	black currant	4-[$^{2}H_{3}$]-mercapto-4-methyll-[1,1,1,3,5,5,5-$^{2}H_{7}$]-2-pentanone	0.0016 µg/kg in water	9444	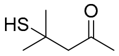	Hemp cultivars PG073	[218]
	4-mercapto-4-methyl-2-pentanone	19872-52-7	black currant	4-[$^{2}H_{3}$]-mercapto-4-methyll-[1,1,1,3,5,5,5-$^{2}H_{7}$]-2-pentanone	0.0016 µg/kg in water	3444	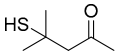	Hemp cultivars PG075.1	[218]
	4-mercapto-4-methyl-2-pentanone	19872-52-7	black currant	4-[$^{2}H_{3}$]-mercapto-4-methyll-[1,1,1,3,5,5,5-$^{2}H_{7}$]-2-pentanone	0.0016 µg/kg in water	2606	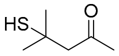	Hemp cultivars PG076	[218]
	4-mercapto-4-methyl-2-pentanone	19872-52-7	black currant	[13C$_{4}$]-4-mercapto-4-methyl-2-pentanone	0.0016 µg/kg in oil	660	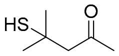	Hallertauer Mandarina Bavaria Hop Varieties	[217]
	4-mercapto-4-methyl-2-pentanone	19872-52-7	black currant	[13C$_{4}$]-4-mercapto-4-methyl-2-pentanone	0.0016 µg/kg in oil	380	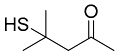	Hallertauer Cascade Hop Varieties	[217]

Continued

No.	Compound	CAS No.	Odor	Labeled Standards	OT	OAV	Structure	Foods	Refs.
27	4-mercapto-4-methyl-2-pentanone	19872-52-7	black currant	[13C$_4$]-4-mercapto-4-methyl-2-pentanone	0.0016 μg/kg in oil	19	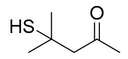	Hallertauer Mittelfrüh Hop Varieties	[217]
	4-mercapto-4-methyl-2-pentanone	19872-52-7	black currant	[13C$_4$]-4-mercapto-4-methyl-2-pentanone	0.0001 μg/L in water	7	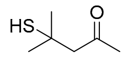	Low addition wines	[221]
	4-mercapto-4-methyl-2-pentanone	19872-52-7	black currant	[13C$_4$]-4-mercapto-4-methyl-2-pentanone	0.0001 μg/L in water	13.5	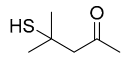	High addition wines	[221]
28	di-1-propenyl disulfide	23838-23-5	roasted onion-like	[$^2H_6$]-dipropyl disulfide	0.0034 μg/kg in water	970 000	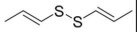	Freeze-dried green T. sinensis	[162]
	di-1-propenyl disulfide	23838-23-5	roasted onion-like	[$^2H_6$]-dipropyl disulfide	0.0034 μg/kg in water	53 000	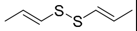	Solar-dried green T. sinensis	[162]
	di-1-propenyl disulfide	23838-23-5	roasted onion-like	[$^2H_6$]-dipropyl disulfide	0.0034 μg/kg in water	2200	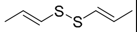	Oven-dried green T. sinensis	[162]
	di-1-propenyl disulfide	23838-23-5	roasted onion-like	[$^2H_6$]-dipropyl disulfide	0.0034 μg/kg in water	400 000	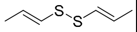	Freeze-dried red T. sinensis	[162]
	di-1-propenyl disulfide	23838-23-5	roasted onion-like	[$^2H_6$]-dipropyl disulfide	0.0034 μg/kg in water	29 000	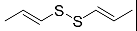	Solar-dried red T. sinensis	[162]
	di-1-propenyl disulfide	23838-23-5	roasted onion-like	[$^2H_6$]-dipropyl disulfide	0.0034 μg/kg in water	1100	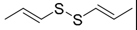	Oven-dried red T. sinensis	[162]
29	2-acetylthiazole	24295-03-2	roasty	[$^{13}C_2$]-2-acetylthiazole	48 μg/L in water/ethanol(6:4 by vol.)	3	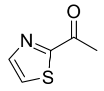	Tennessee Whiskey (bLCP)	[222]

Continued

No.	Compound	CAS No.	Odor	Labeled Standards	OT	OAV	Structure	Foods	Refs.
29	2-acetylthiazole	24295-03-2	roasty	$[^{13}C_2]$-2-acetylthiazole	48 μg/Lin water/ethanol (6:4 by vol.)	1	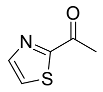	Tennessee Whiskey (aLCP)	[222]
	2-acetylthiazole	24295-03-2	roasted, popcorn	--[a]	3 μg/kg in water	1	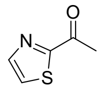	Roasted Goose	[45]
	2-acetylthiazole	24295-03-2	roasty	--[a]	10 μg/kg in water	1	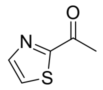	Enzymatic Hydrolysis of Mushroom Protein	[173]
30	methylene bis(methylsulfide)	24949-35-7	sulfurous	methylene bis($[^2H_3]$-methylsulfide)	30 μg/kg in sunflower oil were nasally estimated	12	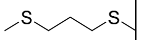	Camembert Cheese Samples I	[136]
	methylene bis(methylsulfide)	24949-35-7	sulfurous	methylene bis($[^2H_3]$-methylsulfide)	30 μg/kg in sunflower oil were nasally estimated	8	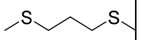	Camembert Cheese Samples II	[136]
31	ethane-1,1-dithiol	26914-40-9	sulfury, durian	$[^{13}C_2]$-ethane-1,1-dithiol	0.13 μg/kg in water	23000	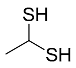	Monthong Durian Pulp	[147]
32	2-methyl-3-furanthiol	28588-74-1	meaty, thiamin	--[a]	0.007 μg/kg in water	397	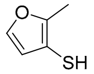	Roasted Goose	[45]
	2-methyl-3-furanthiol	28588-74-1	meaty	$[^2H_3]$-2-methyl-3-furanth iol	0.56 μg/kg in sunflower oil	180	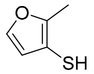	Pan-Roasted White Sesame Seeds	[126]

Continued

No.	Compound	CAS No.	Odor	Labeled Standards	OT	OAV	Structure	Foods	Refs.
32	2-methyl-3-furanthiol	28588-74-1	meat-like	[$^{2}H_{3}$]-2-methyl-3-furanthiol	0.007 μg/kg in water	75710	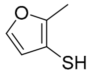	Thermally Treated Commercial Yeast Extract	[168]
	2-methyl-3-furanthiol	28588-74-1	meat-like	[$^{2}H_{3}$]-2-methyl-3-furanthiol	0.007 μg/kg in water	3140	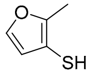	Thermally Treated Self-Prepared Extract of Bakers' Yeast	[168]
	2-methyl-3-furanthiol	28588-74-1	meat-like	[$^{2}H_{3}$]-2-methyl-3-furanthiol	0.007 μg/kg in water	75714	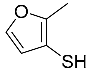	Commercial Yeast Extract(Glucose)	[168]
	2-methyl-3-furanthiol	28588-74-1	meat-like	[$^{2}H_{3}$]-2-methyl-3-furanthiol	0.007 μg/kg in water	22571	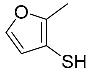	Commercial Yeast Extract(2-Oxopro panal)	[168]
	2-methyl-3-furanthiol	28588-74-1	meat-like	[$^{2}H_{2}$]-2-furfurylthiol[b]	0.56 μg/kg in oil	1	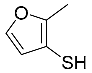	Hungarian-Type Salami	[170]
33	bis(2-methyl-3-furyl) disulfide	28588-75-2	meat-like	[$^{2}H_{6}$]-bis(2-methyl-3-furyl) disulfide	0.0008μg/L in water	39	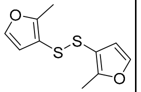	Stewed Beef Vegetable Gravy	[206]
	bis(2-methyl-3-furyl) disulfide	28588-75-2	Meat-like	[$^{2}H_{6}$]-bis(2-methyl-3-furyl) disulfide	0.0008μg/L in water	26	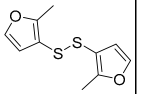	Stewed Pork Vegetable Gravy	[206]
34	2-acetyl-2-thiazoline	29926-41-8	roasty, popcorn-l ike	[$^{2}H_{4}$]-2-acetyl-2-thiazoline	2 μg/kg in starch	2	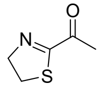	Freshly Prepared Oat Pastry	[133]

Continued

No.	Compound	CAS No.	Odor	Labeled Standards	OT	OAV	Structure	Foods	Refs.
34	2-acetyl-2-thiazoline	29926-41-8	roasted, popcorn	--[a]	0.12 μg/kg in water	153	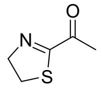	Roasted Goose	[45]
	2-acetyl-2-thiazoline	29926-41-8	roasty, popcorn	[$^2H_4$]-2-acetyl-2-thiazoline	1.0 μg/kg in water	3	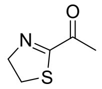	Thermally Treated Commercial Yeast Extract	[168]
	2-acetyl-2-thiazoline	29926-41-8	roasty, popcorn	[$^2H_4$]-2-acetyl-2-thiazoline	1.0 μg/kg in water	8	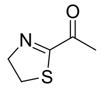	Thermally Treated Self-Prepared Extract of Bakers' Yeast	[168]
	2-acetyl-2-thiazoline	29926-41-8	roasty, popcorn	[$^2H_4$]-2-acetyl-2-thiazoline	1.0 μg/kg in water	13	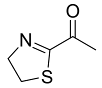	Thermal Treatment of Water-Soluble Low-MW Fractions from Bakers' Yeast A	[168]
	2-acetyl-2-thiazoline	29926-41-8	roasty, popcorn	[$^2H_4$]-2-acetyl-2-thiazoline	1.0 μg/kg in water	2	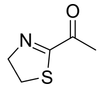	Thermal Treatment of Water-Soluble Low-MW Fractions from Bakers' Yeast B	[168]

Continued

No.	Compound	CAS No.	Odor	Labeled Standards	OT	OAV	Structure	Foods	Refs.
34	2-acetyl-2-thiazoline	29926-41-8	roasty, popcorn-like	[$^{2}H_4$]-2-acetyl-2-thiazoline	1.8 µg/kg in sunflower oil	1	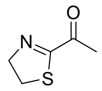	Pan-Roasted Peanut Meal	[138]
	2-acetyl-2-thiazoline	29926-41-8	roasty	[$^{2}H_4$]-2-acetyl-2-thiazoline	1.0 µg/kg in water	12	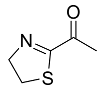	Cooked meat patties A	[197]
	2-acetyl-2-thiazoline	29926-41-8	roasty	[$^{2}H_4$]-2-acetyl-2-thiazoline	1.0 µg/kg in water	20	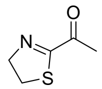	Cooked meat patties B	[197]
	2-acetyl-2-thiazoline	29926-41-8	sulphurousroasty	[$^{2}H_4$]-2-acetyl-2-thiazoline	1.0 µg/kg in water	14	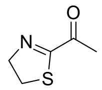	Cooked chicken A	[143]
	2-acetyl-2-thiazoline	29926-41-8	sulphurousroasty	[$^{2}H_4$]-2-acetyl-2-thiazoline	1.0 µg/kg in water	18	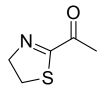	Cooked chicken B	[143]
	2-acetyl-2-thiazoline	29926-41-8	sulphurousroasty	[$^{2}H_4$]-2-acetyl-2-thiazoline	0.079	2	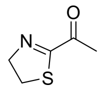	Fried Prawn Meat	[145]
35	1-(methylsulfanyl)ethane-1-thiol	31331-53-0	roasted onion	1-(ethylsulfanyl)-[$^{13}C_2$]ethane-1-thiol	0.038 µg/kg in water	4600	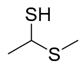	Monthong Durian Pulp	[147]
36	1-(ethylsulfanyl)ethane-1-thiol	31331-54-1	roasted onion	1-(methylsulfanyl)-[$^{13}C_2$]-ethane-1-thiol	0.0016 µg/kg in water	250000	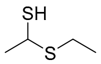	Monthong Durian Pulp	[147]

Continued

No.	Compound	CAS No.	Odor	Labeled Standards	OT	OAV	Structure	Foods	Refs.
37	3-sulfanyl-1-hexanol	51755-83-0	sulphury, grapefruit-like	[1,1-$^{2}H_{2}$]-3-sulfanyl-1-hexanol	0.06 µg/kg in water	270	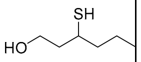	Green white Colombian guava	[223]
	3-sulfanyl-1-hexanol	51755-83-0	sulphury, grapefruit-like	[1,1-$^{2}H_{2}$]-3-sulfanyl-1-hexanol	0.06 µg/kg in water	130	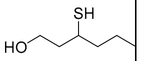	Mature white Colombian guava	[223]
	3-sulfanyl-1-hexanol	51755-83-0	sulphury, grapefruit-like	[1,1-$^{2}H_{2}$]-3-sulfanyl-1-hexanol	0.06 µg/kg in water	30	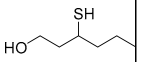	Overripe white Colombian guava	[223]
	3-sulfanyl-1-hexanol	51755-83-0	sulphury, grapefruit-like	[1,1-$^{2}H_{2}$]-3-sulfanyl-1-hexanol	0.06 µg/kg in water	40	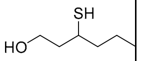	Overripe pink Colombian guava	[223]
	3-sulfanyl-1-hexanol	51755-83-0	grapefruit	[1,1-$^{2}H_{2}$]-3-sulfanyl-1-hexanol	0.06 µg/kg in water	9300	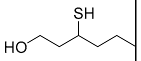	Pink Guava	[189]
	3-sulfanyl-1-hexanol	51755-83-0	grapefruit	3-sulfanyl-[5,5,6,6,6-$^{2}H_{5}$]-hexanol	0.02 µg/kg in water	3898	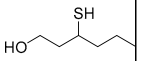	Hemp cultivars PG071	[218]
	3-sulfanyl-1-hexanol	51755-83-0	grapefruit	3-sulfanyl-[5,5,6,6,6-$^{2}H_{5}$]-hexan-1-ol	0.02 µg/kg in water	531	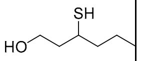	Hemp cultivars PG072	[218]
	3-sulfanyl-1-hexanol	51755-83-0	grapefruit	3-sulfanyl-[5,5,6,6,6-$^{2}H_{5}$]-hexan-1-ol	0.02 µg/kg in water	7890	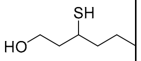	Hemp cultivars PG073	[218]
	3-sulfanyl-1-hexanol	51755-83-0	grapefruit	3-sulfanyl-[5,5,6,6,6-$^{2}H_{5}$]-hexan-1-ol	0.02 µg/kg in water	18991	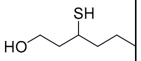	Hemp cultivars PG075.1	[218]
	3-sulfanyl-1-hexanol	51755-83-0	grapefruit	3-sulfanyl-[5,5,6,6,6-$^{2}H_{5}$]-hexan-1-ol	0.02 µg/kg in water	517	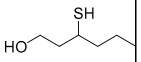	Hemp cultivars PG076	[218]

Continued

No.	Compound	CAS No.	Odor	Labeled Standards	OT	OAV	Structure	Foods	Refs.
37	3-sulfanyl-1-hexanol	51755-83-0	grapefruit	[$^2H_2$]-3-sulfanyl-1-hexanol	60 ng/L in a synthetic wine matrix solution	10	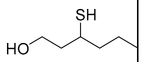	Carmenere Red Wines CMR 2	[172]
	3-sulfanyl-1-hexanol	51755-83-0	grapefruit	[$^2H_2$]-3-sulfanyl-1-hexanol	60 ng/L in a synthetic wine matrix solution	7	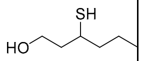	Carmenere Red Wines CMR 2	[172]
	3-sulfanyl-1-hexanol	51755-83-0	grapefruit	[$^2H_2$]-3-sulfanyl-1-hexanol	60 ng/L in a synthetic wine matrix solution	13	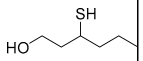	Carmenere Red Wines CMR 3	[172]
	3-sulfanyl-1-hexanol	51755-83-0	grapefruit	[$^2H_2$]-3-sulfanyl-1-hexanol	60 ng/L in a synthetic wine matrix solution	13	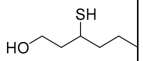	Carmenere Red Wines CMR 4	[172]
	3-sulfanyl-1-hexanol	51755-83-0	grapefruit	[$^2H_2$]-3-sulfanyl-1-hexanol	0.02 µg/kg in water	26	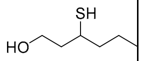	High addition wines	[221]
	3-sulfanyl-1-hexanol	51755-83-0	grapefruit	[$^2H_2$]-3-sulfanyl-1-hexanol	0.02 µg/kg in water	59	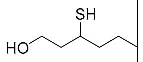	High addition wines	[221]
38	2-methyl-3-(methyldithio) furan	65505-17-1	cooked meat-like	[$^2H_3$]-2-methyl-3-(methyldithio) furan	0.37 µg/kg in oil	5	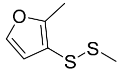	Dark Chocolates (90%CC)	[210]

Continued

No.	Compound	CAS No.	Odor	Labeled Standards	OT	OAV	Structure	Foods	Refs.
38	2-methyl-3-(methyldithio) furan	65505-17-1	cooked meat-like	[$^{2}H_3$]-2-methyl-3-(methyldithio) furan	0.37 µg/kg in oil	6	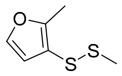	Dark Chocolates (99%CC)	[210]
	2-methyl-3-(methyldithio)furan	65505-17-1	pungent, sweaty	2-methyl-3-([$^{2}H_3$]-methyldithio)furan	0.006 µg/kg in water	15500	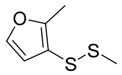	Vanilla Beans (MEX-D)	[224]
	2-methyl-3-(methyldithio)furan	65505-17-1	pungent, sweaty	2-methyl-3-([$^{2}H_3$]-methyldithio)furan	0.006 µg/kg in water	3200	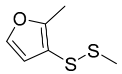	Vanilla Beans (CAM)	[224]
	2-methyl-3-(methyldithio)furan	65505-17-1	pungent, sweaty	2-methyl-3-([$^{2}H_3$]-methyldithio)furan	0.006 µg/kg in water	9200	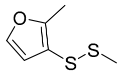	Vanilla Beans (TAH)	[224]
	2-methyl-3-(methyldithio)furan	65505-17-1	pungent, sweaty	2-methyl-3-([$^{2}H_3$]-methyldithio)furan	0.006 µg/kg in water	15400	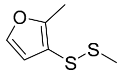	Vanilla Beans (MEX-A)	[224]
	2-methyl-3-(methyldithio)furan	65505-17-1	pungent, sweaty	2-methyl-3-([$^{2}H_3$]-methyldithio)furan	0.006 µg/kg in water	44300	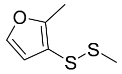	Vanilla Beans (MAD)	[224]
	2-methyl-3-(methyldithio)furan	65505-17-1	pungent, sweaty	2-methyl-3-([$^{2}H_3$]-methyldithio)furan	0.006 µg/kg in water	2690	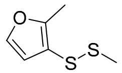	Vanilla Beans (UGA-K1)	[224]
	2-methyl-3-(methyldithio)furan	65505-17-1	pungent, sweaty	2-methyl-3-([$^{2}H_3$]-methyldithio)furan	0.006 µg/kg in water	2600	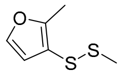	Vanilla Beans (UGA-K2)	[224]
	2-methyl-3-(methyldithio)furan	65505-17-1	pungent, sweaty	2-methyl-3-([$^{2}H_3$]-methyldithio)furan	0.006 µg/kg in water	14800	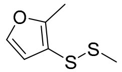	Vanilla Beans (SAP)	[224]
	2-methyl-3-(methyldithio)furan	65505-17-1	pungent, sweaty	2-methyl-3-([$^{2}H_3$]-methyldithio)furan	0.006 µg/kg in water	2840	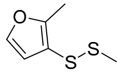	Vanilla Beans (IND-A)	[224]

Continued

No.	Compound	CAS No.	Odor	Labeled Standards	OT	OAV	Structure	Foods	Refs.
38	2-methyl-3-(methyldithio)furan	65505-17-1	meat-like, cabbage-like	[$^2H_8$]-naphthalene[b]	0.00026 µg/kg in water	27	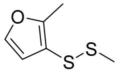	Raw Green Kohlrabi	[176]
	2-methyl-3-(methyldithio)furan	65505-17-1	meat-like, cabbage-like	[$^2H_8$]-naphthalene[b]	0.00026 µg/kg in water	6	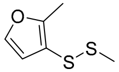	Cooked Green Kohlrabi	[176]
	2-methyl-3-(methyldithio)furan	65505-17-1	meat-like	[$^2H_3$]-2-methyl-3-(methyldithio)furan	0.4 µg/kg in sunflower oil	1.5	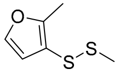	Roasted beans	[215]
	2-methyl-3-(methyldithio)furan	65505-17-1	cooked meat-like	[$^2H_3$]-2-methyl-3-(methyldithio)furan	0.4 µg/kg in sunflower oil	1.2	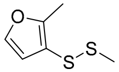	Cocoa Powder	[216]
	2-methyl-3-(methyldithio)furan	65505-17-1	cooked meat-like	[$^2H_3$]-2-methyl-3-(methyldithio)furan	0.37ug/kg in sunflower oil	6	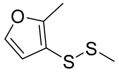	Peanut butter 1	[207]
	2-methyl-3-(methyldithio)furan	65505-17-1	cooked meat-like	[$^2H_3$]-2-methyl-3-(methyldithio)furan	0.37ug/kg in sunflower oil	3.2	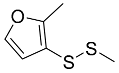	Peanut butter 2	[207]
	2-methyl-3-(methyldithio)furan	65505-17-1	cooked meat-like	[$^2H_3$]-2-methyl-3-(methyldithio)furan	0.37ug/kg in sunflower oil	4.3	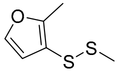	Peanut butter 3	[207]
	2-methyl-3-(methyldithio)furan	65505-17-1	cooked meat-like	[$^2H_3$]-2-methyl-3-(methyldithio)furan	0.37ug/kg in sunflower oil	1.9	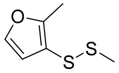	Peanut butter 4	[207]

Continued

No.	Compound	CAS No.	Odor	Labeled Standards	OT	OAV	Structure	Foods	Refs.
38	2-methyl-3-(methyldithio)furan	65505-17-1	meaty	--[a]	0.4 μg/kg in water	3	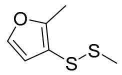	Enzymatic Hydrolysis of Mushroom Protein and Cysteine	[173]
39	3-mercapto-2-pentanone	67633-97-0	catty, black currantlike	$[^2H_2]$-3-mercapto-2-pentanone	0.19 μg/kg in sunflower oil	150	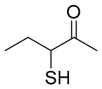	Pan-Roasted White Sesame Seeds	[126]
40	1-*p*-menthene-8-thiol	71159-90-5	grapefruit-like, sulfurous	$[^2H_6]$-1-*p*-menthene-8-thiol	0.0001 μg/kg in water were nasally estimated	100	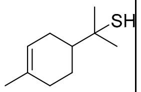	Hand-Squeezed Grapefruit Juice	[192]
41	propane-1-thiol	79869-58-2	rotten, durian	$[^2H_7]$-propane-1-thiol	0.49 μg/kg in water	6300	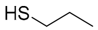	Monthong Durian Pulp	[147]
42	4-methoxy-2-methyl-2-butanethiol	94087-83-9	black currantlike,catty	$[^2H_3]$-4-methoxy-2-methyl-2-butanethiol	0.017 μg/kg in Refined Sunflower Oil were nasally estimated	253	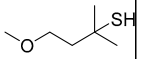	Spain Virgin Olive Oil	[225]

Continued

No.	Compound	CAS No.	Odor	Labeled Standards	OT	OAV	Structure	Foods	Refs.
42	4-methoxy-2-methyl-2-butanethiol	94087-83-9	black currantlike,catty	[$^2H_3$]-4-methoxy-2-methyl-2-butanethiol	0.024 μg/kg in Refined Sunflower Oil were retronasally estimated	179	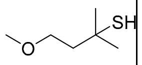	Spain Virgin Olive Oil	[225]
43	1-(2-furyl)ethanethiol	96631-04-8	meaty	--[a]	0.053 μg/kg in water	78	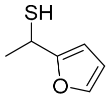	Enzymatic Hydrolysis of Mushroom Protein and Cysteine	[173]
44	di-1-propenyl trisulfide	115868-72-9	cooked onion-like	[$^2H_6$]-dipropyl trisulfide	0.26 μg/kg in water	7500	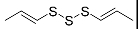	Freeze-dried green T. sinensis	[162]
	di-1-propenyl trisulfide	115868-72-9	cooked onion-like	[$^2H_6$]-dipropyl trisulfide	0.26 μg/kg in water	1600	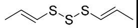	Solar-dried green T. sinensis	[162]
	di-1-propenyl trisulfide	115868-72-9	cooked onion-like	[$^2H_6$]-dipropyl trisulfide	0.26 μg/kg in water	66	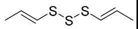	Oven-dried green T. sinensis	[162]
	di-1-propenyl trisulfide	115868-72-9	cooked onion-like	[$^2H_6$]-dipropyl trisulfide	0.26 μg/kg in water	2800	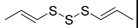	Freeze-dried red T. sinensis	[162]
	di-1-propenyl trisulfide	115868-72-9	cooked onion-like	[$^2H_6$]-dipropyl trisulfide	0.26 μg/kg in water	1300	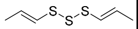	Solar-dried red T. sinensis	[162]
	di-1-propenyl trisulfide	115868-72-9	cooked onion-like	[$^2H_6$]-dipropyl trisulfide	0.26 μg/kg in water	94	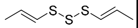	Oven-dried red T. sinensis	[162]

Continued

No.	Compound	CAS No.	Odor	Labeled Standards	OT	OAV	Structure	Foods	Refs.
45	3-sulfanylhexyl acetate	136954-20-6	sulphury, black currant-like	[1,1-$^{2}H_{2}$]-3-3-sulfanyl hexyl acetate	0.02 μg/kg in water	500	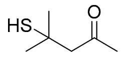	Green white Colombian guava	[223]
	3-sulfanylhexyl acetate	136954-20-6	sulphury, black currant-like	[1,1-$^{2}H_{2}$]-3-sulfanylhexyl acetate	0.02 μg/kg in water	460	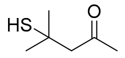	Mature white Colombian guava	[223]
	3-sulfanylhexyl acetate	136954-20-6	sulphury, black currant-like	[1,1-$^{2}H_{2}$]-3-sulfanylhexyl acetate	0.02 μg/kg in water	20	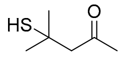	Overripe white Colombian guava	[223]
	3-sulfanylhexyl acetate	136954-20-6	sulphury, black currant-like	[1,1-$^{2}H_{2}$]-3-sulfanylhexyl acetate	0.02 μg/kg in water	100	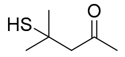	Overripe pink Colombian guava	[223]
	3-sulfanylhexyl acetate	136954-20-6	sulfurous, onion, strange	--[a]	0.02 μg/kg in water	54	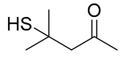	Roasted Goose	[45]
	3-sulfanylhexyl acetate	136954-20-6	black currant	[1,1-$^{2}H_{2}$]-3-sulfanylhexyl acetate	0.02 μg/kg in water	570	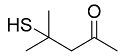	Pink Guava	[189]
	3-sulfanylhexyl acetate	136954-20-6	black currant	3-sulfanyl-[5,5,6,6,6-$^{2}H_{5}$]-hexan-1-ol[b]	0.06 μg/kg in water	1648	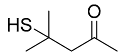	Hemp cultivars PG071	[218]
	3-sulfanylhexyl acetate	136954-20-6	black currant	3-sulfanyl-[5,5,6,6,6-$^{2}H_{5}$]-hexan-1-ol[b]	0.06 μg/kg in water	232	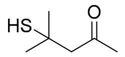	Hemp cultivars PG072	[218]
	3-sulfanylhexyl acetate	136954-20-6	black currant	3-sulfanyl-[5,5,6,6,6-$^{2}H_{5}$]-hexan-1-ol[b]	0.06 μg/kg in water	3026	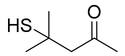	Hemp cultivars PG073	[218]
	3-sulfanylhexyl acetate	136954-20-6	black currant	3-sulfanyl-[5,5,6,6,6-$^{2}H_{5}$]-hexan-1-ol[b]	0.06 μg/kg in water	4513	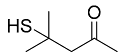	Hemp cultivars PG075.1	[218]
	3-sulfanylhexyl acetate	136954-20-6	black currant	3-sulfanyl-[5,5,6,6,6-$^{2}H_{5}$]-hexan-1-ol[b]	0.06 μg/kg in water	190	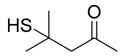	Hemp cultivars PG076	[218]

Continued

No.	Compound	CAS No.	Odor	Labeled Standards	OT	OAV	Structure	Foods	Refs.
45	3-sulfanylhexyl acetate	136954-20-6	box tree	$[^{2}H_{2}]$-3-sulfanylhexyl acetate	4.2 ng/L in a synthetic wine matrix solution	2	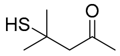	Carmenere Red Wines CMR 1	[172]
	3-sulfanylhexyl acetate	136954-20-6	box tree	$[^{2}H_{2}]$-3-sulfanylhexyl acetate	4.2 ng/L in a synthetic wine matrix solution	5	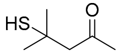	Carmenere Red Wines CMR 3	[172]
	3-sulfanylhexyl acetate	136954-20-6	box tree	$[^{2}H_{2}]$-3-sulfanylhexyl acetate	4.2 ng/L in a synthetic wine matrix solution	5	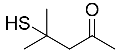	Carmenere Red Wines CMR 4	[172]
	3-sulfanylhexyl acetate	136954-20-6	box tree	$[^{2}H_{2}]$-3-sulfanylhexyl acetate	0.06 µg/kg in water	2	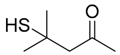	Low addition wines	[221]
	3-sulfanylhexyl acetate	136954-20-6	box tree	$[^{2}H_{2}]$-3-sulfanylhexyl acetate	0.06 µg/kg in water	7	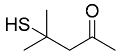	High addition wines	[221]

Continued

No.	Compound	CAS No.	Odor	Labeled Standards	OT	OAV	Structure	Foods	Refs.
46	2-mercapto-3,4-dimethyl -2,3-dihydrothiophene	137363-86-1	cooked onion-like/TS-like	[$^2H_6$]-dipropyl disulfide[b]	0.039 µg/kg in water	12 000	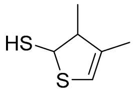	Freeze-dried green T. sinensis	[162]
	2-mercapto-3,4-dimethyl -2,3-dihydrothiophene	137363-86-1	cooked onion-like/TS-like	[$^2H_6$]-dipropyl disulfide[b]	0.039 µg/kg in water	9400	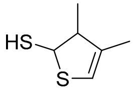	Solar-dried green T. sinensis	[162]
	2-mercapto-3,4-dimethyl -2,3-dihydrothiophene	137363-86-1	cooked onion-like/TS-like	[$^2H_6$]-dipropyl disulfide[b]	0.039 µg/kg in water	1400	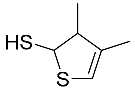	Oven-dried green T. sinensis	[162]
	2-mercapto-3,4-dimethyl -2,3-dihydrothiophene	137363-86-1	cooked onion-like/TS-like	[$^2H_6$]-dipropyl disulfide[b]	0.039 µg/kg in water	2600	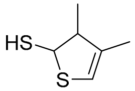	Freeze-dried red T. sinensis	[162]
	2-mercapto-3,4-dimethyl -2,3-dihydrothiophene	137363-86-1	cooked onion-like/TS-like	[$^2H_6$]-dipropyl disulfide[b]	0.039 µg/kg in water	4600	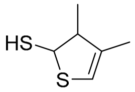	Solar-dried red T. sinensis	[162]
	2-mercapto-3,4-dimethyl -2,3-dihydrothiophene	137363-86-1	cooked onion-like	[$^2H_6$]-dipropyl disulfide	0.039 µg/kg in water	480	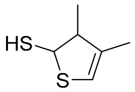	Oven-dried red T. sinensis	[162]
47	3-mercapto-2-methylpen tan-1-ol	227456-27-1	--[c]	[$^2H_2$]-3-mercapto-2-methylpentan-1-ol	0.0016µg/L in water	4800	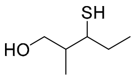	Stewed Beef Vegetable Gravy	[206]

Continued

No.	Compound	CAS No.	Odor	Labeled Standards	OT	OAV	Structure	Foods	Refs.
47	3-mercapto-2-methylpentan-1-ol	227456-27-1	--[c]	[$^2H_2$]-3-mercapto-2-methylpentan-1-ol	0.0016μg/L in water	4700	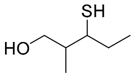	Stewed Pork Vegetable Gravy	[206]
48	2-methyl-3-sulfanyl-1-butanol	227456-33-9	sweet onion	[$^2H_2$]-2-methyl-3-sulfanyl-1-butanol	0.19 ng/L in a synthetic wine matrix solution	2700	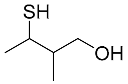	Carmenere Red Wines CMR 1	[172]
	2-methyl-3-sulfanyl-1-butanol	227456-33-9	sweet onion	[$^2H_2$]-2-methyl-3-sulfanyl-1-butanol	0.19 ng/L in a synthetic wine matrix solution	1500	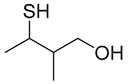	Carmenere Red Wines CMR 2	[172]
	2-methyl-3-sulfanyl-1-butanol	227456-33-9	sweet onion	[$^2H_2$]-2-methyl-3-sulfanyl-1-butanol	0.19 ng/L in a synthetic wine matrix solution	180	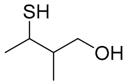	Carmenere Red Wines CMR 3	[172]
49	2-methyl-1-propene-1-thiol	--[d]	sulfurous, meaty	[$^2H_6$]-2-methyl-1-propene-1-thiol	30 μg/kg in sunflower oil	27	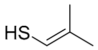	Pan-Roasted White Sesame Seeds	[126]
50	2-methyl-1-butene-1-thiol	--[d]	sulfurous, meaty	[$^2H_3$]-2-methyl-1-butene-1-thiol	1.25 μg/kg in sunflower oil	960	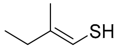	Pan-Roasted White Sesame Seeds	[126]

Continued

No.	Compound	CAS No.	Odor	Labeled Standards	OT	OAV	Structure	Foods	Refs.
51	1-(ethylsulfanyl)propane-1-thiol	--[d]	roasted onion	1-(ethylsulfanyl)-[2,2,3,3,3-$^{5}H_5$]propane-1-thiol	0.0063 μg/kg in water	1200	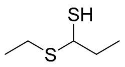	Monthong Durian Pulp	[147]
52	1-(ethyldisulfanyl)-1-(ethylsulfanyl)ethane	--[d]	sulfury, onion	1-(ethyldisulfanyl)-1-(ethylsulfanyl)-[$^{13}C_2$]-ethane	0.018 μg/kg in water	890	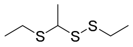	Monthong Durian Pulp	[147]
53	3-methyl-1-butene-1-thiol	1229617-33-7	sulfurous, meaty	[$^{2}H_3$]-2-methyl-1-butene-1-thiol	0.4 μg/kg in sunflower oil	2400	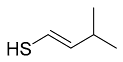	Pan-Roasted White Sesame Seeds	[126]
54	4-mercapto-3-hexanone	1229617-42-8	catty, black currantlike	[$^{2}H_3$]-4-mercapto-3-hexanone	0.07 μg/kg in sunflower oilg	65	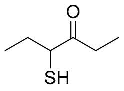	Pan-Roasted White Sesame Seeds	[126]

[a] SA, standard addition. [b] Quantification using stable isotope analogs.[c] Odor characteristics not described in the reference. [d] CAS number not found.

Note: Literature span: 1993–**2026**. A total of 54 key SACs, 221 food samples, and 101 references.

Table. S2 SACs with characteristic aromas

No.	Compound	Molecular structure	CAS No.	FEMA No.	Odor description	Refs.
1	2-methyl-3-furanthiol	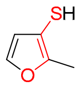	28588-74-1	3188	meat-like, sulfuric	[53]
2	thiophene-3-thiol	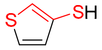	7774-73-4	-	cooked meat-like	[96]
3	2-methyl-3-(methyldithio)furan	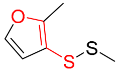	65505-17-1	3573	meat-like, cabbage-like	[7]
4	bis(2-methyl-3-furanyl)disulfane	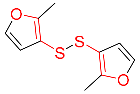	28588-75-2	3259	meat-like	[53]
5	2,3-butanedithiol	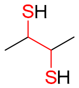	4532-64-3	3477	meat-like	[96]
6	2-mercapto-3-butanol	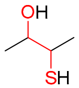	37887-04-0	3502	meat-like	[96]
7	3-mercapto-2-butanone	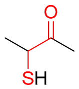	40789-98-8	3298	beefy aroma, roasty	[96]
8	1-mercapto-2-propanone	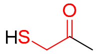	24653-75-6	3856	sulfuric, meat-like	[96]
9	3-mercaptopentan-2-one	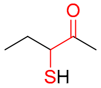	67633-97-0	3300	cured ham-like	[96]
10	2-acetylthiophene	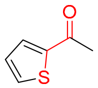	88-15-3	-	beefy aroma	[53]

Continued

No.	Compound	Molecular structure	CAS No.	FEMA No.	Odor description	Refs.
11	2-acetylthiazole	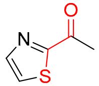	24295-03-2	3328	roasty beefy aroma	[53]
12	2-acetyl-2-thiazoline	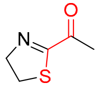	29926-41-8	3817	roasty, meet-like	[53]
13	1,3-dithiolane	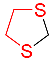	4829-04-3	-	roasted meat	[96]
14	2-methyl-3-tetrahydrofuranthiol	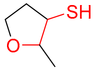	57124-87-5	3787	meet-like	[96]
15	4-mercapto-2-pentanone	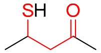	92585-08-5	4157	grapefruit-like, fruity	[98]
16	4-mercapto-4-methyl-2-pentanone	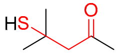	19872-52-7	3997	blackcurrant-like	[226]
17	2-mercapto-4-heptanone	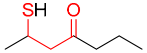	1006684-15-6	-	vegetables, onion-like	[95]
18	4-mercapto-2-heptanol	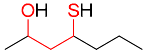	1006684-21-4	-	grapefruit-like, onion-like	[10]
19	methyl 3-(methylthio)propionate	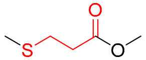	13532-18-8	2720	cabbage-like, earthy	[226]
20	3-mercapto-1-hexanol	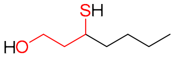	51755-83-0	3850	grapefruit-like	[10]
21	methionol	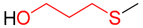	505-10-2	3415	cooked potato-like	[59]
22	4-mercapto-2-pentanol	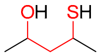	33959-24-9	-	onion-like	[10]
23	2-mercapto-4-heptanol	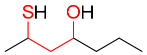	1006684-20-3	4733	vegetables, cooked onion-like	[94]

Continued

No.	Compound	Molecular structure	CAS No.	FEMA No.	Odor description	Refs.
24	methional	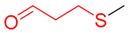	3268-49-3	2747	potato-like, cooked potato-like	[226]
25	3-mercapto-3-methyl-1-butanol	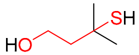	34300-94-2	3854	sweet, onion-like	[10]
26	3-mercaptohexyl acetate	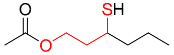	136954-20-6	3851	blackcurrant-like, grapefruit-like	[59]
27	1-propanethiol	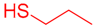	107-03-9	3521	onion-like, durian-like	[50]
28	dipropyl disulfide	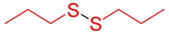	629-19-6	3228	onion-like, garlic-like	[227]
29	dipropyl trisulfide	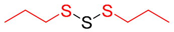	6028-61-1	3276	onion-like, garlic-like	[227]
30	methyl propyl disulfide	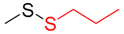	2179-60-4	3201	green onion, garlic-like	[227]
31	allyl mercaptan	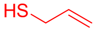	870-23-5	2035	garlic-like	[228]
32	allyl methyl sulfide	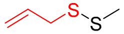	2179-58-0	3127	garlic-like	[228]
33	diallyl sulfide	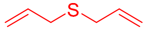	592-88-1	2042	onion-like, garlic-like, vegetables	[229]
34	diallyl disulfide	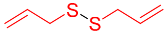	2179-57-9	2028	onion-like, garlic-like	[51]
35	diallyl trisulfide	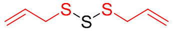	2050-87-5	3265	garlic-like	[230]
36	allyl methyl trisulfide	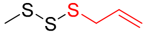	34135-85-8	3253	onion-like, garlic-like	[230]
37	allyl propyl disulfide	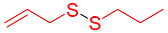	2179-59-1	4073	garlic-like, onion-like	[96]
38	2-furanmethanethiol	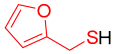	98-02-2	2493	roasty, coffee-like, meat-like	[14]

Continued

No.	Compound	Molecular structure	CAS No.	FEMA No.	Odor description	Refs.
39	furfuryl isopropyl sulfide	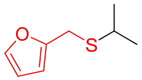	1883-78-9	3161	coffee-like	[231]
40	ethyl 3-(furfurylthio)propionate	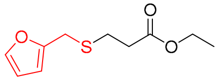	94278-27-0	3674	coffee-like, roasty	[231]
41	2-((methyldithio)methyl)furan	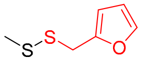	57500-00-2	3362	vegetables, coffee-like, sulfuric	[59]
42	bis(2-furanylmethyl) disulfide	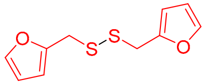	4437-20-1	3146	coffee-like, roasty, meat-like	[59]
43	*S*-furfuryl thioacetate	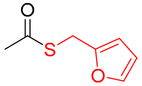	13678-68-7	3162	coffee-like, roasty, meat-like	[231]
44	*S*-furfuryl propanethioate	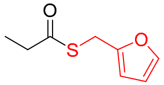	59020-85-8	3347	coffee-like, meat-like	[231]

References

[2] Dunkel A., Steinhaus M., Kotthoff M., et al. Nature's Chemical Signatures in Human Olfaction: A Foodborne Perspective for Future Biotechnology [J]. *Angew. Chem. Int. Ed.*, **2014**, 53(28): 7124–7143.

[7] Baoguo Sun F. D., Fuping Zheng,Yuping Liu. Relationship of Molecular Structure of Sulfur-containing Flavoring Compounds to Meat Flavors [J]. *Fine Chem.*, **2001**, (08): 456–460.

[10] Polster J., Schieberle P. Structure Odor Correlations in Homologous Series of Mercaptoalkanols [J]. *J. Agric. Food Chem.*, **2017**, 65(21): 4329–4340.

[14] Zhang G., Xiao P., Yuan M., et al. Roles of sulfur-containing compounds in fermented beverages with 2-furfurylthiol as a case example [J]. *Front. Nutr.*, **2023**, Volume 10 - **2023**.

[44] Zhao X., Wang H., Yin S., et al. Methanesulfenylation of mercaptans and β-dicarbonyls with DMSO [J]. *J. Sulfur Chem.*, **2024**, 45(3): 330–345.

[45] Gasior R., Wojtycza K., Majcher M., et al. Key Aroma Compounds in Roasted White Koluda Goose [J]. *J. Agric. Food Chem.*, **2021**, 69(21): 5986–5996.

[46] Sen A., Grosch W. Synthesis of six deuterated sulfur containing odorants to be used as internal standards in quantification assays [J]. *Z. Lebensm. Unters. Forsch.*, **1991**, 192(6): 541–547.

[50] Li J.-X., Schieberle P., Steinhaus M. Development of stable isotope dilution assays for the quantitation of the food odorants hydrogen sulphide, methanethiol, ethanethiol, and propane-1-thiol and application to durian (*Durio zibethinus* L.) pulp [J]. *Eur. Food Res. Technol.*, **2017**, 243(1): 69–79.

[51] Bi J., Yang Z., Li Y., et al. Effects of different cooking methods on volatile flavor compounds in garlic [J]. *Int. J. Gastron. Food Sci.*, **2023**, 31.

[53] Shakoor A., Pei Z., Al-Dalali S., et al. Unveiling improval changes in fried chicken flavor by glucose-curing treatment based on molecular sensory science [J]. *J. Food Compos. Anal.*, **2023**, 124: 105697.

[59] Wang H., Yang P., Liu C., et al. Characterization of key odor-active compounds in thermal reaction beef flavoring by SGC× GC-O-MS, AEDA, DHDA, OAV and quantitative measurements [J]. *J. Food Compos. Anal.*, **2022**, 114: 104805.

[94] Riegel A. D., Kiske C., Dudko V., et al. Absolute Configurations and Sensory Properties of the Stereoisomers of a Homologous Series (C6-C10) of 2-Mercapto-4-alkanols [J]. *J. Agric. Food Chem.*, **2020**, 68(9): 2738–2746.

[95] Kiske C., Riegel A. D., Hopf R., et al. Determination of the Absolute Configurations and Sensory Properties of the Enantiomers of a Homologous Series (C6-C10) of 2-Mercapto-4-alkanones [J]. *J. Agric. Food Chem.*, **2019**, 67(4): 1187–1196.

[96] Sun B. Chemistry of Sulfur-Containing Flavor Compounds [M]. Ke xue chu ban she: Beijing, China, **2007**.

[98] Wakabayashi M., Wakabayashi H., Noerenberg S., et al. Comparison of odour thresholds and odour qualities of the enantiomers of 4-mercapto-2-alkanones and 4-acetylthio-2-alkanones [J]. *Flavour Fragr. J.*, **2015**, 30(2): 171–178.

[126] Tamura H., Fujita A., Steinhaus M., et al. Assessment of the Aroma Impact of Major Odor-Active Thiols in Pan-Roasted White Sesame Seeds by Calculation of Odor Activity Values [J]. *J. Agric. Food Chem.*, **2011**, 59(18): 10211–10218.
[127] Cerny C., Grosch W. Quantification of character-impact odour compounds of roasted beef [J]. *Z. Lebensm. Unters. Forsch.*, **1993**, 196(5): 417–422.
[128] Flaig M., Qi S. C., Wei G., et al. Characterisation of the key aroma compounds in a Longjing green tea infusion (*Camellia sinensis*) by the sensomics approach and their quantitative changes during processing of the tea leaves [J]. *Eur. Food Res. Technol.*, **2020**, 246(12): 2411–2425.
[129] Fricke K., Schieberle P. Characterization of the Key Aroma Compounds in a Commercial Milk Chocolate by Application of the Sensomics Approach [J]. *J. Agric. Food Chem.*, **2020**, 68(43): 12086–12095.
[130] Tran T. K. L., Gillich E., André A., et al. Decoding the Aroma Gap: Sensomics-Based Characterization of Key Odorants in the Plant-Based Egg and Chicken Egg [J]. *J. Agric. Food Chem.*, **2025**, 73(22): 13832–13842.
[131] Ortner E., Granvogl M., Schieberle P. Elucidation of Thermally Induced Changes in Key Odorants of White Mustard Seeds (*Sinapis alba* L.) and Rapeseeds (*Brassica napus* L.) Using Molecular Sensory Science [J]. *J. Agric. Food Chem.*, **2016**, 64(43): 8179–8190.
[132] Lapsongphon N., Yongsawatdigul J., Cadwallader K. R. Identification and Characterization of the Aroma-Impact Components of Thai Fish Sauce [J]. *J. Agric. Food Chem.*, **2015**, 63(10): 2628–2638.
[133] Dach A., Schieberle P. Characterization of the Key Aroma Compounds in a Freshly Prepared Oat (*Avena sativa* L.) Pastry by Application of the Sensomics Approach [J]. *J. Agric. Food Chem.*, **2021**, 69(5): 1578–1588.
[134] Poehlmann S., Schieberle P. Characterization of the aroma signature of styrian pumpkin seed oil (Cucurbita pepo subsp. pepo var. Styriaca) by molecular sensory science [J]. *J. Agric. Food Chem.*, **2013**, 61(12): 2933–2942.
[135] Rychlik M., Bosset J. O. Flavour and off-flavour compounds of Swiss Gruyère cheese. Identification of key odorants by quantitative instrumental and sensory studies [J]. *Int. Dairy J.*, **2001**, 11(11): 903–910.
[136] Kubícková J., Grosch W. Quantification of potent odorants in camembert cheese and calculation of their odour activity values [J].*Int. Dairy J.*, **1998**, 8(1): 17–23.
[137] Masanetz C., Guth H., Grosch W. Fishy and hay-like off-flavours of dry spinach [J]. *Z. Lebensm. Unters. Forsch. A*, **1998**, 206(2): 108–113.
[138] Chetschik I., Granvogl M., Schieberle P. Quantitation of Key Peanut Aroma Compounds in Raw Peanuts and Pan-Roasted Peanut Meal. Aroma Reconstitution and Comparison with Commercial Peanut Products [J]. *J. Agric. Food Chem.*, **2010**, 58(20): 11018–11026.
[139] Milo C., Grosch W. Changes in the Odorants of Boiled Salmon and Cod As Affected by the Storage of the Raw Material [J]. *J. Agric. Food Chem.*, **1996**, 44(8): 2366–2371.
[140] Masanetz C., Grosch W. Key odorants of parsley leaves (Petroselinum crispum [Mill.] Nym. ssp. crispum) by Odour–activity values [J]. *Flavour Fragr. J.*, **1998**, 13(2): 115–124.

[141] Masanetz C., Grosch W. Hay-like off-flavour of dry parsley [J]. *Z. Lebensm. Unters. Forsch. A*, **1998**, 206(2): 114–120.
[142] Semmelroch P., Grosch W. Studies on Character Impact Odorants of Coffee Brews [J]. *J. Agric. Food Chem.*, **1996**, 44(2): 537–543.
[143] Kerler J., Grosch W. Character impact odorants of boiled chicken: changes during refrigerated storage and reheating [J]. *Z. Lebensm. Unters. Forsch. A*, **1997**, 205(3): 232–238.
[144] Milo C., Grosch W. Potent odorants in boiled cod as affected by the storage of raw material [J]. *Flavor and lipid chemistry of seafoods*; ACS Publications: Washington, DC, USA,, **1998**.
[145] Mall V., Schieberle P. Evaluation of Key Aroma Compounds in Processed Prawns (Whiteleg Shrimp) by Quantitation and Aroma Recombination Experiments [J]. *J. Agric. Food Chem.*, **2017**, 65(13): 2776–2783.
[146] Flaig M., Qi S., Wei G., et al. Characterization of the Key Odorants in a High-Grade Chinese Green Tea Beverage (Camellia sinensis; Jingshan cha) by Means of the Sensomics Approach and Elucidation of Odorant Changes in Tea Leaves Caused by the Tea Manufacturing Process [J]. *J. Agric. Food Chem.*, **2020**, 68(18): 5168–5179.
[147] Li J.-X., Schieberle P., Steinhaus M. Insights into the Key Compounds of Durian (Durio zibethinus L. 'Monthong') Pulp Odor by Odorant Quantitation and Aroma Simulation Experiments [J]. *J. Agric. Food Chem.*, **2017**, 65(3): 639–647.
[148] Matheis K., Granvogl M. Characterisation of the key aroma compounds in commercial native cold-pressed rapeseed oil by means of the Sensomics approach [J]. *Eur. Food Res. Technol.*, **2016**, 242(9): 1565–1575.
[149] Zhai X., Granvogl M. Characterization of the Key Aroma Compounds in Two Differently Dried Toona sinensis (A. Juss.) Roem. by Means of the Molecular Sensory Science Concept [J]. *J. Agric. Food Chem.*, **2019**, 67(35): 9885–9894.
[150] Majcher M. A., Olszak-Ossowska D., Szudera-Kończal K., et al. Formation of Key Aroma Compounds during Preparation of Pumpernickel Bread [J]. *J. Agric. Food Chem.*, **2020**, 68(38): 10352–10360.
[151] Kelley L. E., Cadwallader K. R. Identification and Quantitation of Potent Odorants in Spearmint Oils [J]. *J. Agric. Food Chem.*, **2018**, 66(10): 2414–2421.
[152] Wu T., Cadwallader K. R. Identification of Characterizing Aroma Components of Roasted Chicory "Coffee" Brews [J]. *J. Agric. Food Chem.*, **2019**, 67(50): 13848–13859.
[153] Jeleń H., Majcher M., Ginja A., et al. Determination of compounds responsible for tempeh aroma [J]. *Food Chem.*, **2013**, 141(1): 459–465.
[154] Averbeck M., Schieberle P. Influence of different storage conditions on changes in the key aroma compounds of orange juice reconstituted from concentrate [J]. *Eur. Food Res. Technol.*, **2011**, 232(1): 129–142.
[155] Fritsch H. T., Schieberle P. Identification Based on Quantitative Measurements and Aroma Recombination of the Character Impact Odorants in a Bavarian Pilsner-type Beer [J]. *J. Agric. Food Chem.*, **2005**, 53(19): 7544–7551.
[156] Guth H. Quantitation and Sensory Studies of Character Impact Odorants of Different White Wine Varieties [J]. *J. Agric. Food Chem.*, **1997**, 45(8): 3027–3032.

[157] Wang J., Hu Y., Guan Z., et al. Dynamic changes in aroma of large-leaf yellow tea during the whole processing at an industrial scale: From fresh leaf to finished tea [J]. *Food Chem. X*, **2025**, 28: 102628.

[158] Zhang Z., Liang M., Liu B., et al. Characterization of the key odorants of ripe Capparis spinosa L. fruit and changes in concentrations after storage by means of molecular sensory science [J]. *Food Chem.*, **2023**, 403: 134383.

[159] Matheis K., Granvogl M. Characterization of Key Odorants Causing a Fusty/Musty Off-Flavor in Native Cold-Pressed Rapeseed Oil by Means of the Sensomics Approach [J]. *J. Agric. Food Chem.*, **2016**, 64(43): 8168–8178.

[160] Schmidberger P. C., Schieberle P. Characterization of the Key Aroma Compounds in White Alba Truffle (*Tuber magnatum pico*) and Burgundy Truffle (*Tuber uncinatum*) by Means of the Sensomics Approach [J]. *J. Agric. Food Chem.*, **2017**, 65(42): 9287–9296.

[161] Zhou J., He C., Qin M., et al. Characterizing and Decoding the Effects of Different Fermentation Levels on Key Aroma Substances of Congou Black Tea by Sensomics [J]. *J. Agric. Food Chem.*, **2023**, 71(40): 14706–14719.

[162] Zhai X., Granvogl M. Elucidation of the Impact of Different Drying Methods on the Key Odorants of Toona sinensis (A. Juss.) Roem. Using the Sensomics Approach [J]. *J. Agric. Food Chem.*, **2020**, 68(29): 7697–7709.

[163] Wang H., Bi H., Wang J., et al. Key odorants of 'Keemun Aroma' and their changes during the preliminary processing of Keemun BLack Tea (*Camellia sinensis*) [J]. *J. Food Compos. Anal.*, **2024**, 132: 106254.

[164] Majcher M. A., Myszka K., Kubiak J., et al. Identification of key odorants of fried cottage cheese and contribution of Galactomyces geotrichum MK017 to the formation of 2-phenylethanol and related rose-like aroma compounds [J]. *Int. Dairy J.*, **2014**, 39(2): 324–329.

[165] Burdack-Freitag A., Schieberle P. Characterization of the key odorants in raw Italian hazelnuts (*Corylus avellana* L. var. Tonda Romana) and roasted hazelnut paste by means of molecular sensory science [J].*J. Agric. Food Chem.*, **2012**, 60(20): 5057–5064.

[166] Kiefl J., Pollner G., Schieberle P. Sensomics Analysis of Key Hazelnut Odorants (*Corylus avellana* L. ‘Tonda Gentile’) Using Comprehensive Two-Dimensional Gas Chromatography in Combination with Time-of-Flight Mass Spectrometry (GC×GC-TOF-MS) [J]. *J. Agric. Food Chem.*, **2013**, 61(22): 5226–5235.

[167] Schieberle P. Odour-active compounds in moderately roasted sesame [J]. *Food Chem.*, **1996**, 55(2): 145–152.

[168] Münch P., Schieberle P. Quantitative Studies on the Formation of Key Odorants in Thermally Treated Yeast Extracts Using Stable Isotope Dilution Assays [J].*J. Agric. Food Chem.*, **1998**, 46(11): 4695–4701.

[169] Semmelroch P., Laskawy G., Blank I., et al. Determination of potent odourants in roasted coffee by stable isotope dilution assays [J]. *Flavour Fragr. J.*, **1995**, 10(1): 1–7.

[170] Söllner K., Schieberle P. Decoding the Key Aroma Compounds of a Hungarian-Type Salami by Molecular Sensory Science Approaches [J]. *J. Agric. Food Chem.*, **2009**, 57(10): 4319–4327.

[171] Schieberle, P. Important Odorants in Roasted White and Black Sesame Seeds. In Olfaction and Taste XI; Kurihara, K., Suzuki, N., Ogawa, H., Eds.; Springer: Tokyo, Japan, **1994**; pp. 263–267.

[172] Pavez C., Agosin E., Steinhaus M. Odorant Screening and Quantitation of Thiols in Carmenere Red Wine by Gas Chromatography–Olfactometry and Stable Isotope Dilution Assays [J]. *J. Agric. Food Chem.*, **2016**, 64(17): 3417–3421.

[173] Lopez J., Kerley T., Jenkinson L., et al. Odorants from the Thermal Treatment of Hydrolyzed Mushroom Protein and Cysteine Enhance Saltiness Perception [J]. *J. Agric. Food Chem.*, **2019**, 67(41): 11444–11453.

[174] Schmidberger P. C., Schieberle P. Changes in the Key Aroma Compounds of Raw Shiitake Mushrooms (*Lentinula edodes*) Induced by Pan-Frying As Well As by Rehydration of Dry Mushrooms [J]. *J. Agric. Food Chem.*, **2020**, 68(15): 4493–4506.

[175] Kreissl J., Schieberle P. Characterization of Aroma-Active Compounds in Italian Tomatoes with Emphasis on New Odorants [J]. *J. Agric. Food Chem.*, **2017**, 65(25): 5198–5208.

[176] Marcinkowska M., Frank S., Steinhaus M., et al. Key Odorants of Raw and Cooked Green Kohlrabi (*Brassica oleracea var. gongylodes* L.) [J]. *J. Agric. Food Chem.*, **2021**, 69(41): 12270–12277.

[177] Szudera-Kończal K., Myszka K., Kubiak P., et al. Analysis of the Ability to Produce Pleasant Aromas on Sour Whey and Buttermilk By-Products by Mold Galactomyces geotrichum: Identification of Key Odorants [J]. *Molecules*, **2021**, 26(20): 6239.

[178] Nguyen T. H., Munafo J. P., Jr. Characterization of Important Odorants in Dried Lobster Mushrooms [J]. *J. Agric. Food Chem.*, **2024**, 72(45): 25251–25260.

[179] Wagner J., Granvogl M., Schieberle P. Characterization of the Key Aroma Compounds in Raw Licorice (*Glycyrrhiza glabra* L.) by Means of Molecular Sensory Science [J]. *J. Agric. Food Chem.*, **2016**, 64(44): 8388–8396.

[180] Schoenauer S., Schieberle P. Characterization of the Key Aroma Compounds in the Crust of Soft Pretzels by Application of the Sensomics Concept [J]. *J. Agric. Food Chem.*, **2019**, 67(25): 7110–7119.

[181] Lasekan O., Dabaj F., Muniandy M., et al. Characterization of the key aroma compounds in three types of bagels by means of the sensomics approach [J]. *BMC Chem.*, **2021**, 15(1): 16.

[182] Sahin B., Schieberle P. Characterization of the Key Aroma Compounds in Yeast Dumplings by Means of the Sensomics Concept [J].*J. Agric. Food Chem.*, **2019**, 67(10): 2973–2979.

[183] Schmitt R., Cappi M., Pollner G., et al. Key Aroma Compounds in *Lippia dulcis* (Dushi Button) [J]. *J. Agric. Food Chem.*, **2018**, 66(10): 2410–2413.

[184] Moore A. J., Wickramasinghe P. C. K., Munafo Jr. J. P. Key Odorants from Daldinia childiae [J]. *Flavour Fragr. J.*, **2020**, 35(6): 722–733.

[185] Amann L. S., Frank O., Dawid C., et al. The Sensory-Directed Elucidation of the Key Tastants and Odorants in Sourdough Bread Crumb [J]. *Foods*, **2022**, 11(15): 2325.

[186] Nicolotti L., Mall V., Schieberle P. Characterization of Key Aroma Compounds in a Commercial Rum and an Australian Red Wine by Means of a New Sensomics-Based

Expert System (SEBES)—An Approach To Use Artificial Intelligence in Determining Food Odor Codes [J]. *J. Agric. Food Chem.*, **2019**, 67(14): 4011–4022.

[187] Majcher M. A., Jeleń H. H. Key Odorants of Oscypek, a Traditional Polish Ewe's Milk Cheese [J]. *J. Agric. Food Chem.*, **2011**, 59(9): 4932–4937.

[188] Steinhaus P., Schieberle P. Characterization of the Key Aroma Compounds in Soy Sauce Using Approaches of Molecular Sensory Science [J]. *J. Agric. Food Chem.*, **2007**, 55(15): 6262–6269.

[189] Steinhaus M., Sinuco D., Polster J., et al. Characterization of the Key Aroma Compounds in Pink Guava (*Psidium guajava* L.) by Means of Aroma Re-engineering Experiments and Omission Tests [J].*J. Agric. Food Chem.*, **2009**, 57(7): 2882–2888.

[190] Kirchhoff E., Schieberle P. Determination of Key Aroma Compounds in the Crumb of a Three-Stage Sourdough Rye Bread by Stable Isotope Dilution Assays and Sensory Studies [J]. *J. Agric. Food Chem.*, **2001**, 49(9): 4304–4311.

[191] Kirchhoff E., Schieberle P. Quantitation of Odor-Active Compounds in Rye Flour and Rye Sourdough Using Stable Isotope Dilution Assays [J]. *J. Agric. Food Chem.*, **2002**, 50(19): 5378–5385.

[192] Buettner A., Schieberle P. Evaluation of Key Aroma Compounds in Hand-Squeezed Grapefruit Juice (*Citrus paradisi Macfayden*) by Quantitation and Flavor Reconstitution Experiments [J]. *J. Agric. Food Chem.*, **2001**, 49(3): 1358–1363.

[193] Buettner A., Schieberle P. Evaluation of Aroma Differences between Hand-Squeezed Juices from Valencia Late and Navel Oranges by Quantitation of Key Odorants and Flavor Reconstitution Experiments [J]. *J. Agric. Food Chem.*, **2001**, 49(5): 2387–2394.

[194] Zehentbauer G., Grosch W. Crust Aroma of Baguettes I. Key Odorants of Baguettes Prepared in Two Different Ways [J]. *J. Cereal Sci.*, **1998**, 28(1): 81–92.

[195] Milo C., Grosch W. Changes in the odorants of boiled trout (*Salmo fario*) as affected by the storage of the raw material [J]. *J. Agric. Food Chem.*, **1993**, 41(11): 2076–2081.

[196] Rychlik M., Grosch W. Identification and Quantification of Potent Odorants Formed by Toasting of Wheat Bread [J]. *LWT*, **1996**, 29(5): 515–525.

[197] Kerler, J.; Grosch, W. Odorants Contributing to Warmed-Over Flavor (WOF) of Refrigerated Cooked Beef [J]. *J. Food Sci.*, **1996**, 61(6): 1271–1275.

[198] Fuhrmann E., Grosch W. Character impact odorants of the apple cultivars Elstar and Cox Orange [J]. *Nahrung*, **2002**, 46(3): 187–193.

[199] Frank S., Wollmann N., Schieberle P., et al. Reconstitution of the Flavor Signature of Dornfelder Red Wine on the Basis of the Natural Concentrations of Its Key Aroma and Taste Compounds [J]. *J. Agric. Food Chem.*, **2011**, 59(16): 8866–8874.

[200] Preininger M., Grosch W. Evaluation of Key Odorants of the Neutral Volatiles of Emmentaler Cheese by the Calculation of Odour Activity Values [J]. *LWT*, **1994**, 27(3): 237–244.

[201] Schaller T., Schieberle P. Quantitation of Key Aroma Compounds in Fresh, Raw Ginger (*Zingiber officinale* Roscoe) from China and Roasted Ginger by Stable Isotope Dilution Assays and Aroma Profiling by Recombination Experiments [J]. *J. Agric. Food Chem.*, **2020**, 68(51): 15284–15291.

[202] Schlegel K., Ortner E., Buettner A., et al. Contribution of S. xylosus and L. sakei ssp. carnosus Fermentation to the Aroma of Lupin Protein Isolates [J]. *Foods*, **2021**, 10(6): 1257.
[203] Marcq P., Schieberle P. Characterization of the Key Aroma Compounds in a Commercial Amontillado Sherry Wine by Means of the Sensomics Approach [J]. *J. Agric. Food Chem.*, **2015**, 63(19): 4761–4770.
[204] Dein M., Moore A., Ricketts C., et al. Characterization of Odorants in Chardonnay Marc Skins [J]. *J. Agric. Food Chem.*, **2021**, 69(41): 12262–12269.
[205] Dein M., Kerley T., Munafo J. P., Jr. Characterization of Odorants in a 10-Year-Old Riesling Wine [J]. *J. Agric. Food Chem.*, **2021**, 69(38): 11372–11381.
[206] Christlbauer M., Schieberle P. Evaluation of the Key Aroma Compounds in Beef and Pork Vegetable Gravies a la Chef by Stable Isotope Dilution Assays and Aroma Recombination Experiments [J]. *J. Agric. Food Chem.*, **2011**, 59(24): 13122–13130.
[207] Yang Y., Cadwallader K. R. Comparison of Key Odorants and Sensory Properties of Peanut Butter and Sunflower Seed Butter [J]. *J. Agric. Food Chem.*, **2025**, 73(18): 11235–11247.
[208] Lasekan O., Dabaj F. Characterization of the Key Aroma Constituents in Fry Breads by Means of the Sensomics Concept [J]. *Foods*, **2020**, 9(8): 1129.
[209] Buttara M., Intarapichet K.-O., Cadwallader K. R. Characterization of potent odorants in Thai chempedak fruit (*Artocarpus integer* Merr.), an exotic fruit of Southeast Asia [J]. *Food Res. Int.*, **2014**, 66: 388–395.
[210] Seyfried C., Granvogl M. Characterization of the Key Aroma Compounds in Two Commercial Dark Chocolates with High Cocoa Contents by Means of the Sensomics Approach [J]. *J. Agric. Food Chem.*, **2019**, 67(20): 5827–5837.
[211] Ac-Pangan M. F., Engeseth N. J., Cadwallader K. R. Identification of Important Aroma Components and Sensory Profiles of Minimally Processed (Unroasted) and Conventionally Roasted Dark Chocolates [J]. *J. Agric. Food Chem.*, **2023**, 71(25): 9856–9867.
[212] Munafo J. P., Jr., Didzbalis J., Schnell R. J., et al. Insights into the Key Aroma Compounds in Mango (*Mangifera indica* L. 'Haden') Fruits by Stable Isotope Dilution Quantitation and Aroma Simulation Experiments [J]. *J. Agric. Food Chem.*, **2016**, 64(21): 4312–4318.
[213] Matheis K., Granvogl M. Unraveling of the Fishy Off-Flavor in Steam-Treated Rapeseed Oil Using the Sensomics Concept [J]. *J. Agric. Food Chem.*, **2019**, 67(5): 1484–1494.
[214] Ruisinger B., Schieberle P. Characterization of the Key Aroma Compounds in Rape Honey by Means of the Molecular Sensory Science Concept [J]. *J. Agric. Food Chem.*, **2012**, 60(17): 4186–4194.
[215] Frauendorfer F., Schieberle P. Changes in Key Aroma Compounds of Criollo Cocoa Beans During Roasting [J].*J. Agric. Food Chem.*, **2008**, 56(21): 10244–10251.
[216] Frauendorfer F., Schieberle P. Identification of the Key Aroma Compounds in Cocoa Powder Based on Molecular Sensory Correlations [J]. *J. Agric. Food Chem.*, **2006**, 54(15): 5521–5529.

[217] Brendel S., Hofmann T., Granvogl M. Characterization of Key Aroma Compounds in Pellets of Different Hop Varieties (*Humulus lupulus* L.) by Means of the Sensomics Approach [J]. *J. Agric. Food Chem.*, **2019**, 67(43): 12044–12053.

[218] André A., Tran T. K. L., Gillich E., et al. First Quantitation of Key Odor-Active Thiols in Flowers of Different Hemp Cultivars Using HPLC-MS/MS [J]. *J. Agric. Food Chem.*, **2026**, 74(1): 1379–1387.

[219] George J., Nguyen T. T. L., Sanewski G., et al. Stable isotope dilution assay and HS-SPME-GCMS quantification of key aroma volatiles of Australian pineapple (*Ananas comosus*) cultivars [J]. *Food Chem.*, **2024**, 455: 139956.

[220] Neiens S. D., Steinhaus M. Odor-Active Compounds in the Special Flavor Hops Huell Melon and Polaris [J]. *J. Agric. Food Chem.*, **2018**, 66(6): 1452–1460.

[221] Rigou P., Triay A., Razungles A. Influence of volatile thiols in the development of blackcurrant aroma in red wine [J]. *Food Chem.*, **2014**, 142: 242–248.

[222] Kerley T., Munafo J. P., Jr. Changes in Tennessee Whiskey Odorants by the Lincoln County Process [J].*J. Agric. Food Chem.*, **2020**, 68(36): 9759–9767.

[223] Sinuco D. C., Steinhaus M., Schieberle P., et al. Changes in odour-active compounds of two varieties of Colombian guava (*Psidium guajava* L.) during ripening [J]. *Eur. Food Res. Technol.*, **2010**, 230(6): 859–864.

[224] Tran T. K. L., Salvatore I., Geller J., et al. Molecular Aroma Composition of Vanilla Beans from Different Origins [J]. *J. Agric. Food Chem.*, **2024**, 72(34): 19120–19130.

[225] Reiners J., Grosch W. Odorants of Virgin Olive Oils with Different Flavor Profiles [J]. *J. Agric. Food Chem.*, **1998**, 46(7): 2754–2763.

[226] Niu Y., Deng J., Xiao Z., et al. Characterization of the major aroma-active compounds in peach (*Prunus persica* L. Batsch) by gas chromatography-olfactometry, flame photometric detection and molecular sensory science approaches [J]. *Food Res. Int.*, **2021**, 147.

[227] Liu B., Xu S., Dong Z., et al. Characterization of Key Odorants in Scallion Pancake and Investigation on Their Changes during Storage [J]. *Molecules*, **2021**, 26(24).

[228] Zhang Y., Sun Y., Song H. Variation in Volatile Flavor Compounds of Cooked Mutton Meatballs during Storage [J]. *Foods*, **2021**, 10(10).

[229] Jiménez-Amezcua I., Sanz M., Díez-Municio M., et al. Changes in the bioactive content and aroma profile of aged garlic with processing conditions [J]. *Lwt*, **2025**, 227.

[230] Yang P., Song H., Wang L., et al. Characterization of Key Aroma-Active Compounds in Black Garlic by Sensory-Directed Flavor Analysis [J]. *J. Agric. Food Chem.*, **2019**, 67(28): 7926–7934.

[231] Sun B. Handbook of Food Flavors [M]. China Petrochemical Press: Beijing, China, **2004**.
